# COVID-19 vaccination and birth outcomes of 186,990 women vaccinated before pregnancy: an England-wide cohort study

**DOI:** 10.1016/j.lanepe.2024.101025

**Published:** 2024-08-13

**Authors:** Arun K. Suseeladevi, Rachel Denholm, Matthew Retford, Elena Raffetti, Christy Burden, Katherine Birchenall, Victoria Male, Venetia Walker, Christopher Tomlinson, Angela M. Wood, Luisa Zuccolo, CVD-COVID-UK/COVID-IMPACT Consortium

**Affiliations:** a Population Health Sciences, Bristol Medical School, University of Bristol, Bristol, UK; b NIHR Bristol Biomedical Research Centre, University of Bristol, Bristol, UK; c MRC Integrative Epidemiology Unit, University of Bristol, Bristol, UK; d Health Data Research UK South-West, Bristol, UK; e Health Data Research UK, London, UK; f British Heart Foundation Cardiovascular Epidemiology Unit, Department of Public Health and Primary Care, University of Cambridge, Cambridge, UK; g Victor Phillip Dahdaleh Heart and Lung Research Institute, University of Cambridge, Cambridge, UK; h Department of Global Public Health, Karolinska Institutet, Stockholm, Sweden; i Translational Health Sciences, Bristol Medical School, University of Bristol, Bristol, UK; j Department of Metabolism, Digestion and Reproduction, Imperial College London, London, UK; k Academic Womens Health Unit, Bristol Medical School, University of Bristol, Bristol, UK; l Institute of Health Informatics, University College London, London, UK; m University College London Hospitals Biomedical Research Centre, University College London, London, UK; n British Heart Foundation Data Science Centre, Health Data Research UK, London, UK; o Health Data Science Centre, Human Technopole, Milan, Italy

**Keywords:** COVID-19 vaccination, Pregnancy, Preterm, Stillbirth, Venous thromboembolism, Electronic health records

## Abstract

**Background:**

COVID-19 vaccination in pregnancy is recommended by the World Health Organisation as effective and safe. However, there remains a lack of robust evidence to inform vaccination choices for women of childbearing potential in relation to their future pregnancies. Here we investigated the association between starting a course of COVID-19 vaccination before pregnancy and birth outcomes.

**Methods:**

We analysed England-wide linked electronic health records for all pregnancies reaching at least 24 weeks gestation between 25th May 2021 and 28th October 2022. We estimated incidence rates and hazard ratios for birth and pregnancy outcomes by pre-pregnancy COVID-19 vaccination status.

**Findings:**

Based on 186,990 women, compared to starting a pregnancy unvaccinated, receiving COVID-19 vaccination within 12 months before pregnancy was associated with lower risks of very and extremely preterm birth and small-for-gestational age in term babies for any vaccine type (adjusted hazard ratio and 95% confidence interval: 0.74 [0.63, 0.88] and 0.94 [0.88, 1.00], respectively), and lower stillbirth risk in those receiving an mRNA vaccine (0.72 [0.52, 1.00]). Incidence of venous thromboembolism during pregnancy was higher amongst women receiving a viral-vector, but not an mRNA vaccine (1.54 [1.10, 2.16] and 1.02 [0.70, 1.50], respectively). Results were generally consistent for different dose regimens and across sensitivity analyses.

**Interpretation:**

We found evidence that pregnancies starting within 12 months from a first COVID-19 vaccination, compared to those in unvaccinated women, experienced fewer adverse birth outcomes, overall or in selected subgroups of the general population, accounting for potential confounders. An mRNA vaccine should be preferred to a viral-vector vaccine, to minimise safety issues, but where the latter is the only choice, it is still to be preferred to starting a pregnancy unvaccinated. The venous thromboembolism risk of the viral-vector vaccine was substantially lower compared to that attributable to SARS-CoV-2 infection in pregnancy or to commonly used medications such as hormone replacement therapy and oral contraceptives in the non-pregnant population.

**Funding:**

UK 10.13039/501100000272National Institute for Health and Care Research (NIHR), 10.13039/100014013UKRI10.13039/501100000265Medical Research Council, UK Research and Innovation, The Alan Turing Institute, 10.13039/501100023699Health Data Research UK, the 10.13039/501100000276Department of Health and Social Care.

**Research in context**

**Evidence before this study**

We searched for prospective or retrospective epidemiological studies investigating the association of COVID-19 vaccination before pregnancy with pregnancy and birth outcomes published in any language up until February 20, 2024 (with no specified earliest date), in MEDLINE, Scientific Citation Index Expanded, and Embase using relevant terms ((‘conception’ OR ‘pregnan∗’ OR ‘gestation’) AND (‘preterm’ OR ‘premature’∗ OR ‘pregnancy’ ‘complication’ OR (‘adverse’ AND ‘birth’ AND ‘outcome∗’) OR ‘stillbirth’ OR (‘pregnan’∗ AND ‘loss’) OR (‘venous’ AND ‘thrombo∗’)) AND ((‘COVID-19’ OR ‘SARS-CoV-2’ OR ‘coronavirus’) AND (‘vaccin∗))) (n = 757). We found many primary reports and literature-based reviews reporting on the association of COVID-19 vaccination in pregnancy, demonstrating its safety in the short-, medium- and long-term with respect of a whole range of pregnancy, birth and neonatal outcomes, and confirming protection against COVID-19 disease severity (e.g. hospitalisation) and common adverse pregnancy outcomes that have been shown to be consequences of COVID-19 in pregnancy (e.g. preterm birth and stillbirth). However, only a handful of small or very small studies focussed on COVID-19 vaccination before pregnancy, yielding conflicting and widely imprecise estimates, ignoring rare and serious risks for women such as thrombotic events and failing to explore vaccine type-specific effects or whether maternal characteristics and medical history could confer increased vulnerability.

**Added value of this study**

We found that COVID-19 vaccination does not adversely affect birth outcomes in pregnancies starting after vaccination, and in fact it reduces the risk of extremely and very preterm birth and of small-for-gestational age in babies born after 36 weeks of gestation (and stillbirth in the case of the mRNA vaccine). All women and their infants are likely to benefit from pre-pregnancy COVID-19 vaccination (within a year from pregnancy start) irrespective of dose regimens, vaccine type, previous medical history and sociodemographic characteristics. Starting a course of a viral-vector vaccine within 12 months before pregnancy (generally 2 doses of which at least 1 before pregnancy) could be associated with higher venous thromboembolism risk in pregnancy. However, even if confirmed to be causal in future studies, the observed increase in risk would still be considerably smaller in magnitude than the venous thromboembolism risk attributable to SARS-CoV-2 infection in pregnancy or to commonly used medications such as hormone replacement therapy and oral contraceptives in the non-pregnant population.

**Implications of all the available evidence**

The main public health implication of all the available evidence is that COVID-19 vaccination is to be recommended to all women of reproductive age, especially those intending to become pregnant within a year. The second implication is that, should causality of the observed associations be confirmed, mRNA vaccines should be preferred to a viral vector vaccine, but where the latter is the only choice, it is still to be preferred to starting a pregnancy unvaccinated against COVID-19.

## Introduction

Infection with SARS-CoV-2 during pregnancy increases the risk of adverse outcomes for mothers and fetuses.^1^ The World Health Organisation therefore recommends COVID-19 vaccination for pregnant women,^2^ as it has been proven to be effective in reducing these risks.^3^, ^4^, ^5^, ^6^ Vaccines in pregnancy have also been found to be safe, with international systematic reviews showing no increased risk of miscarriage,^7^ preterm birth, stillbirth or small-for-gestational age,^4^ and a reduced risk of severe symptoms, complications, and death from COVID-19.^5^^,^^8^

Having been systematically excluded from COVID-19 vaccine trials, pregnant women in the UK and the US were not initially prioritised as a high-risk group at the start of vaccine campaigns (December 2020/January 2021). It was not until July and August 2021 that vaccination was explicitly recommended, and from December 2021 prioritised.^9^, ^10^, ^11^ Despite the widely known risks of severe illness and death from COVID-19 in pregnancy, women of reproductive age still experience vaccine hesitancy, especially those from minority ethnic groups.^12^ National surveillance data show approximately a quarter of women in England of reproductive age remain unvaccinated, possibly due to concerns about the consequences on future pregnancies, and confirm ethnicity and (younger) age as predictors of lower vaccine uptake.^13^ An increased risk of venous thromboembolism observed within two weeks of receiving the viral-vector vaccine fueled additional concerns.^14^

The missing piece of the jigsaw to inform both prioritization strategies and public health messaging for vaccination campaigns in women of reproductive age is direct evidence that vaccines offer protection without increasing pregnancy risks. However, to date there are no large-scale studies of medium-to long-term risks of vaccination prior to conception on subsequent birth outcomes.

Here, we aimed to investigate the association of starting a course of COVID-19 vaccination before pregnancy with birth outcomes and venous thromboembolism as an adverse pregnancy-related outcome, accounting for type of vaccination, number of doses and previous COVID-19 diagnosis, and in sub-groups defined by maternal age, ethnicity, deprivation score, and obstetric or clinical risk.

## Methods

### Data sources

De-identified, individual-level, population-scale, linked electronic health records (EHR) were analysed within the NHSE Secure Data Environment (SDE), made available through the BHF Data Science Centre's CVD-COVID-UK/COVID-IMPACT Consortium. The analysis was conducted according to pre-specified protocols and analysis plans, and published phenotyping and analysis code (available at https://github.com/BHFDSC/CCU036_01). We used the Strengthening the REporting of studies Conducted using Observational Routinely collected Data (RECORD) guideline to write this paper (Supplementary Table S1). The NHSE SDE provides access to primary care data (General Practice Extraction Service Data for Pandemic Planning and Research (GDPPR)) from 96% of general practices (GP) in England, linked to secondary care data including all NHS hospital maternity admissions (Hospital Episode Statistics Admitted Patient Care Maternity data), COVID-19 vaccination data (NHS England Immunisation Management System), COVID-19 laboratory testing data (Second Generation Surveillance System) and death registrations.^15^ Clinical diagnoses were classified using clinician verified Systematized Nomenclature of Medicine Clinical Terms (SNOMED-CT) and ICD-10 (International Classification of Diseases, 10th Revision) code lists for primary and hospital admission data, respectively. Further information on the data used can be found on the Health Data Research UK Gateway. The North East–Newcastle and North Tyneside 2 research ethics committee provided ethical approval for the CVD-COVID-UK/COVID-IMPACT research programme (REC No 20/NE/0161).

### Study population

Our cohort included all women aged between 18 and 45 years registered with a GP in England and alive on 8th December 2020, the official start-date of the COVID-19 vaccination programme in England and the start date for this study.^16^ We included pregnancies with a birth outcome recorded in the hospital maternity dataset with an estimated pregnancy start date between the study start date and 31st December 2021. All pregnancies with a birth outcome recorded by 28th October 2022 (allowing for up to 43 weeks of gestation) were included. We excluded individuals with invalid pseudo-identifiers. Early pregnancy loss is not well captured in EHRs and therefore we excluded deliveries occurring prior to 24 weeks gestation. Deliveries recorded as occurring beyond 43 completed weeks of gestation were also excluded. Estimated pregnancy start dates were calculated as actual delivery date minus number of completed gestational weeks, as recorded at delivery in hospital admission data.

### COVID-19 vaccination

Date of COVID-19 vaccination, and vaccine type (ChAdOx1-S and BNT162b2 hereafter referred to as viral-vector and mRNA vaccine, respectively) were ascertained from the COVID-19 vaccination dataset, which includes all vaccinations administered by the NHS in England. The primary analysis focused on exposure to at least one dose of vaccination before estimated pregnancy start date, compared to starting the pregnancy unvaccinated. Due to the observed length of follow-up, all exposed pregnancies started within 12 months of receiving a vaccine. Pregnancies with the first dose of vaccine after delivery were considered unexposed, whereas those who received a first dose during pregnancy were excluded from the analysis. We also ran secondary analyses investigating the association of alternative exposure classifications.

### Birth and pregnancy-related outcomes

Birth and pregnancy-related outcomes were defined using hospital maternity admission data. Preterm birth was defined as a live birth before 37 completed weeks of gestation (yes, no), further classified into “extremely and very preterm (24–31 wks)” and “moderate to late preterm (32–36 wks)”. We included all preterm births, whether spontaneous or iatrogenic. We classified small-for-gestational age as all infants born with a birthweight in the lowest fifth percentile in the population. Data on stillbirth were derived from hospital admissions and national death registry data. We included venous thromboembolism during pregnancy as a measure of possible adverse effect, derived from hospital records, with codes in any position in hospital data used. The earliest date recorded of the outcome event across all data sources was used.

### Covariates

We used the modified disjunctive cause criterion to select covariates, including causes of vaccination (exposure), causes of the outcomes, and shared causes.^17^ The final list was guided by updated recommendations specific to aetiological studies of COVID-19 vaccines and pregnancy using administrative data.^18^ Primary and secondary care records up to the study start date were used to define socio-demographic and pre-existing health factors. We additionally included a proxy for clinical vulnerability which indexed prioritisation for COVID-19 vaccination, defined as priority group assigned by the Joint Committee on Vaccination and Immunisation (JCVI). In England, the most clinically vulnerable and frontline health and social care workers became eligible to receive their first vaccination from December 8th 2020, whilst all adults became eligible by June 18th 2021.^19^ For more information on covariates, please refer to the Supplementary Materials (Supplementary Table S2).

### Statistical analysis

Follow up time began from 13 weeks of gestation and was censored at the outcome event (for venous thromboembolism during pregnancy), or began at 24 weeks gestation and was censored at delivery (for preterm, small-for gestational age, and stillbirth). We described baseline demographic and clinical characteristics by calculating number of events and person-years of follow-up per outcome by vaccination status, and estimated incidence rates (per 100,000 person-years) and 95% confidence intervals using the exact method.

We fitted Cox-proportional hazards models with gestational age in days as the time scale and the start of follow-up as the origin of the analysis. For each outcome, we estimated hazard ratios (HRs) comparing the incidence of events for women who were administered the first dose of vaccine prior to pregnancy (up to 12 months) and women who started and completed the pregnancy unvaccinated. Using this approach, we estimated HRs for outcome events after exposure. We formally tested for deviations from the proportional hazard assumption for each model, and where violated, took the following steps to reduce bias: a) split the follow-up time into periods when number of events allowed (<32, 32–36 and >36 wks gestation for small-for-gestational age, and first two periods for preterm birth); and b) added an interaction terms with gestational age for any covariate where there was evidence of deviation.

To control for confounding, we estimated three sets of HRs for each model: unadjusted, adjusted for maternal age (continuous, computed in days) and calendar month of start of pregnancy (as a cubic spline, setting the degrees to three and boundary knots at 0 and 12, representing the beginning and end of the calendar year, respectively). We tested the linearity assumption for maternal age by plotting the Martingale residual against the predictor. To account for changes in risk over time, we specified a piecewise polynomial term for calendar month in the model, which excluded any internal knots. Maximally adjusted models additionally included ethnicity, region, level of deprivation, smoking status, parity, prior stillbirth, history of COVID-19 diagnosis, JCVI priority group and comorbidities. For all models and all outcomes, we tested for influential observations by producing index plots for each of the regression coefficients.

#### Subgroup analyses

To investigate the presence of groups with increased vulnerability, we performed subgroup analyses for categories of: maternal age (18–24, 25–29, 30–39, 40–45 years), area-based deprivation index (high, low), ethnicity (white, minority ethnic group, other, unknown), past COVID-19 diagnosis (yes, no), previous pregnancy (yes, no), and multifetal gestation (yes, no). Differences across sub-groups were tested using interaction analyses on a multiplicative scale.

#### Secondary and sensitivity analyses

We conducted secondary analyses by refining the exposure definition according to number of doses and vaccine type. We included an ‘unknown’ category for missing covariates (smoking and ethnicity), and imputed missing gestational age at birth with known median gestational age (40 weeks). We ran sensitivity analyses to check to what extent results were robust to: 1) imputation of gestational age (restricting to pregnancies with known gestational age), 2) incident SARS-CoV-2 infections during pregnancy (censoring at positive test results), and 3) patients' clinical vulnerability and timing of vaccination (stratifying analysis by JCVI priority group in a deviation from our protocol). For more details on secondary and sensitivity analyses, derivation of absolute excess risks and other analytical specifications, please refer to the Supplementary Materials.

#### Software

Data manipulation and analyses used SQL and Python in Databricks and RStudio (Professional) Version 1.3.1093.1 driven by R Version 4.0.5.

#### Role of the funding source

The funding source supported the development of the technical and governance infrastructure to facilitate data access for research and the analysts time to conduct the study. The funders played no role in study design and protocol development, data curation and analysis, interpretation or writing the manuscript.

## Results

During the study period there were 288,150 pregnancies recorded, of which 284,275 were among eligible women aged 18–45 years old. Of these women, 97,285 received their first COVID-19 vaccination dose during their pregnancy and were excluded (Supplementary Figure S1).

Of the 186,990 eligible pregnancies, excluding venous thromboembolism within 12 weeks of gestation, there were 186,925 pregnancies in the analysis for venous thromboembolism (186,470 pregnancies for the birth outcomes). 53,295 (28.5%) women had received their first COVID-19 vaccination dose prior to the start of the pregnancy (Table 1), of which 26,395 (49.5%) received at least one more dose during pregnancy, and 4640 were not further vaccinated in pregnancy. Median time between first vaccination and estimated pregnancy start date was 51 days (interquartile range [IQR]: 21–92 days). Women were followed-up for the following median times in days: 91 (IQR: 91–91) for preterm birth, 105 (IQR: 98–112) for small-for-gestational age and stillbirth, 189 (IQR: 182–196) for venous thromboembolism. Gestational age was recorded for 145,695 (78%) of pregnancies. At the estimated pregnancy start date, women were on average 29.4 years old (SD 5.3 years), mostly of white ethnicity (n = 135,885, 72.7%) and residing in areas with high levels of deprivation (n = 53,540, 28.6%; see Table 1). Differences between the vaccinated and unvaccinated groups reflect predictors of COVID-19 vaccine uptake. The unvaccinated group, compared to the vaccinated group, were more likely to be aged under 30 (56.6 %vs. 36.6%), from minority ethnic groups (24.5% vs. 16.4%) and the most deprived background (32.1% vs. 20%). A higher proportion of those vaccinated were in the most vulnerable JCVI group (3.1% vs. 0.9%) and had pre-existing conditions, including diabetes mellitus (10.4% vs. 4.6%) and chronic kidney disease (1.2% vs. 0.8%), and were dispensed with medication (for example 1.5% (n = 790) of those vaccinated were dispensed with blood pressure lowering drugs compared to 0.6% (n = 800) of the unvaccinated). Comparing different vaccine types, a higher proportion of women who received the viral-vector vaccine were in the most vulnerable JCVI group^4^ (4.0% vs. 2.4%), had pre-existing conditions, including diabetes (13.7% vs. 7.7%) and a prior venous thrombotic event (1.0% vs. 0.5%), and were on blood pressure lowering medication (1.8% vs. 1.2%), compared to those who received the mRNA vaccine (Supplementary Table S3).

**Table 1:** Characteristics of study population (pre-pregnancy).

Characteristic	Preterm, small-for-gestational age, & stillbirth			Venous event		
	Vaccinated before pregnancy (n = 53,060)	Not vaccinated before pregnancy (n = 133,405)	Overall (n = 186,470)	Vaccinated before pregnancy (n = 53,295)	Not vaccinated before pregnancy (n = 133,630)	Overall (n = 186,925)
**Age (years)**^a^	31.1 (5.1)	28.7 (5.4)	29.4 (5.4)	31.1 (5.1)	28.7 (5.4)	29.4 (5.4)
**Age group (years)**						
18–24	5950 (11.2%)	31,460 (23.6%)	37,410 (20.1%)	5985 (11.2%)	31,510 (23.6%)	37,495 (20.1%)
25–29	13,325 (25.1%)	44,090 (33.0%)	57,415 (30.8%)	13,380 (25.1%)	44,150 (33.0%)	57,530 (30.8%)
30–34	20,025 (37.7%)	37,860 (28.4%)	57,885 (31.0%)	20,100 (37.7%)	37,940 (28.4%)	58,040 (31.0%)
35–39	11,210 (21.1%)	16,775 (12.6%)	27,985 (15.0%)	11,265 (21.1%)	16,800 (12.6%)	28,065 (15.0%)
40–45	2555 (4.8%)	3220 (2.4%)	5775 (3.1%)	2570 (4.8%)	3225 (2.4%)	5795 (3.1%)
**Ethnicity**						
White	42,580 (80.3%)	92,955 (69.7%)	135,535 (72.7%)	42,765 (80.2%)	93,120 (69.7%)	135,885 (72.7%)
Asian or Asian British	6315 (11.9%)	18,530 (13.9%)	24,850 (13.3%)	6335 (11.9%)	18,555 (13.9%)	24,890 (13.3%)
Black or Black British	1445 (2.7%)	10,170 (7.6%)	11,615 (6.2%)	1460 (2.7%)	10,190 (7.6%)	11,650 (6.2%)
Mixed	950 (1.8%)	4025 (3.0%)	4975 (2.7%)	960 (1.8%)	4030 (3.0%)	4990 (2.7%)
Other	1675 (3.2%)	7085 (5.3%)	8760 (4.7%)	1680 (3.2%)	7095 (5.3%)	8775 (4.7%)
Unknown	95 (0.2%)	645 (0.5%)	735 (0.4%)	95 (0.2%)	645 (0.5%)	740 (0.4%)
**Region**						
East midlands	4685 (8.8%)	10,835 (8.1%)	15,520 (8.3%)	4695 (8.8%)	10,845 (8.1%)	15,540 (8.3%)
East of England	6290 (11.9%)	14,870 (11.1%)	21,160 (11.3%)	6310 (11.8%)	14,885 (11.1%)	21,195 (11.3%)
London	7580 (14.3%)	26,220 (19.7%)	33,800 (18.1%)	7595 (14.2%)	26,240 (19.6%)	33,835 (18.1%)
North East	2800 (5.3%)	6135 (4.6%)	8935 (4.8%)	2805 (5.3%)	6135 (4.6%)	8940 (4.8%)
North West	6805 (12.8%)	18,255 (13.7%)	25,060 (13.4%)	6820 (12.8%)	18,280 (13.7%)	25,100 (13.4%)
South East	8825 (16.6%)	18,340 (13.7%)	27,165 (14.6%)	8925 (16.7%)	18,395 (13.8%)	27,325 (14.6%)
South West	5420 (10.2%)	9385 (7.0%)	14,805 (7.9%)	5445 (10.2%)	9405 (7.0%)	14,850 (7.9%)
West Midlands	5380 (10.1%)	16,755 (12.6%)	22,135 (11.9%)	5400 (10.1%)	16,795 (12.6%)	22,195 (11.9%)
Yorkshire and The Humber	5270 (9.9%)	12,620 (9.5%)	17,890 (9.6%)	5300 (9.9%)	12,650 (9.5%)	17,950 (9.6%)
**Deprivation index**						
1–2 (Most deprived)	10,605 (20.0%)	42,795 (32.1%)	53,400 (28.6%)	10,660 (20.0%)	42,875 (32.1%)	53,540 (28.6%)
3–4	10,800 (20.4%)	32,775 (24.6%)	43,575 (23.4%)	10,860 (20.4%)	32,830 (24.6%)	43,685 (23.4%)
5–6	10,830 (20.4%)	24,495 (18.4%)	35,325 (18.9%)	10,880 (20.4%)	24,530 (18.4%)	35,410 (18.9%)
7–8	10,685 (20.1%)	19,180 (14.4%)	29,865 (16.0%)	10,720 (20.1%)	19,215 (14.4%)	29,935 (16.0%)
9–10 (Least deprived)	10,140 (19.1%)	14,160 (10.6%)	24,300 (13.0%)	10,175 (19.1%)	14,180 (10.6%)	24,355 (13.0%)
**Parity**						
Multiparous	35,810 (67.5%)	86,300 (64.7%)	122,110 (65.5%)	35,990 (67.5%)	86,450 (64.7%)	122,440 (65.5%)
Nulliparous	17,255 (32.5%)	47,105 (35.3%)	64,360 (34.5%)	17,305 (32.5%)	47,180 (35.3%)	64,485 (34.5%)
**Reproductive history**						
Stillbirth	345 (0.6%)	895 (0.7%)	1240 (0.7%)	345 (0.6%)	900 (0.7%)	1245 (0.7%)
**JCVI group**						
Group 4 (vulnerable)	1665 (3.1%)	1185 (0.9%)	2850 (1.5%)	1670 (3.1%)	1190 (0.9%)	2855 (1.5%)
Group 10 (age ≥40)	2675 (5.0%)	3775 (2.8%)	6450 (3.5%)	2690 (5.0%)	3780 (2.8%)	6470 (3.5%)
Group 11 (age ≥30)	30,785 (58.0%)	56,735 (42.5%)	87,520 (46.9%)	30,910 (58.0%)	56,840 (42.5%)	87,750 (46.9%)
Group 12 (age ≥18)	17,935 (33.8%)	71,710 (53.8%)	89,645 (48.1%)	18,030 (33.8%)	71,820 (53.7%)	89,850 (48.1%)
**Medical history**						
Prior SARS-CoV-2 infection	4210 (7.9%)	7655 (5.7%)	11,865 (6.4%)	4235 (7.9%)	7670 (5.7%)	11,900 (6.4%)
Depression/anxiety	14,915 (28.1%)	31,090 (23.3%)	46,005 (24.7%)	14,990 (28.1%)	31,160 (23.3%)	46,145 (24.7%)
Obesity	8145 (15.4%)	13,740 (10.3%)	21,885 (11.7%)	8185 (15.4%)	13,760 (10.3%)	21,945 (11.7%)
Hypertensive disorder	7375 (13.9%)	13,930 (10.4%)	21,305 (11.4%)	7415 (13.9%)	13,955 (10.4%)	21,365 (11.4%)
Diabetes mellitus	5520 (10.4%)	6185 (4.6%)	11,705 (6.3%)	5540 (10.4%)	6200 (4.6%)	11,735 (6.3%)
Chronic kidney disease	660 (1.2%)	1040 (0.8%)	1705 (0.9%)	665 (1.2%)	1045 (0.8%)	1710 (0.9%)
PCOS	1870 (3.5%)	3075 (2.3%)	4945 (2.7%)	1880 (3.5%)	3085 (2.3%)	4960 (2.7%)
DVT/Pulmonary embolism	385 (0.7%)	575 (0.4%)	960 (0.5%)	380 (0.7%)	570 (0.4%)	950 (0.5%)
Thrombophilia	155 (0.3%)	200 (0.1%)	355 (0.2%)	155 (0.3%)	200 (0.1%)	355 (0.2%)
Venous thrombotic event	385 (0.7%)	575 (0.4%)	960 (0.5%)	380 (0.7%)	570 (0.4%)	950 (0.5%)
**Treatment history**						
Surgery in previous year	7380 (13.9%)	19,150 (14.4%)	26,530 (14.2%)	7415 (13.9%)	19,195 (14.4%)	26,610 (14.2%)
Combined oral contraceptive	2670 (5.0%)	6185 (4.6%)	8855 (4.7%)	2675 (5.0%)	6190 (4.6%)	8865 (4.7%)
BP Lowering drugs	790 (1.5%)	800 (0.6%)	1585 (0.9%)	790 (1.5%)	800 (0.6%)	1590 (0.9%)
Lipid lowering drugs	215 (0.4%)	150 (0.1%)	365 (0.2%)	220 (0.4%)	145 (0.1%)	365 (0.2%)
Immunosuppressants	205 (0.4%)	65 (0.1%)	270 (0.1%)	205 (0.4%)	70 (0.1%)	275 (0.1%)
Anticoagulant	130 (0.2%)	105 (0.1%)	240 (0.1%)	130 (0.2%)	105 (0.1%)	230 (0.1%)
**Smoking status**						
Current	7880 (14.8%)	28,805 (21.6%)	36,685 (19.7%)	7915 (14.9%)	28,860 (21.6%)	36,780 (19.7%)
Never	35,785 (67.4%)	73,655 (55.2%)	109,440 (58.7%)	35,935 (67.4%)	73,760 (55.2%)	109,695 (58.7%)
Ex-smoker	8340 (15.7%)	17,485 (13.1%)	25,820 (13.8%)	8370 (15.7%)	17,515 (13.1%)	25,885 (13.8%)
Missing	1060 (2.0%)	13,460 (10.1%)	14,520 (7.8%)	1075 (2.0%)	13,490 (10.1%)	14,565 (7.8%)

JCVI, Joint Committee for Vaccination and Immunisation; COVID-19, Corona Virus Disease 2019; PCOS, Polycystic ovary syndrome; DVT, Deep Vein Thrombosis; BP, Blood Pressure.

For disclosure control, any cell number <10 is not reported exactly, but as <10, and any number ≥10 has been rounded up to the nearest multiple of 5.

COVID-19 vaccination and birth outcomes: an England-wide cohort study of 186,990 women.

Women with a recorded pregnancy in England with estimated start date between 08 December 2020 and 31 December 2021.

a Mean (SD) reported for continuous variable.

The number, person-years and incidence rates per 100,000 person-years for each pregnancy outcome event are presented by vaccination exposure group in Table 2. There were 12,850 preterm births, 7235 babies born small-for-gestational age, 485 stillbirths, and 300 maternal venous thromboembolism during pregnancy. Amongst women vaccinated, the majority had received a second dose before pregnancy, with similar numbers of women receiving viral-vector and mRNA vaccination. The unadjusted incidence of preterm birth was higher in the vaccinated group, with the highest incidence rates amongst women who received three doses of the vaccine by 24 weeks gestation. Incidence rates for small-for-gestational age were lower in the vaccinated, compared to unvaccinated group. Whilst the incidence of venous thromboembolism during pregnancy was comparable in the unvaccinated and primary vaccinated group, higher incidence rates were found in specific secondary analysis subgroups, for example amongst women who received the viral-vector vaccine or three doses by 24 weeks gestation. The incidence of stillbirth was similar across exposures groups.

**Table 2:** Number of pregnancies and outcome events, person-years of follow-up, and unadjusted incidence rate per 100,000 person-years, by exposure group, for pregnancies in England with estimated start date between 08 December 2020 and 31 December 2021 (primary and secondary analyses).

Exposure group	Preterm				Small-for-gestational age			Stillbirth			Venous events			
	Person-years	No. of pregnancies	Events	Incidence rate (95% CI)	Person-years	Events	Incidence rate (95% CI)	Person-years	Events	Incidence rate (95% CI)	Person-years	No. of pregnancies	Events	Incidence rate (95% CI)
Unvaccinated (*unexposed*)	33,409	133,405	8435	25,248 (24,712, 25,792)	39,437	5510	13,976 (13,609, 14,350)	38,736	375	966 (870, 1069)	69,405	133,630	195	280 (242, 322)
At least one dose (By vaccine type)														
**First dose before pregnancy—Primary analysis**	**13,211**	**53,060**	**4415**	**33,431 (32,453, 34,432)**	**15,405**	**1725**	**11,212 (10,689, 11,753)**	**15,130**	**115**	**747 (616, 898)**	**27,333**	**53,295**	**105**	**388 (318, 469)**
Of which: viral-vector vaccine (ChAdOx1)	6107	24,490	2005	32,804 (31,383, 34,273)	7110	795	11,168 (10,405, 11,973)	6983	65	902 (693, 1154)	12,604	24,545	65	516 (398, 657)
Of which: mRNA vaccine (BNT162b2)	6905	27,760	2325	33,662 (32,307, 35,059)	8065	905	11,211 (10,492, 11,966)	7921	50	619 (458, 818)	14,312	27,935	40	286 (206, 389)
At least 2 doses (By timing of 2nd dose)														
Two doses before pregnancy	10,944	43,975	3695	33,785 (32,704, 34,891)	12,771	1410	11,026 (10,458, 11,618)	12,543	100	797 (649, 970)	22,655	44,185	90	388 (312, 479)
Three doses by 24 weeks gestation	1155	4815	805	69,723 (64,989, 74,711)	1322	130	9986 (8355, 11,842)	4815	15	1001 (533, 1712)	2411	4900	10	498 (257, 870)
Documented gestational age														
Unvaccinated (*unexposed*)	24,980	100,040	7425	29,720 (29,047, 30,404)	29,116	5510	18,930 (18,434, 19,437)	28,593	205	714 (619, 818)	51,592	100,250	140	275 (232, 324)
First dose before pregnancy	10,082	40,620	3820	37,892 (36,700, 39,114)	11,589	1725	14,903 (14,209, 15,623)	11,380	60	527 (402, 679)	20,727	40,845	80	376 (297, 470)

CI, confidence interval; # number of sensitivity analysis.

For disclosure control, any cell number <10 is not reported exactly, but as <10, and any number ≥10 has been rounded up to the nearest multiple of 5.

**Fig. 1:**
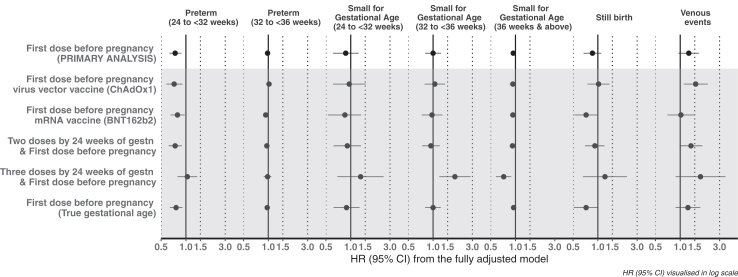
Adjusted hazard ratios (aHR) of pregnancy outcomes following pre-pregnancy vaccination exposure among pregnancies in England with estimated start date between 08 December 2020 and 31 December 2021 (primary and secondary analyses).

The maximally adjusted HR (aHR) for extreme and very preterm birth and moderate to late preterm were 0.74 (95% CI 0.63, 0.88) and 0.98 (95% CI 0.93, 1.03), respectively in pregnancies among women who received first dose of COVID-19 vaccination prior to becoming pregnant, compared to those who were not vaccinated (Fig. 1; Supplementary Table S4). Similar aHRs of preterm birth were observed across secondary analyses, except for women who received three doses by 24 weeks gestation, for whom there was no association between COVID-19 vaccination pre-pregnancy and extremely and very preterm birth.

The aHR comparing the incidence of term (>36 wks) small-for-gestational age after COVID-19 vaccination compared to without was 0.94 (0.88, 1.00). Findings were comparable across secondary analyses, with the exception for the aHRs comparing women with three doses by 24 weeks gestation to unvaccinated women, which were in opposite direction for small-for-gestational age between 32 and 36 wks (1.84 [2.19, 1.85]), and after 36 wks (0.72 [0.58, 0.89]).

In maximally adjusted models, the aHR for stillbirth in the vaccinated compared to unvaccinated group was 0.86 [0.67, 1.10]. Results were similarly uncertain in secondary analyses, and more extreme amongst women who received the mRNA vaccine, where the aHR was 0.72 (0.52, 1.00) compared to those who were unvaccinated, and when restricting to the cohort with complete gestational age (aHR 0.72 [0.51, 1.01]).

The aHR for venous thromboembolism during pregnancy was 1.27 (0.95, 1.69), compared to those unvaccinated. Higher incidence was reported amongst women who received the viral-vector vaccine (aHR 1.54 [1.10, 2.16]), especially amongst those in JCVI group 11 (i.e. not clinically vulnerable women aged 30–39) (1.88 [1.14, 3.11]), those who received two doses before pregnancy (aHR 1.35 [0.98, 1.85]), or three doses by 24 weeks gestations (1.76 [0.88, 3.55]), compared to unvaccinated.

We found no deviations from linearity for the covariate maternal age (in days) (Supplementary Figure S5), or deviations from the proportional hazard assumptions (Supplementary Table S7 and Supplementary Figures S4 and S6), and influential observations plots showed no concerning patterns (Supplementary Figure S7).

**Fig. 2:**
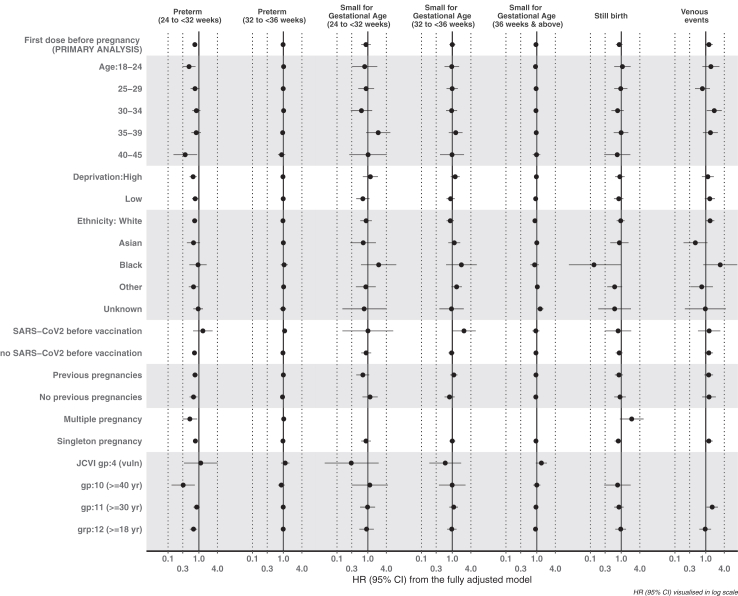
Adjusted hazard ratios (aHR) of pregnancy outcomes following pre-pregnancy vaccination exposure among pregnancies in England with estimated start date between 08 December 2020 and 31 December 2021, stratified by maternal characteristics.

Findings from models stratified by sociodemographic, reproductive, or COVID-19 history factors were generally consistent with the main analysis (Fig. 2; Supplementary Table S5). Isolated exceptions are listed as follows. For preterm birth, vaccinated women in the older age groups had lower aHRs compared with those unvaccinated (p-value_interaction_=<0.0001). Incidence of small-for-gestational age was lower amongst vaccinated white women, compared to those unvaccinated, but similar amongst women from minority ethnic groups or those missing ethnicity (p-value_interaction_=<0.0001). There was a higher aHR for stillbirth in multiple compared to singleton pregnancies (p-value_interaction_=<0.0001).

Despite modest differences in the magnitude of aHRs for venous thromboembolism in subgroups defined by age, ethnicity and deprivation, results were consistent with the main analyses.

Receiving a first dose of COVID-19 vaccine before pregnancy, compared to starting the pregnancy unvaccinated, prevented an estimated 452 preterm birth and 391 small-for-gestational age babies per 100,000 vaccinations prior to pregnancy, regardless of the type of vaccine. However, women who received the viral-vector vaccine before their pregnancy experienced an estimated excess risk of 107 venous thromboembolism per 100,000 vaccinations prior to pregnancy, compared to those who remained unvaccinated.

Results were generally comparable in analyses 1) restricting to pregnancies with known gestational age (Fig. 1, Supplementary Table S4); 2) censored after a COVID-19 diagnosis during pregnancy (Supplementary Figure S2, Supplementary Table S6); and 3) stratified by JCVI priority group (Fig. 2, Supplementary Table S5). However, there was no evidence of an association between viral-vector vaccine and venous thromboembolism during pregnancy when women were censored after a COVID-19 diagnosis in pregnancy.

## Discussion

Based on this population-wide cohort study of nearly 190,000 pregnancies reaching 24 weeks gestation, we found evidence that vaccination up to 12 months before pregnancy was associated with lower risks of extremely and very preterm birth and small-for-gestational age babies for any vaccine type, and lower risks of stillbirth limited to those vaccinated with mRNA vaccine, compared to unvaccinated women. Results were generally consistent for different dose regimens. Higher incidence of maternal venous thromboembolism during pregnancy was observed amongst women who received the viral-vector, but not the mRNA vaccine, compared to unvaccinated women. After accounting for measured confounding factors, the increased risk was estimated to be as low as 10% and as large as a doubling of the risk in the unvaccinated. These results—based on over 50,000 pregnancies pre-conceptionally exposed to at least one vaccine dose—did not differ when stratified by maternal socio-demographic, reproductive and COVID-19 history factors, or in further analyses accounting for missing gestational age, the impact of SARS-CoV-2 infection in pregnancy, or the changing composition over time of the vaccinated vs. unvaccinated groups as the vaccination program was rolled-out. Our findings are novel, as there is no comprehensive and large-scale evidence on the association of COVID-19 vaccination with mRNA and viral-vector vaccines before pregnancy and birth outcomes.

This is by far the largest study to investigate the association of COVID-19 vaccination before pregnancy with subsequent birth outcomes. Directly comparable figures from the literature are limited to four small studies from the Netherlands and China, including totals of 1700 and 4000 women vaccinated pre-pregnancy with mRNA and inactivated COVID-19 vaccines, respectively. These found no evidence of increased risk of adverse fetal outcomes, and indicate a reduced risk of preterm births for women receiving at least two doses before or in the very early stages of pregnancy.^20^, ^21^, ^22^, ^23^

A larger body of evidence exists about receiving COVID-19 vaccination during pregnancy, generally showing no increased risk or reduced risk of both preterm birth and stillbirth, and no increased risk of other adverse pregnancy outcomes. These results come from large population-based studies using similarly robust methodologies including those that minimise immortal time bias, almost identical confounding adjustments and similar if not overlapping population samples. Based on the largest systematic review available, including over 117,000 women vaccinated predominantly with mRNA vaccines during pregnancy, the risk of stillbirth was lower by 15% (pooled OR 0.85, 95% CI 0.73, 0.99) in the vaccinated cohort.^4^ A large Canadian study observed a reduction in risk of very preterm birth (aHR 0.80 [0.67, 0.95]) and stillbirth (aHR 0.64 [0.51, 0.84]), but not preterm birth overall or small-for-gestational age, based on 43,000 women vaccinated during pregnancy.^18^

In contrast to our results for vaccination exposures, COVID-19 diagnosis during pregnancy (in unvaccinated women) has been associated with increased risks of preterm birth, stillbirth and admission to neonatal intensive care units.^24^ Pregnancies which are protected by vaccination from the outset are less likely to experience the severe consequences of COVID-19.^8^ Our study demonstrates that it is plausible that this protection extends to birth and perinatal outcomes. This aligns with recent evidence that maternal immunisation with COVID-19 vaccine before pregnancy is transferred to the newborn via the placenta, with antibody levels increasing with number of vaccine doses.^22^

We found some evidence of an increase in venous thromboembolism incidence during pregnancy for women receiving one or more viral-vector vaccines before pregnancy, with (age-adjusted) incidence rates of 475 per 100,000 person-years in the viral-vector vaccine-vaccinated group, compared to 320 and 284 per 100,000 person-years in the mRNA-vaccinated and unvaccinated groups, respectively (Supplementary Figure S3). In absolute terms, this corresponds to an excess risk of 127 venous thromboembolism during pregnancy per 100,000 vaccinations prior to pregnancy. This increased risk is compatible with that previously published for 18–63 year olds in the general population following the same vaccine. While it is possible that some residual confounding might inflate the current estimate, especially if clinically vulnerable women were more likely to receive the viral-vector vaccine (compared to mRNA, as reflected in the distributions of known confounders across recipients of the two vaccines), we cannot at present rule out the possibility that this might be a true causal effect. Unfortunately, the long interval between first dose and venous thromboembolism events in pregnancy prevents us from investigating the expected time-waning of vaccine side effects, which is well established in the case of vaccine-induced immune thrombotic thrombocytopenia over shorter time frames. Women of reproductive age are known to experience a higher baseline risk of venous thromboembolism compared to men,^25^ as well as higher absolute risks attributable to viral-vector vaccination for a specific type of venous thromboembolism (intracranial venous thrombosis).^26^ However, previous studies did not investigate whether the association was indeed due or confined to pregnancy. Other studies specifically examining the safety of COVID-19 vaccine types during pregnancy in UK populations could not estimate excess (pregnancy) venous thromboembolism risks attributable to viral-vector vaccine due to insufficient numbers receiving this vaccine.^27^ Therefore, ours remains the only available estimate of the excess risk of developing venous thromboembolism in pregnancy after receiving viral-vector vaccine vaccination before pregnancy. For context, our population seems to experience higher baseline risk of pregnancy-related venous thromboembolism compared to estimates published for other high-income countries, with age-adjusted incidence rates of over 280 compared to approximately 100 events per 100,000 women-years, respectively^28^^,^^29^ (Supplementary Figure S3). Importantly, the relative risk for venous thromboembolism associated with the viral-vector vaccine before pregnancy is considerably lower than that conferred by SARS-CoV-2 infection (2.5 folds),^30^ which it prevents, and also to the risk induced by widely used and generally deemed safe medications such as hormone replacement therapy and oral contraceptives (two-to-six folds).^31^

The study is the first to use large, population-wide electronic health resource, from a publicly funded healthcare system and therefore has nearly complete coverage of all births beyond 24 weeks and vaccinations in the English population, minimizing the risk of selection bias or misclassification. Moreover, our results are unlikely to suffer from collider bias (a type of selection bias) that could be induced through ascertaining pregnancies based on hospital birth records, since there is no evidence that either female fertility or miscarriage risk are affected by pre-conception COVID-19 vaccination.^32^^,^^33^

Healthy vaccine bias has been reported in observational studies of vaccination, including for vaccination during pregnancy.^34^ We extensively adjusted for most of the confounders recommended to minimise the risk of confounding in COVID-19 vaccine safety studies of pregnancy and birth outcomes,^18^ except prenatal care adherence and occupational status (e.g. whether a frontline healthcare worker). However, for the latter we were able to adjust for JCVI priority group, proxying for a combination of clinical vulnerability and at-risk frontline healthcare work. The latter were mainly women who received two doses of viral-vector vaccine, for whom we found no difference in association of vaccination status with birth outcomes compared to those receiving the mRNA vaccine (both protective associations). This indicates that the residual confounding due to not explicitly adjusting for occupational exposure in these analyses is likely negligible. On the other hand, we observe an association with venous thromboembolism in the opposite direction for viral-vector vaccine but not mRNA vaccines, with the former offered to more clinically vulnerable groups. This finding was confirmed in analyses limited to non-clinically vulnerable individuals vaccinated during the universal vaccine roll-out (not highly exposed healthcare workers), which again rules out a substantial role of residual confounding. However, we cannot rule out the presence of residual confounding due to measurement error in confounders such as smoking, and of unmeasured confounding due to factors that we had not identified a priori. When comparing baseline characteristics across vaccine groups, those who received viral-vector vaccine were more likely to be in the most vulnerable JCVI group, have existing medical conditions and be on medications, compared to those who received mRNA. We are also limited in the causal interpretation of the results due to the built-in selection bias in HR estimates.^35^ Another limitation of our study is the amount of missing data for gestational age. If those with missing gestational age were more likely to both receive pre-pregnancy vaccination and to experience adverse pregnancy outcomes (e.g. preterm labour), our main analysis assuming term births would be biased. However, results were unchanged when restricting to pregnancies with recorded gestational age, suggesting that the protection afforded by vaccines prior to the start of the pregnancy is unlikely to be explained by the poor data quality or missing data patterns.

There are three additional limitations worth mentioning. The first is the exclusion of home births (2% of all births in England^36^), due to identifying deliveries from hospital records, which could lead to lack of generalisability to this (already very low risk) group of women. The second is performing interaction tests on the multiplicative rather than additive scale, to minimise computational burden in the absence of consistent differences in the sub-group analyses, which could miss subtle heterogeneity across groups. The third is combining spontaneous and iatrogenic preterm births into an overall preterm birth phenotype, despite them being clearly shown to be characterised by different aetiologies and therefore requiring cause-specific interventions.^37^

We found evidence that vaccination against COVID-19 up to 12 months before pregnancy was generally associated with lower risks of adverse birth outcomes amongst those that reached at least 24 weeks gestation, overall or in selected subgroups of the general population. Receiving a viral-vector vaccine before pregnancy, but not the mRNA vaccine, was found to be associated with a higher risk of venous thromboembolism in pregnancy, although considerably smaller in magnitude than that attributable to SARS-CoV-2 infection in pregnancy or to some hormone replacement therapies and oral contraceptives. Therefore, our results support and strengthen the recommendations to vaccinate all women of childbearing potential, especially those intending to become pregnant within 12 months, but if possible to offer them an mRNA vaccine over viral vector vaccines.

## Contributors

LZ, RD, ER, CB, KB, CT, VW and AMW contributed to Conceptualization. LZ, RD, ER, VW and AMW contributed to Methodology. AKS, RD and MR contributed to Formal analysis. LZ and RD contributed to Investigation. AKS, RD, MR and ER contributed to Data Curation. RD, AKS and LZ contributed to Writing–Original Draft. All authors contributed to Writing–Review & Editing. AKS contributed to Visualisation. RD and LZ contributed to Supervision. AKS and RD had access to and verified the data. RD and LZ were responsible for the decision to submit the manuscript.

## Data sharing statement

The data used in this study are available in NHS England’s Secure Data Environment (SDE) service for England, but as restrictions apply they are not publicly available (https://digital.nhs.uk/services/secure-data-environment-service). The CVD-COVID-UK/COVID-IMPACT programme, led by the BHF Data Science Centre (https://bhfdatasciencecentre.org/), received approval to access data in NHS England’s SDE service for England from the Independent Group Advising on the Release of Data (IGARD) (https://digital.nhs.uk/about-nhs-digital/corporate-information-and-documents/independent-group-advising-on-the-release-of-data) via an application made in the Data Access Request Service (DARS) Online system (ref. DARS-NIC-381078-Y9C5K) (https://digital.nhs.uk/services/data-access-request-service-dars/dars-products-and-services). The CVD-COVID-UK/COVID-IMPACT Approvals & Oversight Board (https://bhfdatasciencecentre.org/areas/cvd-covid-uk-covid-impact/) subsequently granted approval to this project to access the data within NHS England’s SDE service for England. The de-identified data used in this study were made available to accredited researchers only. Those wishing to gain access to the data should contact bhfdsc@hdruk.ac.uk in the first instance. The study protocol, which includes the statistical analysis plan, phenotyping and analytical code is available in a repositoryin GitHub here: https://github.com/BHFDSC/CCU036_01.

## Declaration of interests

CT has received funding paid to University College London from GlaxoSmithKline (GSK), outside the scope of the submitted work. The other Authors declare no conflict of interest.

## References

[1] Allotey J., Chatterjee S., Kew T. (2022). SARS-CoV-2 positivity in offspring and timing of mother-to-child transmission: living systematic review and meta-analysis. BMJ.

[2] World Health Organisation COVID-19 vaccines. https://www.who.int/emergencies/diseases/novel-coronavirus-2019/covid-19-vaccines.

[3] Health Security Agency COVID-19 vaccination: a guide on pregnancy and breastfeeding. HM Government. https://www.gov.uk/government/publications/covid-19-vaccination-women-of-childbearing-age-currently-pregnant-planning-a-pregnancy-or-breastfeeding/covid-19-vaccination-a-guide-on-pregnancy-and-breastfeeding.

[4] Prasad S., Kalafat E., Blakeway H. (2022). Systematic review and meta-analysis of the effectiveness and perinatal outcomes of COVID-19 vaccination in pregnancy. Nat Commun.

[5] Villar J., Soto Conti C.P., Gunier R.B. (2023). Pregnancy outcomes and vaccine effectiveness during the period of omicron as the variant of concern, INTERCOVID-2022: a multinational, observational study. Lancet.

[6] Barros F.C., Gunier R.B., Rego A. (2024). Maternal vaccination against COVID-19 and neonatal outcomes during Omicron: INTERCOVID-2022 study. Am J Obstet Gynecol.

[7] Rimmer M.P., Teh J.J., Mackenzie S.C., Al Wattar B.H. (2023). The risk of miscarriage following COVID-19 vaccination: a systematic review and meta-analysis. Hum Reprod.

[8] Bosworth M.L., Schofield R., Ayoubkhani D. (2023). Vaccine effectiveness for prevention of covid-19 related hospital admission during pregnancy in England during the alpha and delta variant dominant periods of the SARS-CoV-2 pandemic: population based cohort study. BMJ Med.

[9] NHS England (2021).

[10] Royal College of Midwives, Royal College of Obstetricians & Gynaecologists RCOG COVID-19 vaccination guidance timeline. https://www.rcog.org.uk/media/k5aniwh1/rcog-covid-19-vaccination-guidance-timeline.pdf.

[11] Grunebaum A., Chervenak F.A. (2023). Physician hesitancy to recommend COVID-19 vaccination in pregnancy as a cause of maternal deaths - robert Brent was prescient. Birth Defects Res.

[12] Skirrow H., Barnett S., Bell S. (2022). Women's views on accepting COVID-19 vaccination during and after pregnancy, and for their babies: a multi-methods study in the UK. BMC Pregnancy Childbirth.

[13] Magee L.A., Molteni E., Bowyer V. (2023). National surveillance data analysis of COVID-19 vaccine uptake in England by women of reproductive age. Nat Commun.

[14] European Medicines Agency (2021). AstraZeneca’s COVID-19 vaccine: EMA finds possible link to very rare cases of unusual blood clots with low blood platelets. Sci Med Health. https://www.ema.europa.eu/en/news/astrazenecas-covid-19-vaccine-ema-finds-possible-link-very-rare-cases-unusual-blood-clots-low-blood-platelets.

[15] Wood A., Denholm R., Hollings S. (2021). Linked electronic health records for research on a nationwide cohort of more than 54 million people in England: data resource. BMJ.

[16] Landmark moment as first NHS patient receives COVID-19 vaccination. NHS England. https://www.england.nhs.uk/2020/12/landmark-moment-as-first-nhs-patient-receives-covid-19-vaccination/.

[17] VanderWeele T.J. (2019). Principles of confounder selection. Eur J Epidemiol.

[18] Fell D.B., Dimitris M.C., Hutcheon J.A. (2021). Guidance for design and analysis of observational studies of fetal and newborn outcomes following COVID-19 vaccination during pregnancy. Vaccine.

[19] Baraniuk C. (2021). Covid-19: how the UK vaccine rollout delivered success, so far. BMJ.

[20] Zhao Y., Zhao Y., Su X. (2023). No association of vaccination with inactivated COVID-19 vaccines before conception with pregnancy complications and adverse birth outcomes: a cohort study of 5457 Chinese pregnant women. J Med Virol.

[21] De Feijter M., Vissers L.C.M., Davidson L., Kant A.C., Woestenberg P.J. (2023). The risk of preterm labor after COVID-19 vaccination before and during pregnancy. Front Drug Saf Regul.

[22] Yang Y., Xing H., Zhao Y. (2023). Transplacental transmission of SARS-CoV-2 immunoglobulin G antibody to infants from maternal COVID-19 vaccine immunization before pregnancy. J Med Virol.

[23] Chen Z., Mu X., Wang X. (2023). Association of maternal inactivated COVID-19 vaccination within 3 Months before conception with neonatal outcomes. Vaccines.

[24] Allotey J., Fernandez S., Bonet M. (2020). Clinical manifestations, risk factors, and maternal and perinatal outcomes of coronavirus disease 2019 in pregnancy: living systematic review and meta-analysis. BMJ.

[25] Heit J.A. (2015). Epidemiology of venous thromboembolism. Nat Rev Cardiol.

[26] Whiteley W.N., Ip S., Cooper J.A. (2022). Association of COVID-19 vaccines ChAdOx1 and BNT162b2 with major venous, arterial, or thrombocytopenic events: a population-based cohort study of 46 million adults in England. PLoS Med.

[27] Mensah A.A., Stowe J., Jardine J.E. (2023). Covid-19 vaccine safety in pregnancy, a nested case-control study in births from April 2021 to March 2022, England. Epidemiology.

[28] Heit J.A., Kobbervig C.E., James A.H., Petterson T.M., Bailey K.R., Melton L.J. (2005). Trends in the incidence of venous thromboembolism during pregnancy or postpartum: a 30-year population-based study. Ann Intern Med.

[29] James A.H. (2009). Venous thromboembolism in pregnancy. Arterioscler Thromb Vasc Biol.

[30] Lindsay L., Calvert C., Shi T. (2023). Neonatal and maternal outcomes following SARS-CoV-2 infection and COVID-19 vaccination: a population-based matched cohort study. Nat Commun.

[31] Gomes M.P.V., Deitcher S.R. (2004). Risk of venous thromboembolic disease associated with hormonal contraceptives and hormone replacement therapy: a clinical review. Arch Intern Med.

[32] Wesselink A.K., Hatch E.E., Rothman K.J. (2022). A prospective cohort study of COVID-19 vaccination, SARS-CoV-2 infection, and fertility. Am J Epidemiol.

[33] Wang C., Wang M., Li G., Song B., Xing Q., Cao Y. (2023). Effects of COVID-19 vaccination on human fertility: a post-pandemic literature review. Ann Med.

[34] Hui L., Marzan M.B., Rolnik D.L. (2022). Reductions in stillbirths and preterm birth in COVID-19–vaccinated women: a multicenter cohort study of vaccination uptake and perinatal outcomes. Am J Obstet Gynecol.

[35] Hernán M.A. (2010). The hazards of hazard ratios. Epidemiology.

[36] National Childbirth Trust Home birth FAQs. https://www.nct.org.uk/labour-birth/deciding-where-give-birth/giving-birth-home/home-birth-faqs.

[37] Aughey H., Jardine J., Knight H. (2023). Iatrogenic and spontaneous preterm birth in England: a population-based cohort study. BJOG.

