# Supplementary material for: COVID-19 vaccination and birth outcomes of 186,990 women vaccinated before pregnancy: an England-wide cohort study
Source: Lancet Reg Health Eur. 2024 Aug 13;45:101025. doi: 10.1016/j.lanepe.2024.101025 (PMC11838104; doi:10.1016/j.lanepe.2024.101025)

**Supplementary Materials**

**Supplementary Methods**

**Covariates**

Important covariates that could confound the association between exposure to vaccines and birth outcomes were pre-specified in the study protocol, selected according to the modified disjunctive cause criterion. These included socio-demographic variables, history of COVID-19 infection, reproductive history and comorbidities. We defined covariates from primary care, hospital admissions, community drug dispensing and COVID-19 laboratory testing data using phenotyping algorithms verified by specialist clinicians (see study protocol). We defined maternal age, county of residence, deprivation, and smoking status as the latest recorded in primary care records before study start date. Age and ethnicity were derived from the most recent non-missing value across primary care and secondary care, with preference given to primary care in the event of a match on the same date. A missing data category was added to ethnicity and smoking status covariates.

During the vaccination roll-out programme, individuals were assigned a Joint Committee on Vaccination and Immunisation (JCVI) grouping, to ensure the most vulnerable received the vaccination first. We used a previously published method to identify patients on the national Shielded Patient List, containing those with specified underlying conditions considered to make them clinically extremely vulnerable to the development of severe COVID-19. These categories were established according to the individual's age as of the dates of the first (31 March 2021) and second (01 July 2021) phases of vaccination. From the primary care data, we extracted information pertaining to the "high risk of complication," "pregnancy," and "at risk" indicators for the study cohort. Subsequently, based on the individual's age as of the first/second phase of vaccination and the presence of high-risk, pregnancy, and at-risk indicators, each person was allocated to the most relevant of the 12 JCVI priority groups. We defined a history of coronavirus (SARS-CoV-2) infection as either a positive COVID-19 antigen test or a confirmed COVID-19 diagnosis in primary care or hospital admission records.

To account for changes in rates of the outcome event over calendar time, due to pandemic-related factors such as changes in infection rates, viral variants, healthcare provision, etc, we included calendar month of estimated pregnancy start date as a key covariate.

Comorbidities were categorised as any record of a history of depression or anxiety, obesity, hypertensive disorder, diabetes, chronic kidney disease, polycystic ovary syndrome (PCOS), deep vein thrombosis (DVT), pulmonary embolism, thrombophilia or venous thrombotic events in primary care and/or hospital admission data before the study start date, including data for any surgeries in the last year. We further adjusted for prior medication use as indication of existing or past morbidity, derived from community dispensing data. British National Formulary (BNF) codes were used to define the total number of types of medication dispensed in the following groups: combined oral contraceptives, antihypertensives, antiplatelets, lipid lowering agents, immunosuppressants and oral anticoagulants.

**Statistical analysis**

Pre-specified secondary analyses were performed to examine differences according to COVID-19 vaccine administration (differences in exposed groups), according to vaccine type and combinations of total number of doses and timing of vaccination. Further analyses included restricting to the sample with complete data on gestational age, and sensitivity analyses censoring at COVID-19 infection to differentiate between consequences of severe disease and vaccination, and stratifying on JCVI priority group for the women receiving the AstraZeneca vaccine in analyses examining venous thromboembolism risk.

We employed a lifetable approach to ascertain the Absolute Excess Risk (AER). Our methodology involved constructing a lifetable spanning 84 to 343 days to encapsulate the follow-up period. The incidence rate among the unexposed cohort was constant. Initially, we determined the cumulative survival probability for the unexposed group. Subsequently, we calculated the incidence rate for the exposed group by multiplying the Hazard Ratio (HR) by the incidence rate of the unexposed group. We then computed the cumulative survival probability for the exposed group. The excess risk was quantified as the difference between the cumulative survival probability of the exposed and unexposed groups. We then multiplied this difference by the total number of individuals in the exposed cohort, and computed a weighted average to yield the Absolute Excess Risk. The resulting figure was expressed as the number of excess events per 100,000 vaccinations prior to pregnancy (AER/total exposed * 100,000). Estimates are presented in the Results section of the main manuscript (text).

For disclosure control, any cell number <10 is not reported exactly, but as <10, and any number ≥10 has been rounded up to the nearest multiple of 5.

*Deviations from prespecified protocol*

In this paper, we concentrate on the effect of first dose of COVID-19 vaccine before pregnancy on birth outcomes and venous thromboembolism as an adverse pregnancy-related outcome.

Supplementary Table 1: The RECORD statement – checklist of items, extended from the STROBE statement, that should be reported in observational studies using routinely collected health data.

|  | **Item No.** | **STROBE items** | **Location in manuscript** | **RECORD items** | **Location in manuscript** |  |
| --- | --- | --- | --- | --- | --- | --- |
| **Title and abstract** | | | | | | |
|  | 1 | (a) Indicate the study’s design with a commonly used term in the title or the abstract (b) Provide in the abstract an informative and balanced summary of what was done and what was found | Title and abstract | RECORD 1.1: The type of data used should be specified in the title or abstract. When possible, the name of the databases used should be included.  RECORD 1.2: If applicable, the geographic region and timeframe within which the study took place should be reported in the title or abstract.  RECORD 1.3: If linkage between databases was conducted for the study, this should be clearly stated in the title or abstract. | Abstract  Title  Abstract |  |
| **Introduction** | | | | | | |
| Background rationale | 2 | Explain the scientific background and rationale for the investigation being reported | Introduction |  |  |  |
| Objectives | 3 | State specific objectives, including any prespecified hypotheses | Introduction |  |  |  |
| **Methods** | | | | | | |
| Study Design | 4 | Present key elements of study design early in the paper | Methods |  |  |  |
| Setting | 5 | Describe the setting, locations, and relevant dates, including periods of recruitment, exposure, follow-up, and data collection | Methods |  |  |  |
| Participants | 6 | *(a) Cohort study* - Give the eligibility criteria, and the sources and methods of selection of participants. Describe methods of follow-up  *Case-control study* - Give the eligibility criteria, and the sources and methods of case ascertainment and control selection. Give the rationale for the choice of cases and controls  *Cross-sectional study* - Give the eligibility criteria, and the sources and methods of selection of participants  *(b) Cohort study* - For matched studies, give matching criteria and number of exposed and unexposed  *Case-control study* - For matched studies, give matching criteria and the number of controls per case | Methods | RECORD 6.1: The methods of study population selection (such as codes or algorithms used to identify subjects) should be listed in detail. If this is not possible, an explanation should be provided.  RECORD 6.2: Any validation studies of the codes or algorithms used to select the population should be referenced. If validation was conducted for this study and not published elsewhere, detailed methods and results should be provided.  RECORD 6.3: If the study involved linkage of databases, consider use of a flow diagram or other graphical display to demonstrate the data linkage process, including the number of individuals with linked data at each stage. | Methods and GitHub repositories  Methods  Methods |  |
| Variables | 7 | Clearly define all outcomes, exposures, predictors, potential confounders, and effect modifiers. Give diagnostic criteria, if applicable. | Methods and supplementary material | RECORD 7.1: A complete list of codes and algorithms used to classify exposures, outcomes, confounders, and effect modifiers should be provided. If these cannot be reported, an explanation should be provided. | Github repository |  |
| Data sources/ measurement | 8 | For each variable of interest, give sources of data and details of methods of assessment (measurement).  Describe comparability of assessment methods if there is more than one group | Methods and supplementary material |  |  |  |
| Bias | 9 | Describe any efforts to address potential sources of bias | Methods and supplementary material |  |  |  |
| Study size | 10 | Explain how the study size was arrived at | Methods |  |  |  |
| Quantitative variables | 11 | Explain how quantitative variables were handled in the analyses. If applicable, describe which groupings were chosen, and why | Statistical analyses |  |  |  |
| Statistical methods | 12 | (a) Describe all statistical methods, including those used to control for confounding  (b) Describe any methods used to examine subgroups and interactions  (c) Explain how missing data were addressed  (d) *Cohort study* - If applicable, explain how loss to follow-up was addressed  *Case-control study* - If applicable, explain how matching of cases and controls was addressed  *Cross-sectional study* - If applicable, describe analytical methods taking account of sampling strategy  (e) Describe any sensitivity analyses | Statistical analyses |  |  |  |
| Data access and cleaning methods |  | .. |  | RECORD 12.1: Authors should describe the extent to which the investigators had access to the database population used to create the study population.  RECORD 12.2: Authors should provide information on the data cleaning methods used in the study. | Methods  Methods |  |
| Linkage |  | .. |  | RECORD 12.3: State whether the study included person-level, institutional-level, or other data linkage across two or more databases. The methods of linkage and methods of linkage quality evaluation should be provided. | Methods and links to external sources |  |
| **Results** | | | | | | |
| Participants | 13 | (a) Report the numbers of individuals at each stage of the study (*e.g.*, numbers potentially eligible, examined for eligibility, confirmed eligible, included in the study, completing follow-up, and analysed)  (b) Give reasons for non-participation at each stage.  (c) Consider use of a flow diagram | Paragraph 1 | RECORD 13.1: Describe in detail the selection of the persons included in the study (*i.e.,* study population selection) including filtering based on data quality, data availability and linkage. The selection of included persons can be described in the text and/or by means of the study flow diagram. | Paragraph 1 |  |
| Descriptive data | 14 | (a) Give characteristics of study participants (*e.g.*, demographic, clinical, social) and information on exposures and potential confounders  (b) Indicate the number of participants with missing data for each variable of interest  (c) *Cohort study* - summarise follow-up time (*e.g.*, average and total amount) | Table 1, Supp table 2 |  |  |  |
| Outcome data | 15 | *Cohort study* - Report numbers of outcome events or summary measures over time  *Case-control study* - Report numbers in each exposure category, or summary measures of exposure  *Cross-sectional study* - Report numbers of outcome events or summary measures | Table 2 |  |  |  |
| Main results | 16 | (a) Give unadjusted estimates and, if applicable, confounder-adjusted estimates and their precision (e.g., 95% confidence interval). Make clear which confounders were adjusted for and why they were included  (b) Report category boundaries when continuous variables were categorized  (c) If relevant, consider translating estimates of relative risk into absolute risk for a meaningful time period | Table 3 |  |  |  |
| Other analyses | 17 | Report other analyses done—e.g., analyses of subgroups and interactions, and sensitivity analyses | Results |  |  |  |
| **Discussion** | | | | | | |
| Key results | 18 | Summarise key results with reference to study objectives | Paragraph 1, discussion |  |  |  |
| Limitations | 19 | Discuss limitations of the study, taking into account sources of potential bias or imprecision. Discuss both direction and magnitude of any potential bias | Strengths and limitations section | RECORD 19.1: Discuss the implications of using data that were not created or collected to answer the specific research question(s). Include discussion of misclassification bias, unmeasured confounding, missing data, and changing eligibility over time, as they pertain to the study being reported. | Strengths and limitations section |  |
| Interpretation | 20 | Give a cautious overall interpretation of results considering objectives, limitations, multiplicity of analyses, results from similar studies, and other relevant evidence | Conclusion |  |  |  |
| Generalisability | 21 | Discuss the generalisability (external validity) of the study results | Strengths and limitations section |  |  |  |
| **Other Information** | | | | | | |
| Funding | 22 | Give the source of funding and the role of the funders for the present study and, if applicable, for the original study on which the present article is based | Funding details |  |  |  |
| Accessibility of protocol, raw data, and programming code |  | .. |  | RECORD 22.1: Authors should provide information on how to access any supplemental information such as the study protocol, raw data, or programming code. | Links throughout paper, including to GitHub repository |  |

Supplementary Table 2: Description of covariates defined at estimated pregnancy start date, and used in the analysis

| **Variable** | **Description** | **Source** | **Codes used** |
| --- | --- | --- | --- |
| Age at start of pregnancy | Continuous variable | Primary or secondary care data |  |
| Deprivation | Categorical variable (quintiles) 1- most deprived; 5 least deprived | Primary or secondary care | Based on LSOA reference table |
| Region |  | Primary or secondary care | Based on LSOA reference table |
| Ethnicity | White  Asian  Black  Mixed  Others  Unknown | Primary or secondary care |  |
| BMI Obesity | Binary variables | Primary care, Secondary care, and operation dataset | DMD & SNOMED codes from GDPPR  ICD10 from HES_APC  OPCS4 from HES_APC_OPER |
| Covid-19 positive test or diagnosis | Binary variables | Primary care, Secondary care, and operation dataset  Covid testing data | DMD & SNOMED codes from GDPPR  ICD10 from HES_APC  OPCS4 from HES_APC_OPER |
| Depression Anxiety | Binary variables (ever) | Primary care, Secondary care, and operation dataset | DMD & SNOMED codes from GDPPR  ICD10 from HES_APC  OPCS4 from HES_APC_OPER |
| Diabetes Mellitus | Binary variables  (ever) | Primary care, Secondary care, and operation dataset | DMD & SNOMED codes from GDPPR  ICD10 from HES_APC  OPCS4 from HES_APC_OPER |
| Hypertensive disorder | Binary variables  (ever) | Primary care, Secondary care, and operation dataset | DMD & SNOMED codes from GDPPR  ICD10 from HES_APC  OPCS4 from HES_APC_OPER |
| Deep Vein Thrombosis and Pulmonary Embolism | Binary variables  (ever) | Primary care, Secondary care, and operation dataset | DMD & SNOMED codes from GDPPR  ICD10 from HES_APC  OPCS4 from HES_APC_OPER |
| Polycystic ovary syndrome | Binary variables  (ever) | Primary care, Secondary care, and operation dataset | DMD & SNOMED codes from GDPPR  ICD10 from HES_APC  OPCS4 from HES_APC_OPER |
| Stillbirth | Binary variables (ever) | Primary care, Secondary care, and operation dataset | DMD & SNOMED codes from GDPPR  ICD10 from HES_APC  OPCS4 from HES_APC_OPER |
| Venous thrombotic events | Binary variables  (ever) | Primary care, Secondary care, and operation dataset | DMD & SNOMED codes from GDPPR  ICD10 from HES_APC  OPCS4 from HES_APC_OPER |
| Thrombophilia | Binary variables  (ever) | Primary care, Secondary care, and operation dataset | DMD & SNOMED codes from GDPPR  ICD10 from HES_APC  OPCS4 from HES_APC_OPER |
| Chronic kidney disease | Binary variables  (ever) | Primary care, Secondary care, and operation dataset | DMD & SNOMED codes from GDPPR  ICD10 from HES_APC  OPCS4 from HES_APC_OPER |
| R/x lipid lowering | Binary variable (prior to pregnancy start date) | Medicine dispensing data | BNF codes from dispensing dataset |
| R/x antiplatelets | Binary variable (prior to pregnancy start date) | Medicine dispensing data | BNF codes from dispensing dataset |
| R/x combined oral contraceptive pills | Binary variable (prior to pregnancy start date) | Medicine dispensing data | BNF codes from dispensing dataset |
| R/x anticoagulant | Binary variable (prior to pregnancy start date) | Medicine dispensing data | BNF codes from dispensing dataset |
| Smoking status | Categories : Current, ex, or never (and unknown) | Primary care data, Secondary care, or operations | DMD & SNOMED codes from GDPPR  ICD10 from HES_APC  OPCS4 from HES_APC_OPER |
| Surgery in the last year |  | Operations data (secondary care) | OPCS4 from HES_APC_OPER |
| JCVI grouping | 1-12 groups | Vaccination dataset, Primary care, Secondary care, delivery dataset | Vaccination data, high risk pregnancy, and at-risk flag |

H/o- Medical History; R/x – Treatment history; LSOA – Lower layer Super Output Area; DMD – Dictionary of Medicines and Devices; SNOMED- Systematic NOmenclature of MEDicines; OPSC- Operations Procedure Codes; ICD-10- International Classification of Diseases -10; GDPPR- General Practice Extraction Service (GPES) Data for Pandemic Planning and Research; HES_APC- Hopsital Episode Statistics Admitted Patient Care; HES_APC_OPER - Hopsital Episode Statistics Admitted Patient Care Operations; BMI- Body Mass Index; JCVI: Joint Committee on Vaccination and Immunisation

Supplementary Table 3: Confounder distribution by vaccine type (first dose received prior to onset of pregnancy)

| **Characteristic** |  | **Exposure to viral-vector vaccine (ChAdOX1)**  **(n=24,565)** | **Exposure to mRNA vaccine**  **(BNT162b2)**  **(n=27,945)** |
| --- | --- | --- | --- |
| **Age (years)†** |  | 30·9 (5·5) | 31·2 (4·9) |
| **Age group (years)** | 18 – 24 | 3,220 (13·1%) | 2,725 (9·8%) |
|  | 25 – 29 | 6,340 (25·8%) | 6,900 (24·7%) |
|  | 30 – 34 | 8,590 (35·0%) | 11,175 (40·0%) |
|  | 35 – 39 | 4,800 (19·5%) | 6,235 (22·3%) |
|  | 40- 45 | 1,615 (6·6%) | 910 (3·3%) |
| **Ethnicity** | White | 19,545 (79·6%) | 22,595 (80·9%) |
|  | Asian or Asian British | 3,000 (12·2%) | 3,245 (11·6%) |
|  | Black or Black British | 785 (3·2%) | 655 (2·3%) |
|  | Mixed | 430 (1·8%) | 510 (1·8%) |
|  | Other | 760 (3·1%) | 885 (3·2%) |
|  | Unknown | 45 (0·2%) | 50 (0·2%) |
| **Region** | East midlands | 2,365 (9·6%) | 2,305 (8·3%) |
|  | East of England | 2,915 (11·9%) | 3,305 (11·8%) |
|  | London | 3,580 (14·6%) | 3,840 (13·7%) |
|  | North East | 1,255 (5·1%) | 1,510 (5·4%) |
|  | North West | 3,230 (13·2%) | 3,490 (12·5%) |
|  | South East | 3,875 (15·8%) | 4,910 (17·6%) |
|  | South West | 2,150 (8·8%) | 3,210 (11·5%) |
|  | West Midlands | 2,730 (11·1%) | 2,620 (9·4%) |
|  | Yorkshire and The Humber | 2,460 (10·0%) | 2,745 (9·8%) |
| **Deprivation index** | 1-2 (Most deprived) | 5,620 (22·9%) | 4,895 (17·5%) |
|  | 3-4 | 5,275 (21·5%) | 5,410 (19·4%) |
|  | 5-6 | 4,765 (19·4%) | 5,950 (21·3%) |
|  | 7-8 | 4,665 (19·0%) | 5,905 (21·1%) |
|  | 9-10 (Least deprived) | 4,235 (17·2%) | 5,780 (20·7%) |
| **Parity** | Multiparous | 17,465 (71·1%) | 18,055 (64·6%) |
|  | Nulliparous | 7,100 (28·9%) | 9,885 (35·4%) |
| **Reproductive history** | Stillbirth | 175 (0·7%) | 165 (0·6%) |
| **JCVI group** | Group 4 (vulnerable) | 990 (4·0%) | 680 (2·4%) |
|  | Group 10 (age>=40) | 1,660 (6·8%) | 980 (3·5%) |
|  | Group 11(age > =30) | 13,070 (53·2%) | 17,280 (61·8%) |
|  | Group 12 (age>=18) | 8,840 (36%) | 9,005 (32·2%) |
| **Medical history** | Prior COVID-19 infection | 2,125 (8·7%) | 2,065 (7·4%) |
|  | Depression/anxiety | 7,735 (31·5%) | 7,105 (25·4%) |
|  | Obesity | 4,590 (18·7%) | 3,535 (12·6%) |
|  | Hypertensive disorder | 3,790 (15·4%) | 3,550 (12·7%) |
|  | Diabetes Mellitus | 3,365 (13·7%) | 2,140 (7·7%) |
|  | Chronic kidney disease | 380 (1·5%) | 280 (1·0%) |
|  | PCOS | 985 (4%) | 880 (3·1%) |
|  | DVT/ Pulmonary embolism | 250 (1·0%) | 135 (0·5%) |
|  | Thrombophilia | 75 (0·3%) | 80 (0·3%) |
|  | Venous thrombotic event | 250 (1·0%) | 135 (0·5%) |
| **Treatment history** | Surgery in previous year | 3,755 (15·3%) | 3,540 (12·7%) |
|  | Combined oral contraceptive | 1,230 (5·0%) | 1,415 (5·1%) |
|  | BP Lowering drugs | 450 (1·8%) | 330 (1·2%) |
|  | Lipid lowering drugs | 120 (0·5%) | 95 (0·3%) |
|  | Immunosuppressants | 110 (0·4%) | 90 (0·3%) |
|  | Anticoagulant | 85 (0·3%) | 50 (0·2%) |
| **Smoking status** | Current | 4,245 (17·3%) | 3,580 (12·8%) |
|  | Never | 15,890 (64·7%) | 19,485 (69·7%) |
|  | Ex-smoker | 3,980 (16·2%) | 4,275 (15·3%) |
|  | Missing | 445 (1·8%) | 600 (2·1%) |

†Mean(SD) reported for continuous variable

Supplementary Table 4: Hazard Ratios of pregnancy outcomes following pre-pregnancy vaccination exposure among pregnancies in England with an estimated start date between 08 December 2020 and 31 December 2021 (primary and secondary analyses)

| **Outcome** | **Exposure subgroup** |  | **Events (n)** | **Hazard Ratios (95% CI) [p-value]** | | |
| --- | --- | --- | --- | --- | --- | --- |
|  |  | **Follow up time interval (gest·weeks)** |  | **Unadjusted** | **Age-adjusted** | **Maximally Adjusted** |
| Preterm birth | First dose before pregnancy – Primary analysis | 24 to <32 | 3340 | 1∙39 (1∙29, 1∙49) [<0∙0001] | 1∙36 (1∙27, 1∙46) [<0∙0001] | 0·74 (0·63,0·88) [0·00045] |
|  |  | 32 to 36 | 9510 | 1∙31 (1∙26, 1∙37) [<0∙0001] | 1∙29 (1∙23, 1∙34) [<0∙0001] | 0·98 (0·93,1·03) [0·49] |
|  | -of which : viral-vector vaccine (ChAdOx1) | 24 to <32 | 2645 | 1∙25 (1∙13, 1·38) [<0∙0001] | 1·21 (1·10, 1·34) [<0∙0001] | 0·72 (0·58,0·91) [0·0049] |
|  |  | 32 to 36 | 7790 | 1∙33 (1∙25, 1∙40) [<0∙0001] | 1∙29 (1∙22, 1∙37) [<0∙0001] | 1·02 (0·95,1·08) [0·63] |
|  | -of which : mRNA vaccine (BNT162b2) | 24 to <32 | 2820 | 1∙49 (1∙37, 1∙63) [<0∙0001] | 1∙46 (1∙34, 1∙59) [<0∙0001] | 0·79 (0·65,0·97) [0·025] |
|  |  | 32 to 36 | 7940 | 1∙29 (1∙22, 1∙36) [<0∙0001] | 1∙26 (1∙20, 1∙33) [<0∙0001] | 0·93 (0·87,0·99) [0·016] |
|  | Two dose before pregnancy | 24 to <32 | 3150 | 1∙41 (1∙30, 1∙52) [<0∙0001] | 1∙38 (1∙28, 1∙49) [<0∙0001] | 0·74 (0·62,0·89) [0·0011] |
|  |  | 32 to 36 | 8980 | 1∙32 (1∙27, 1∙39) [<0∙0001] | 1∙30 (1∙24, 1∙36) [<0∙0001] | 0·96 (0·91,1·01) [0·14] |
|  | Three doses by 24 weeks gestation | 24 to <32 | 2470 | 4∙14 (3∙68, 4∙66) [<0∙0001] | 3∙98 (3∙53, 4∙66) [<0∙0001] | 1·04 (0·79,1·37) [0·76] |
|  |  | 32 to 36 | 6770 | 2∙37 (2∙16, 2∙60) [<0∙0001] | 2∙28 (2∙07, 2∙50) [<0∙0001] | 0·98 (0·88,1·09) [0·71] |
|  | First dose before pregnancy (complete gestational age) | 24 to <32 | 2915 | 1∙33 (1∙23, 1·44) [<0∙0001] | 1∙31 (1·21,1·41) [<0∙0001] | 0·76 (0·64,0·91) [0·0028] |
|  |  | 32 to 36 | 8325 | 1∙26 (1∙21, 1∙32) [<0∙0001] | 1∙24 (1∙18, 1∙30) [<0∙0001] | 0∙97 (0∙92, 1∙03) [0∙33] |
| Small for gestational age | First dose before pregnancy – Primary analysis | 24 to <32 | 150 | 1∙27 (0∙91, 1∙79) [0·16] | 1∙29 (0∙92, 1∙81) [0·14] | 0∙87 (0∙61, 1∙25) [0·45] |
|  |  | 32 to 36 | 405 | 1∙07 (0∙86, 1∙32) [0·55] | 1∙08 (0∙87, 1∙34) [0·48] | 1∙00 (0∙80, 1∙25) [0·99] |
|  |  | >36 | 6680 | 0∙85 (0∙81, 0∙90) [<0∙0001] | 0∙86 (0∙82, 0∙91) [<0∙0001] | 0∙94 (0∙88, 1∙00) [0·06] |
|  | -of which : viral-vector vaccine (ChAdOx1) | 24 to <32 | 125 | 1·38 (0·89,2·14) [0·15] | 1·39 (0·9,2·16) [0·14] | 0·96 (0·61,1·52) [0·87] |
|  |  | 32 to 36 | 345 | 1·13 (0·86,1·5) [0·38] | 1·15 (0·87,1·52) [0·34] | 1·05 (0·79,1·4) [0·72] |
|  |  | >36 | 5835 | 0·85 (0·79,0·92) [<0·0001] | 0·86 (0·8,0·93) [0·00024] | 0·93 (0·85,1·01) [0·1] |
|  | -of which : mRNA vaccine (BNT162b2) | 24 to <32 | 125 | 1·22 (0·79,1·89) [0·38] | 1·24 (0·8,1·92) [0·34] | 0·85 (0·53,1·34) [0·48] |
|  |  | 32 to 36 | 345 | 1·02 (0·77,1·35) [0·88] | 1·04 (0·79,1·37) [0·78] | 0·97 (0·73,1·3) [0·85] |
|  |  | >36 | 5945 | 0·85 (0·79,0·91) [<0·0001] | 0·86 (0·8,0·93) [1e-04] | 0·93 (0·86,1·01) [0·1] |
|  | Two dose before pregnancy | 24 to <32 | 145 | 1·38 (0·97,1·97) [0·071] | 1·4 (0·99,2) [0·059] | 0·91 (0·62,1·33) [0·63] |
|  |  | 32 to 36 | 380 | 1·02 (0·81,1·29) [0·87] | 1·04 (0·82,1·31) [0·77] | 0·94 (0·74,1·2) [0·63] |
|  |  | >36 | 6395 | 0·83 (0·78,0·89) [<0·0001] | 0·85 (0·8,0·9) [<0·0001] | 0·92 (0·85,0·99) [0·019] |
|  | Three doses by 24 weeks gestation | 24 to <32 | 115 | 3·74 (2·1,6·67) [<0·0001] | 3·83 (2·15,6·83) [<0·0001] | 1·32 (0·69,2·51) [0·41] |
|  |  | 32 to 36 | 310 | 2·41 (1·58,3·69) [<0·0001] | 2·47 (1·62,3·78) [<0·0001] | 1·84 (1·19,2·85) [0·0066] |
|  |  | >36 | 5220 | 0·69 (0·56,0·85) [0·00032] | 0·71 (0·58,0·87) [0·00081] | 0·72 (0·58,0·89) [0·0023] |
|  | First dose before pregnancy (complete gestational age) | 24 to <32 | 150 | 1·25 (0·89,1·75) [0·2] | 1·25 (0·89,1·76) [0·2] | 0·89 (0·62,1·28) [0·51] |
|  |  | 32 to 36 | 405 | 1·05 (0·84,1·29) [0·69] | 1·05 (0·85,1·3) [0·66] | 1 (0·8,1·24) [0·97] |
|  |  | >36 | 6680 | 0·86 (0·81,0·91) [<0·0001] | 0·86 (0·81,0·91) [<0·0001] | 0·95 (0·89,1·02) [0·14] |
| Stillbirth | First dose before pregnancy – Primary analysis | | 485 | 0∙84 (0∙68, 1∙04) [0·11] | 0∙78 (0∙63, 0∙97) [0·023] | 0∙86 (0∙67, 1∙10) [0·22] |
|  | -of which : viral-vector vaccine (ChAdOx1) | | 435 | 1·03 (0·79,1·34) [0·86] | 0·95 (0·73,1·25) [0·73] | 1·02 (0·75,1·38) [0·91] |
|  | -of which : mRNA vaccine (BNT162b2) | | 425 | 0·69 (0·51,0·93) [0·016] | 0·63 (0·47,0·86) [0·0029] | 0·72 (0·52,1) [0·052] |
|  | Two dose before pregnancy | | 475 | 0·9 (0·72,1·12) [0·32] | 0·82 (0·66,1·03) [0·093] | 0·92 (0·7,1·2) [0·53] |
|  | Three doses by 24 weeks gestation | | 385 | 1·28 (0·74,2·22) [0·39] | 1·14 (0·65,1·99) [0·64] | 1·22 (0·66,2·25) [0·53] |
|  | First dose before pregnancy (complete gestational age) | | 265 | 0·79 (0·59,1·05) [0·1] | 0·74 (0·55,0·99) [0·04] | 0·72 (0·51,1·01) [0·058] |
| Venous events | First dose before pregnancy – Primary analysis | | 300 | 1∙40 (1∙10, 1∙77) [0·0056] | 1∙41 (1∙11, 1∙80) [0·0055] | 1∙27 (0∙95, 1∙69) [0·11] |
|  | -of which : viral-vector vaccine (ChAdOx1) | | 260 | 1·86 (1·41,2·47) [<0·0001] | 1·89 (1·42,2·51) [<0·0001] | 1·54 (1·1,2·16) [0·011] |
|  | JCVI group 4* | | <10 |  |  |  |
|  | JCVI group 10* | | <10 |  |  |  |
|  | JCVI group 11* | | 114 | 1·97 (1·32, 2·93) [0·0009] | 1·94 (1·30, 2·89) [0·0012] | 1·88 (1·14, 3·11) [0·014] |
|  | JCVI group 12* | | 133 | 1·91 (1·24, 2·95) [0·0035] | 1·91 (1·24, 2·96) [0·0036] | 1·33 (0·82, 2·16) [0·26] |
|  | -of which : mRNA vaccine (BNT162b2) | | 235 | 1·03 (0·74,1·45) [0·86] | 1·02 (0·72,1·43) [0·92] | 1·02 (0·7,1·5) [0·91] |
|  | Two dose before pregnancy | | 280 | 1·4 (1·09,1·8) [0·0089] | 1·42 (1·09,1·84) [0·0085] | 1·35 (0·98,1·85) [0·067] |
|  | Three doses by 24 weeks gestation | | 205 | 1·83 (1·02,3·28) [0·042] | 1·83 (1·02,3·3) [0·044] | 1·76 (0·88,3·55) [0·11] |
|  | First dose before pregnancy (complete gestational age) | | 220 | 1·38 (1·04,1·81) [0·024] | 1·4 (1·05,1·85) [0·02] | 1·24 (0·88,1·75) [0·22] |

*JCVI group 4. Vulnerable, JCVI group 10 All those aged >=40 years, 11. All those aged 30 to 39 years, JCVI group 12. All those aged 18 to 29 years

Supplementary Table 5: Hazard ratio of pregnancy outcomes following pre-pregnancy vaccination exposure (primary analysis and the subgroup analyses) among pregnancies in England with an estimated start date between 08 December 2020 and 31 December 2021

| **Outcome** | **Subgroup** | **Follow up**  **time interval**  **(gestational week)** | **Unadjusted** | **Age-adjusted** | **Maximally** | **Interaction p-value** |
| --- | --- | --- | --- | --- | --- | --- |
| **Preterm birth** | First dose before pregnancy (PRIMARY ANALYSIS) | 24 to 32 weeks | 1·39 (1·29, 1·49) [<0·0001] | 1·36 (1·27, 1·46) [<0·0001] | 0·74 (0·63, 0·88) [0.00045] |  |
|  |  | 32 to 36 weeks | 1·31 (1·26, 1·37) [<0·0001] | 1·29 (1·23, 1·34) [<0·0001] | 0·98 (0·93, 1·03) [0·49] |  |
|  | Age:18-24 | 24 to 32 weeks | 1·65 (1·37,1·98) [<0·0001] | 1·66 (1·38,2) [<0·0001] | 0·48 (0·3,0·78) [0·0028] | <0·0001 |
|  |  | 32 to 36 weeks | 1·43 (1·28,1·6) [<0·0001] | 1·44 (1·29,1·61) [<0·0001] | 1·02 (0·9,1·16) [0·77] |  |
|  | 25-29 | 24 to 32 weeks | 1·58 (1·38,1·8) [<0·0001] | 1·57 (1·37,1·8) [<0·0001] | 0·75 (0·54,1·05) [0·09] |  |
|  |  | 32 to 36 weeks | 1·32 (1·22,1·44) [<0·0001] | 1·32 (1·21,1·43) [<0·0001] | 0·99 (0·9,1·09) [0·87] |  |
|  | 30-34 | 24 to 32 weeks | 1·27 (1·12,1·44) [0·00025] | 1·26 (1·11,1·43) [0·00029] | 0·83 (0·61,1·12) [0·22] |  |
|  |  | 32 to 36 weeks | 1·35 (1·25,1·45) [<0·0001] | 1·35 (1·25,1·45) [<0·0001] | 1·02 (0·93,1·12) [0·62] |  |
|  | 35-39 | 24 to 32 weeks | 1·17 (1,1·37) [0·051] | 1·17 (1,1·37) [0·053] | 0·82 (0·57,1·17) [0·27] |  |
|  |  | 32 to 36 weeks | 1·14 (1·04,1·26) [0·0079] | 1·14 (1·03,1·26) [0·0087] | 0·95 (0·84,1·07) [0·41] |  |
|  | 40-45 | 24 to 32 weeks | 0·90 (0·63,1·29) [0·57] | 0·90 (0·63,1·30) [0·57] | 0·36 (0·15,0·88) [0·024] |  |
|  |  | 32 to 36 weeks | 0·99 (0·81,1·22) [0·95] | 0·99 (0·81,1·22) [0·95] | 0·88 (0·67,1·15) [0·34] |  |
|  | Deprivation: High | 24 to 32 weeks | 1·45 (1·3,1·61) [<0·0001] | 1·43 (1·29,1·59) [<0·0001] | 0·65 (0·5,0·85) [0·0012] | 0·74 |
|  |  | 32 to 36 weeks | 1·33 (1·25,1·42) [<0·0001] | 1·31 (1·24,1·4) [<0·0001] | 0·97 (0·9,1·05) [0·43] |  |
|  | Low | 24 to 32 weeks | 1·45 (1·32,1·6) [<0·0001] | 1·41 (1·28,1·56) [<0·0001] | 0·76 (0·6,0·95) [0·017] |  |
|  |  | 32 to 36 weeks | 1·38 (1·3,1·47) [<0·0001] | 1·34 (1·26,1·42) [<0·0001] | 0·99 (0·92,1·06) [0·74] |  |
|  | Ethnicity: White | 24 to 32 weeks | 1·46 (1·35,1·59) [<0·0001] | 1·44 (1·33,1·57) [<0·0001] | 0·73 (0·6,0·89) [0·0023] | 0·81 |
|  |  | 32 to 36 weeks | 1·26 (1·2,1·32) [<0·0001] | 1·24 (1·18,1·30) [<0·0001] | 0·97 (0·92,1·03) [0·38] |  |
|  | Other | 24 to 32 weeks | 1·25 (1·07,1·47) [0·0052] | 1·2 (1·02,1·41) [0·027] | 0·67 (0·47,0·95) [0·027] |  |
|  |  | 32 to 36 weeks | 1·45 (1·32,1·6) [<0·0001] | 1·39 (1·25,1·53) [<0·0001] | 1·02 (0·91,1·15) [0·75] |  |
|  | Unknown | 24 to 32 weeks | 1·26 (0·88,1·80) [0·21] | 1·22 (0·85,1·75) [0·29] | 0·95 (0·66,1·36) [0·78] |  |
|  |  | 32 to 36 weeks | 1·43 (1·14,1·81) [0·0023] | 1·39 (1·09,1·76) [0·007] | 0·98 (0·77,1·23) [0·83] |  |
|  | Asian | 24 to 32 weeks | 1·39 (1·14,1·7) [0·0013] | 1·3 (1·06,1·58) [0·012] | 0·67 (0·41,1·09) [0·11] |  |
|  |  | 32 to 36 weeks | 1·47 (1·30,1·65) [<0·0001] | 1·37 (1·21,1·54) [<0·0001] | 1 (0·86,1·16) [0·99] |  |
|  | Black | 24 to 32 weeks | 1·48 (1·08,2·02) [0·015] | 1·44 (1·05,1·98) [0·023] | 0·94 (0·49,1·79) [0·84] |  |
|  |  | 32 to 36 weeks | 1·37 (1·08,1·75) [0·01] | 1·34 (1·05,1·72) [0·018] | 1·06 (0·82,1·39) [0·65] |  |
|  | SARS-CoV2 before vaccination | 24 to 32 weeks | 1·37 (1·04,1·82) [0·028] | 1·34 (1·01,1·77) [0·044] | 1·35 (0·65,2·79) [0·42] | 0·084 |
|  |  | 32 to 36 weeks | 1·42 (1·21,1·67) [<0·0001] | 1·39 (1·18,1·63) [<0·0001] | 1·1 (0·91,1·33) [0·35] |  |
|  | no SARS-CoV2 before vaccination | 24 to 32 weeks | 1·39 (1·29,1·50) [<0·0001] | 1·36 (1·27,1·47) [<0·0001] | 0·72 (0·60,0·85) [0·00017] |  |
|  |  | 32 to 36 weeks | 1·30 (1·25,1·36) [<0·0001] | 1·28 (1·22,1·34) [<0·0001] | 0·97 (0·92,1·03) [0·34] |  |
|  | Previous pregnancies | 24 to 32 weeks | 1·27 (1·16,1·38) [<0·0001] | 1·25 (1·14,1·37) [<0·0001] | 0·75 (0·61,0·92) [0·0056] | 0·00075 |
|  |  | 32 to 36 weeks | 1·26 (1·20,1·33) [<0·0001] | 1·25 (1·18,1·31) [<0·0001] | 1·00 (0·94,1·07) [0·93] |  |
|  | No previous pregnancies | 24 to 32 weeks | 1·65 (1·46,1·86) [<0·0001] | 1·60 (1·42,1·80) [<0·0001] | 0·67 (0·50,0·90) [0·0078] |  |
|  |  | 32 to 36 weeks | 1·41 (1·31,1·52) [<0·0001] | 1·37 (1·27,1·48) [<0·0001] | 0·94 (0·85,1·03) [0·15] |  |
|  | Multiple pregnancy | 24 to 32 weeks | 1·48 (1·22,1·80) [0·0001] | 1·45 (1·19,1·76) [0·00025] | 0·51 (0·30,0·88) [0·015] | <0.0001 |
|  |  | 32 to 36 weeks | 1·45 (1·28,1·63) [<0·0001] | 1·41 (1·25,1·59) [<0·0001] | 1·03 (0·89,1·19) [0·72] |  |
|  | Singleton pregnancy | 24 to 32 weeks | 1·36 (1·26,1·47) [<0·0001] | 1·34 (1·24,1·45) [<0·0001] | 0·77 (0·65,0·92) [0·0046] |  |
|  |  | 32 to 36 weeks | 1·29 (1·23,1·35) [<0·0001] | 1·27 (1·21,1·33) [<0·0001] | 0·97 (0·92,1·03) [0·31] |  |
|  | JCVI gp:4 (vulnerable) | 24 to 32 weeks | 0·96 (0·61,1·51) [0·84] | 0·97 (0·61,1·53) [0·88] | 1·15 (0·33,4·05) [0·83] | 0·60 |
|  |  | 32 to 36 weeks | 1·32 (1·02,1·7) [0·035] | 1·33 (1·03,1·73) [0·029] | 1·15 (0·84,1·57) [0·37] |  |
|  | JCVI gp:10 (age>=40) | 24 to 32 weeks | 0·82 (0·57,1·17) [0·27] | 0·81 (0·57,1·17) [0·26] | 0·31 (0·13,0·74) [0·0077] |  |
|  |  | 32 to 36 weeks | 0·97 (0·8,1·19) [0·8] | 0·97 (0·79,1·18) [0·75] | 0·86 (0·67,1·12) [0·27] |  |
|  | JCVI gp:11 (age>=30) | 24 to 32 weeks | 1·27 (1·15,1·4) [<0·0001] | 1·24 (1·13,1·37) [<0·0001] | 0·85 (0·67,1·07) [0·17] |  |
|  |  | 32 to 36 weeks | 1·28 (1·21,1·36) [<0·0001] | 1·25 (1·18,1·33) [<0·0001] | 0·99 (0·92,1·07) [0·78] |  |
|  | JCVI gp:12 (age>=18) | 24 to 32 weeks | 1·61 (1·44,1·8) [<0·0001] | 1·61 (1·44,1·8) [<0·0001] | 0·67 (0·51,0·88) [0·0045] |  |
|  |  | 32 to 36 weeks | 1·33 (1·24,1·42) [<0·0001] | 1·33 (1·24,1·43) [<0·0001] | 0·99 (0·91,1·07) [0·71] |  |
| Small for gestational age | First dose before pregnancy (PRIMARY ANALYSIS) | 24 to <32 weeks | 1·27 (0·91,1·79) [0·16] | 1·29 (0·92,1·81) [0·14] | 0·87 (0·61,1·25) [0·45] |  |
|  |  | 32 to 36 weeks | 1·07 (0·86,1·32) [0·55] | 1·08 (0·87,1·34) [0·48] | 1·00 (0·80,1·25) [0·99] |  |
|  |  | >36 weeks | 0·85 (0·81,0·9) [<0·0001] | 0·86 (0·82,0·91) [<0·0001] | 0·94 (0·88,1·00) [0·059] |  |
|  | Age:18-24 | 24 to <32 weeks | 1·33 (0·54,3·24) [0·54] | 1·33 (0·54,3·24) [0·54] | 0·79 (0·31,2·03) [0·62] | 0·97 |
|  |  | 32 to 36 weeks | 1·07 (0·63,1·84) [0·8] | 1·07 (0·63,1·84) [0·8] | 0·96 (0·56,1·66) [0·89] |  |
|  |  | >36 weeks | 0·86 (0·74,1) [0·052] | 0·86 (0·74,1) [0·052] | 0·91 (0·78,1·07) [0·27] |  |
|  | 25-29 | 24 to <32 weeks | 1·44 (0·8,2·58) [0·23] | 1·44 (0·8,2·58) [0·23] | 0·88 (0·49,1·6) [0·68] |  |
|  |  | 32 to 36 weeks | 1·06 (0·69,1·62) [0·8] | 1·06 (0·69,1·62) [0·8] | 0·99 (0·65,1·51) [0·96] |  |
|  |  | >36 weeks | 0·85 (0·76,0·95) [0·0045] | 0·85 (0·76,0·95) [0·0045] | 0·95 (0·85,1·07) [0·39] |  |
|  | 30-34 | 24 to <32 weeks | 0·82 (0·39,1·73) [0·61] | 0·82 (0·39,1·73) [0·61] | 0·62 (0·28,1·38) [0·24] |  |
|  |  | 32 to 36 weeks | 1·05 (0·71,1·56) [0·81] | 1·05 (0·71,1·56) [0·81] | 0·95 (0·63,1·43) [0·79] |  |
|  |  | >36 weeks | 0·88 (0·8,0·97) [0·0091] | 0·88 (0·8,0·97) [0·0091] | 0·94 (0·84,1·05) [0·28] |  |
|  | 35-39 | 24 to <32 weeks | 2·50 (1·09,5·71) [0·03] | 2·50 (1·09,5·71) [0·03] | 2·17 (0·88,5·38) [0·093] |  |
|  |  | 32 to 36 weeks | 1·25 (0·77,2·04) [0·36] | 1·25 (0·77,2·04) [0·36] | 1·29 (0·77,2·15) [0·34] |  |
|  |  | >36 weeks | 0·83 (0·73,0·95) [0·0072] | 0·83 (0·73,0·95) [0·0072] | 0·96 (0·81,1·13) [0·63] |  |
|  | 40-45 | 24 to <32 weeks | 0·63 (0·16,2·52) [0·51] | 0·63 (0·16,2·52) [0·51] | 1·02 (0·25,4·1) [0·98] |  |
|  |  | 32 to 36 weeks | 0·84 (0·34,2·06) [0·7] | 0·84 (0·34,2·06) [0·7] | 0·98 (0·4,2·4) [0·96] |  |
|  |  | >36 weeks | 0·99 (0·75,1·32) [0·95] | 0·99 (0·75,1·32) [0·95] | 0·98 (0·74,1·3) [0·87] |  |
|  | Deprivation: High | 24 to <32 weeks | 1·49 (0·89,2·5) [0·13] | 1·51 (0·9,2·53) [0·12] | 1·21 (0·69,2·13) [0·51] | 0·69 |
|  |  | 32 to 36 weeks | 1·28 (0·93,1·77) [0·13] | 1·29 (0·94,1·79) [0·12] | 1·24 (0·89,1·74) [0·21] |  |
|  |  | >36 weeks | 0·94 (0·86,1·01) [0·098] | 0·95 (0·87,1·03) [0·18] | 0·97 (0·88,1·07) [0·49] |  |
|  | Low | 24 to <32 weeks | 1·27 (0·8,2·02) [0·32] | 1·27 (0·8,2·03) [0·31] | 0·69 (0·42,1·13) [0·14] |  |
|  |  | 32 to 36 weeks | 1·05 (0·78,1·41) [0·76] | 1·05 (0·78,1·41) [0·76] | 0·88 (0·65,1·20) [0·43] |  |
|  |  | >36 weeks | 0·86 (0·79,0·93) [0·00028] | 0·86 (0·79,0·93) [0·00035] | 0·92 (0·84,1·01) [0·076] |  |
|  | Ethnicity: White | 24 to <32 weeks | 1·23 (0·82,1·84) [0·32] | 1·26 (0·84,1·89) [0·26] | 0·88 (0·57,1·35) [0·55] | <0·0001 |
|  |  | 32 to 36 weeks | 0·92 (0·7,1·2) [0·52] | 0·94 (0·72,1·23) [0·66] | 0·86 (0·65,1·13) [0·28] |  |
|  |  | >36 weeks | 0·84 (0·78,0·9) [<0·0001] | 0·86 (0·8,0·93) [<0·0001] | 0·87 (0·8,0·95) [0·0012] |  |
|  | Other | 24 to <32 weeks | 1·48 (0·74,2·97) [0·27] | 1·54 (0·77,3·1) [0·22] | 0·86 (0·41,1·81) [0·69] |  |
|  |  | 32 to 36 weeks | 1·72 (1·17,2·52) [0·0055] | 1·79 (1·22,2·63) [0·0029] | 1·38 (0·93,2·05) [0·11] |  |
|  |  | >36 weeks | 1·09 (0·98,1·2) [0·11] | 1·13 (1·02,1·25) [0·018] | 1·04 (0·93,1·17) [0·52] |  |
|  | Unknown | 24 to <32 weeks | 1·76 (0·34,9·06) [0·5] | 1·82 (0·35,9·41) [0·47] | 0·76 (0·15,3·94) [0·74] |  |
|  |  | 32 to 36 weeks | 1·4 (0·56,3·49) [0·48] | 1·45 (0·58,3·63) [0·43] | 0·94 (0·38,2·36) [0·9] |  |
|  |  | >36 weeks | 1·39 (1·09,1·78) [0·0073] | 1·44 (1·12,1·86) [0·0042] | 1·29 (1·01,1·65) [0·039] |  |
|  | Asian | 24 to <32 weeks | 1·21 (0·5,2·92) [0·67] | 1·25 (0·52,3·01) [0·62] | 0·71 (0·27,1·83) [0·47] |  |
|  |  | 32 to 36 weeks | 1·44 (0·95,2·2) [0·089] | 1·49 (0·97,2·27) [0·066] | 1·16 (0·74,1·81) [0·51] |  |
|  |  | >36 weeks | 1·07 (0·96,1·2) [0·24] | 1·1 (0·98,1·24) [0·1] | 1 (0·87,1·14) [0·96] |  |
|  | Black | 24 to <32 weeks | 4·03 (1·18,13·78) [0·026] | 4·22 (1·23,14·42) [0·022] | 2·25 (0·6,8·39) [0·23] |  |
|  |  | 32 to 36 weeks | 2·39 (0·77,7·42) [0·13] | 2·50 (0·81,7·77) [0·11] | 1·97 (0·63,6·22) [0·25] |  |
|  |  | >36 weeks | 0·83 (0·62,1·12) [0·22] | 0·87 (0·65,1·17) [0·35] | 0·85 (0·62,1·17) [0·32] |  |
|  | SARS-CoV2 before vaccination | 24 to <32 weeks | 1·22 (0·2,7·28) [0·83] | 1·26 (0·21,7·52) [0·8] | 1·01 (0·15,6·67) [0·99] | 0·76 |
|  |  | 32 to 36 weeks | 2·21 (0·95,5·11) [0·065] | 2·28 (0·98,5·28) [0·055] | 2·39 (0·99,5·78) [0·053] |  |
|  |  | >36 weeks | 0·82 (0·65,1·03) [0·091] | 0·85 (0·67,1·07) [0·16] | 0·93 (0·72,1·22) [0·61] |  |
|  | no SARS-CoV2 before vaccination | 24 to <32 weeks | 1·29 (0·91,1·83) [0·15] | 1·31 (0·92,1·85) [0·13] | 0·88 (0·61,1·28) [0·5] |  |
|  |  | 32 to 36 weeks | 1·02 (0·82,1·27) [0·87] | 1·03 (0·83,1·29) [0·79] | 0·95 (0·75,1·19) [0·64] |  |
|  |  | >36 weeks | 0·86 (0·81,0·91) [<0·0001] | 0·87 (0·82,0·92) [<0·0001] | 0·94 (0·88,1·01) [0·07] |  |
|  | Previous pregnancies | 24 to <32 weeks | 1·04 (0·66,1·62) [0·88] | 1·04 (0·67,1·64) [0·85] | 0·69 (0·42,1·11) [0·13] | 0·40 |
|  |  | 32 to 36 weeks | 1·17 (0·9,1·52) [0·25] | 1·18 (0·90,1·53) [0·23] | 1·12 (0·85,1·47) [0·43] |  |
|  |  | >36 weeks | 0·85 (0·79,0·92) [<0·0001] | 0·86 (0·79,0·92) [<0·0001] | 0·94 (0·86,1·03) [0·17] |  |
|  | No previous pregnancies | 24 to <32 weeks | 1·75 (1·04,2·96) [0·036] | 1·75 (1·04,2·96) [0·036] | 1·18 (0·67,2·09) [0·57] |  |
|  |  | 32 to 36 weeks | 0·92 (0·64,1·32) [0·64] | 0·92 (0·63,1·32) [0·64] | 0·81 (0·56,1·19) [0·28] |  |
|  |  | >36 weeks | 0·87 (0·8,0·95) [0·0019] | 0·87 (0·8,0·95) [0·002] | 0·93 (0·84,1·03) [0·14] |  |
|  | Singleton pregnancy | 24 to <32 weeks | 1·28 (0·91,1·79) [0·16] | 1·29 (0·92,1·82) [0·14] | 0·87 (0·61,1·26) [0·46] |  |
|  |  | 32 to 36 weeks | 1·07 (0·86,1·32) [0·55] | 1·08 (0·87,1·34) [0·47] | 1·00 (0·8,1·25) [0·99] |  |
|  |  | >36 weeks | 0·85 (0·8,0·9) [<0·0001] | 0·86 (0·81,0·91) [<0·0001] | 0·94 (0·88,1) [0·067] |  |
|  | JCVI gp:4 (vulnerable) | 24 to <32 weeks | 0·71 (0·1,5·05) [0·73] | 0·71 (0·1,5·02) [0·73] | 0·29 (0·04,2·25) [0·24] | 0·14 |
|  |  | 32 to 36 weeks | 0·6 (0·18,1·96) [0·39] | 0·59 (0·18,1·95) [0·39] | 0·59 (0·18,1·94) [0·39] |  |
|  |  | >36 weeks | 0·94 (0·63,1·42) [0·78] | 0·94 (0·62,1·42) [0·76] | 1·41 (0·93,2·13) [0·1] |  |
|  | JCVI gp:10 (age>=40) | 24 to <32 weeks | 0·6 (0·16,2·34) [0·46] | 0·61 (0·16,2·34) [0·47] | 1·16 (0·3,4·51) [0·83] |  |
|  |  | 32 to 36 weeks | 0·77 (0·28,2·08) [0·6] | 0·77 (0·29,2·08) [0·61] | 0·99 (0·37,2·69) [0·99] |  |
|  |  | >36 weeks | 0·97 (0·75,1·27) [0·84] | 0·97 (0·75,1·27) [0·85] | 1 (0·77,1·31) [1] |  |
|  | JCVI gp:11 (age>=30) | 24 to <32 weeks | 1·44 (0·86,2·44) [0·17] | 1·43 (0·85,2·41) [0·18] | 0·98 (0·56,1·72) [0·95] |  |
|  |  | 32 to 36 weeks | 1·23 (0·9,1·67) [0·19] | 1·22 (0·9,1·65) [0·21] | 1·11 (0·8,1·52) [0·54] |  |
|  |  | >36 weeks | 0·88 (0·81,0·95) [0·0012] | 0·87 (0·8,0·94) [5e-04] | 0·94 (0·86,1·04) [0·23] |  |
|  | JCVI gp:12 (age>=18) | 24 to <32 weeks | 1·38 (0·83,2·3) [0·21] | 1·42 (0·85,2·35) [0·18] | 0·91 (0·53,1·57) [0·74] |  |
|  |  | 32 to 36 weeks | 1 (0·71,1·41) [0·99] | 1·03 (0·73,1·44) [0·89] | 0·96 (0·68,1·37) [0·83] |  |
|  |  | >36 weeks | 0·81 (0·74,0·89) [<0·0001] | 0·83 (0·76,0·92) [0·00013] | 0·91 (0·83,1·01) [0·082] |  |
| **Stillbirth** | First dose before pregnancy (PRIMARY ANALYSIS) | | 0·84 (0·68,1·04) [0·11] | 0·78 (0·63,0·97) [0·023] | 0·86 (0·67,1·1) [0·22] |  |
|  | Age:18-24 |  | 0·99 (0·56,1·75) [0·97] | 0·99 (0·56,1·75) [0·97] | 1·09 (0·59,2·02) [0·79] | 0·43 |
|  | 25-29 |  | 0·91 (0·59,1·39) [0·65] | 0·91 (0·59,1·39) [0·65] | 0·96 (0·59,1·58) [0·88] |  |
|  | 30-34 |  | 0·76 (0·52,1·12) [0·17] | 0·76 (0·52,1·12) [0·17] | 0·76 (0·48,1·2) [0·24] |  |
|  | 35-39 |  | 0·76 (0·49,1·19) [0·23] | 0·76 (0·49,1·19) [0·23] | 0·99 (0·57,1·7) [0·96] |  |
|  | 40-45 |  | 0·44 (0·2,0·97) [0·042] | 0·44 (0·2,0·97) [0·042] | 0·74 (0·28,1·97) [0·54] |  |
|  | Deprivation: High |  | 0·87 (0·64,1·16) [0·34] | 0·79 (0·58,1·07) [0·12] | 0·9 (0·63,1·29) [0·56] | 0·72 |
|  | Low |  | 0·88 (0·65,1·19) [0·41] | 0·83 (0·61,1·12) [0·22] | 0·83 (0·58,1·17) [0·28] |  |
|  | Ethnicity: White |  | 0·97 (0·76,1·24) [0·81] | 0·91 (0·71,1·16) [0·44] | 0·96 (0·71,1·29) [0·77] | 0·19 |
|  | Other |  | 0·64 (0·39,1·05) [0·076] | 0·6 (0·36,0·99) [0·045] | 0·61 (0·35,1·07) [0·086] |  |
|  | Unknown |  | 0·76 (0·27,2·19) [0·61] | 0·62 (0·21,1·81) [0·38] | 0·61 (0·18,2·06) [0·43] |  |
|  | Asian |  | 0·85 (0·48,1·49) [0·56] | 0·8 (0·45,1·42) [0·44] | 0·87 (0·44,1·72) [0·68] |  |
|  | Black |  | 0·16 (0·02,1·17) [0·071] | 0·15 (0·02,1·1) [0·063] | 0·13 (0·02,0·99) [0·049] |  |
|  | SARS-CoV2 before vaccination | | 1·09 (0·46,2·57) [0·85] | 1·07 (0·45,2·55) [0·88] | 0·79 (0·3,2·1) [0·63] | 0·017 |
|  | no SARS-CoV2 before vaccination | | 0·83 (0·67,1·04) [0·1] | 0·77 (0·62,0·96) [0·021] | 0·86 (0·66,1·11) [0·24] |  |
|  | Previous pregnancies | | 0·87 (0·68,1·13) [0·3] | 0·79 (0·61,1·03) [0·081] | 0·84 (0·62,1·14) [0·25] | 0·99 |
|  | No previous pregnancies | | 0·78 (0·54,1·12) [0·18] | 0·75 (0·52,1·1) [0·14] | 0·91 (0·59,1·41) [0·68] |  |
|  | Multiple pregnancy | | 1·82 (0·94,3·54) [0·078] | 1·78 (0·9,3·51) [0·097] | 2·18 (0·9,5·3) [0·085] | <0·0001 |
|  | Singleton pregnancy | | 0·79 (0·63,0·98) [0·033] | 0·73 (0·58,0·91) [0·0056] | 0·8 (0·61,1·04) [0·095] |  |
|  | JCVI gp:10 (age>=40) | | 0·47 (0·21,1·04) [0·061] | 0·46 (0·21,1·02) [0·054] | 0·76 (0·28,2·02) [0·58] | 0·46 |
|  | JCVI gp:11 (age>=30) | | 0·81 (0·61,1·08) [0·15] | 0·78 (0·58,1·04) [0·089] | 0·84 (0·59,1·19) [0·33] |  |
|  | JCVI gp:12 (age>=18) | | 0·88 (0·61,1·26) [0·48] | 0·9 (0·63,1·28) [0·55] | 0·96 (0·64,1·43) [0·83] |  |
| **Venous events** | First dose before pregnancy (PRIMARY ANALYSIS) | | 1·4 (1·1,1·77) [0·0056] | 1·41 (1·11,1·8) [0·0055] | 1·27 (0·95,1·69) [0·11] |  |
|  | Age:18-24 |  | 2·01 (1·13,3·57) [0·017] | 2·01 (1·13,3·57) [0·017] | 1·47 (0·78,2·78) [0·24] | 0·26 |
|  | 25-29 |  | 0·98 (0·6,1·6) [0·94] | 0·98 (0·6,1·6) [0·94] | 0·78 (0·45,1·37) [0·39] |  |
|  | 30-34 |  | 1·68 (1·11,2·54) [0·014] | 1·68 (1·11,2·54) [0·014] | 1·89 (1·08,3·32) [0·026] |  |
|  | 35-39 |  | 1·4 (0·8,2·44) [0·23] | 1·4 (0·8,2·44) [0·23] | 1·42 (0·81,2·47) [0·22] |  |
|  | Deprivation: High | | 1·4 (0·98,2·01) [0·067] | 1·46 (1·01,2·11) [0·046] | 1·17 (0·75,1·83) [0·5] | 0·65 |
|  | Low |  | 1·56 (1·13,2·15) [0·0064] | 1·53 (1·1,2·11) [0·011] | 1·35 (0·93,1·98) [0·12] |  |
|  | Ethnicity: White | | 1·46 (1·13,1·89) [0·0041] | 1·48 (1·13,1·93) [0·004] | 1·38 (1·01,1·9) [0·045] | 0·049 |
|  | Other |  | 0·85 (0·42,1·76) [0·67] | 0·8 (0·38,1·67) [0·55] | 0·74 (0·32,1·71) [0·48] |  |
|  | Unknown |  | 0·99 (0·21,4·59) [0·99] | 1·04 (0·21,5·11) [0·96] | 0·97 (0·21,4·5) [0·97] |  |
|  | Asian |  | 0·87 (0·35,2·16) [0·77] | 0·79 (0·31,1·99) [0·61] | 0·46 (0·19,1·14) [0·094] |  |
|  | Black |  | 1·83 (0·52,6·49) [0·35] | 1·61 (0·45,5·86) [0·47] | 2·95 (0·83,10·52) [0·095] |  |
|  | SARS-CoV2 before vaccination | | 1·51 (0·75,3·07) [0·25] | 1·56 (0·76,3·18) [0·23] | 1·29 (0·56,2·98) [0·55] | 0·055 |
|  | no SARS-CoV2 before vaccination | | 1·36 (1·06,1·75) [0·016] | 1·37 (1·06,1·77) [0·018] | 1·26 (0·93,1·72) [0·14] |  |
|  | Previous pregnancies | | 1·34 (1·02,1·75) [0·033] | 1·39 (1·06,1·83) [0·019] | 1·25 (0·9,1·73) [0·18] | 0·18 |
|  | No previous pregnancies | | 1·53 (0·92,2·54) [0·10] | 1·5 (0·89,2·52) [0·13] | 1·28 (0·77,2·13) [0·34] |  |
|  | Singleton pregnancy | | 1·41 (1·11,1·8) [0·0049] | 1·42 (1·11,1·82) [0·0049] | 1·26 (0·94,1·68) [0·13] |  |
|  | JCVI gp:11 (age>=30) | | 1·52 (1·09,2·11) [0·013] | 1·5 (1·08,2·09) [0·016] | 1·62 (1·06,2·48) [0·027] | 0·62 |
|  | JCVI gp:12 (age>=18) | | 1·28 (0·87,1·88) [0·21] | 1·29 (0·88,1·91) [0·2] | 0·96 (0·62,1·49) [0·87] |  |

Supplementary Table 6: Sensitivity analyses censoring at first COVID-19 infection in pregnancy: Hazard Ratios of pregnancy outcomes following pre-pregnancy vaccination exposure among pregnancies in England with an estimated start date between 08 December 2020 and 28 August 2021

| **Outcome** | **Exposure subgroup** |  | **Events (n)** | **Hazard Ratios (95% CI) [p-value]** | | |
| --- | --- | --- | --- | --- | --- | --- |
|  |  | **Follow up time interval (gest·weeks)** |  | **Unadjusted** | **Age-adjusted** | **Maximally Adjusted** |
| Preterm birth | First dose before pregnancy – Primary analysis | 24 to <32 | 2380 | 1∙61 (1∙48, 1∙76) [<0∙0001] | 1∙57 (1∙44, 1∙72) [<0∙0001] | 0·59 (0·48,0·73) [<0·0001] |
|  |  | 32 to 36 | 6370 | 1∙46 (1∙38, 1∙55) [<0∙0001] | 1∙42 (1∙34, 1∙51) [<0∙0001] | 1·01 (0·94,1·08) [0·82] |
|  | -of which : ChAdOx1 (viral-vector vaccine) | 24 to <32 | 2055 | 1∙57 (1∙39, 1·77) [<0∙0001] | 1·53 (1·36, 1·72)[<0∙0001] | 0·67 (0·51,0·88) [0·004] |
|  |  | 32 to 36 | 5715 | 1∙62 (1∙50, 1∙74) [<0∙0001] | 1∙58 (1∙46, 1∙70) [<0∙0001] | 0·99 (0·91,1·08) [0·81] |
|  | -of which : BNT162b2 (mRNA vaccine) | 24 to <32 | 2055 | 1∙61 (1∙44, 1∙81) [<0∙0001] | 1∙57 (1∙40, 1∙77) [<0∙0001] | 0·54 (0·41,0·71) [<0·0001] |
|  |  | 32 to 36 | 5565 | 1∙30 (1∙20, 1∙41) [<0∙0001] | 1∙27 (1∙17, 1∙38) [<0∙0001] | 1·03 (0·94,1·12) [0·57] |
|  | Two dose before pregnancy | 24 to <32 | 2245 | 1∙78 (1∙61, 1∙96) [<0∙0001] | 1∙73 (1∙57, 1∙90) [<0∙0001] | 0·57 (0·45,0·72) [<0·0001] |
|  |  | 32 to 36 | 6000 | 1∙52 (1∙43, 1∙63) [<0∙0001] | 1∙48 (1∙39, 1∙58) [<0∙0001] | 0·98 (0·91,1·06) [0·64] |
|  | Three doses by 24 weeks gestation | 24 to <32 | 1802 | 12∙43 (9∙66, 16∙01) [<0∙0001] | 11∙97(9∙29,15∙41)[<0∙0001] | 0·51 (0·29,0·88) [0·016] |
|  |  | 32 to 36 | 4955 | 3∙56 (2∙59, 4∙90) [<0∙0001] | 3∙43 (2∙49, 4∙72) [<0∙0001] | 1·09 (0·78,1·51) [0·62] |
|  | First dose before pregnancy (complete gestational age) | 24 to <32 | 2080 | 1∙54 (1∙40, 1·69) [<0∙0001] | 1∙50 (1·36,1·65) [<0∙0001] | 0·59 (0·47,0·74) [<0·0001] |
|  |  | 32 to 36 | 5590 | 1∙40 (1∙32, 1∙49) [<0∙0001] | 1∙37 (1∙29, 1∙46) [<0∙0001] | 1 (0·94,1·08) [0·91] |
| Small for gestational age | First dose before pregnancy – Primary analysis | 24 to <32 | 110 | 0·8 (0·55,1·16) [0·24] | 0·81 (0·56,1·17) [0·26] | 0·62 (0·41,0·94) [0·023] |
|  |  | 32 to 36 | 275 | 1·05 (0·78,1·41) [0·76] | 1·06 (0·79,1·42) [0·71] | 1·04 (0·77,1·41) [0·79] |
|  |  | >36 | 4480 | 0·9 (0·83,0·98) [0·02] | 0·91 (0·84,0·99) [0·036] | 0·96 (0·87,1·05) [0·37] |
|  | -of which : ChAdOx1 (viral-vector vaccine) | 24 to <32 | 100 | 0·85 (0·52,1·39) [0·52] | 0·86 (0·52,1·41) [0·54] | 0·7 (0·41,1·18) [0·18] |
|  |  | 32 to 36 | 250 | 1·19 (0·82,1·74) [0·37] | 1·2 (0·82,1·75) [0·35] | 1·1 (0·75,1·61) [0·64] |
|  |  | >36 | 4150 | 0·91 (0·81,1·02) [0·12] | 0·92 (0·82,1·03) [0·15] | 0·9 (0·79,1·02) [0·099] |
|  | -of which : BNT162b2 (mRNA vaccine) | 24 to <32 | 90 | 0·75 (0·44,1·26) [0·28] | 0·76 (0·45,1·28) [0·3] | 0·54 (0·3,0·97) [0·041] |
|  |  | 32 to 36 | 245 | 0·91 (0·59,1·39) [0·65] | 0·92 (0·6,1·41) [0·7] | 0·99 (0·64,1·51) [0·94] |
|  |  | >36 | 4180 | 0·9 (0·8,1·01) [0·067] | 0·91 (0·82,1·02) [0·12] | 1 (0·89,1·13) [1] |
|  | Two dose before pregnancy | 24 to <32 | 105 | 0·87 (0·57,1·32) [0·51] | 0·88 (0·58,1·34) [0·56] | 0·62 (0·39,0·99) [0·045] |
|  |  | 32 to 36 | 255 | 0·94 (0·66,1·35) [0·74] | 0·95 (0·67,1·36) [0·79] | 0·92 (0·64,1·32) [0·63] |
|  |  | >36 | 4285 | 0·87 (0·79,0·96) [0·0057] | 0·88 (0·8,0·97) [0·013] | 0·92 (0·82,1·02) [0·11] |
|  | Three doses by 24 weeks gestation | 24 to <32 | 80 | 2·01 (0·28,14·33) [0·49] | 2·05 (0·29,14·62) [0·47] | 0·1 (0·01,0·85) [0·035] |
|  |  | 32 to 36 | 225 | 3·65 (0·91,14·67) [0·068] | 3·72 (0·93,14·96) [0·064] | 2·56 (0·63,10·37) [0·19] |
|  |  | >36 | 3860 | 1·15 (0·52,2·56) [0·74] | 1·17 (0·53,2·61) [0·7] | 0·96 (0·43,2·15) [0·92] |
|  | First dose before pregnancy (complete gestational age) | 24 to <32 | 110 | 0·78 (0·54,1·13) [0·19] | 0·78 (0·54,1·14) [0·2] | 0·63 (0·42,0·96) [0·031] |
|  |  | 32 to 36 | 275 | 1·02 (0·76,1·38) [0·88] | 1·03 (0·76,1·38) [0·86] | 1·05 (0·77,1·41) [0·77] |
|  |  | >36 | 4475 | 0·91 (0·84,0·99) [0·031] | 0·91 (0·84,1) [0·038] | 0·98 (0·89,1·08) [0·68] |
| Stillbirth | First dose before pregnancy – Primary analysis |  | 365 | 0·92 (0·68,1·23) [0·55] | 0·85 (0·64,1·15) [0·29] | 0·81 (0·59,1·13) [0·22] |
|  | -of which : ChAdOx1 (viral-vector vaccine) |  | 370 | 1·11 (0·76,1·61) [0·6] | 1·04 (0·71,1·51) [0·85] | 0·94 (0·62,1·42) [0·76] |
|  | -of which : BNT162b2 (mRNA vaccine) |  | 335 | 0·74 (0·48,1·14) [0·17] | 0·69 (0·45,1·06) [0·089] | 0·71 (0·45,1·12) [0·14] |
|  | Two dose before pregnancy |  | 355 | 1·01 (0·73,1·39) [0·98] | 0·92 (0·67,1·28) [0·63] | 0·87 (0·6,1·25) [0·44] |
|  | Three doses by 24 weeks gestation |  | 315 | 2·28 (0·32,16·22) [0·41] | 2·02 (0·28,14·43) [0·48] | 1·34 (0·16,11·2) [0·79] |
|  | First dose before pregnancy (complete gestational age) |  | 200 | 0·9 (0·61,1·33) [0·59] | 0·84 (0·57,1·25) [0·39] | 0·68 (0·44,1·05) [0·078] |
| Venous events | First dose before pregnancy – Primary analysis |  | 205 | 1·42 (1·02,1·97) [0·04] | 1·42 (1·02,1·99) [0·039] | 1·16 (0·8,1·71) [0·44] |
|  | -of which : ChAdOx1 (viral-vector vaccine) |  | 185 | 1·59 (1·04,2·43) [0·031] | 1·6 (1·05,2·45) [0·03] | 1·23 (0·76,1·98) [0·41] |
|  | -of which : BNT162b2 (mRNA vaccine) |  | 180 | 1·25 (0·78,1·99) [0·35] | 1·23 (0·77,1·97) [0·38] | 1·06 (0·65,1·75) [0·81] |
|  | Two dose before pregnancy |  | 195 | 1·5 (1·03,2·17) [0·033] | 1·5 (1·03,2·18) [0·036] | 1·29 (0·84,1·98) [0·25] |
|  | Three doses by 24 weeks gestation |  | 160 | 5·29 (1·31,21·35) [0·019] | 5·21 (1·29,21·09) [0·021] | 3·71 (0·82,16·75) [0·088] |
|  | First dose before pregnancy (complete gestational age) |  | 150 | 1·39 (0·94,2·05) [0·096] | 1·4 (0·95,2·08) [0·089] | 1·17 (0·75,1·83) [0·49] |

Supplementary Table 7: Proportional Assumption test results for the Cox proportional model for four outcomes

| **Outcome** | **Variable** | **Chisq.** | **df** | **Pr(<Chisq)** |
| --- | --- | --- | --- | --- |
| Small for gestational age | Week 24 to <32 | 0·017853 | 1 | 0·89 |
|  | Week 32 to <36 | 0·967799 | 1 | 0·33 |
|  | Week 36 & above | 3·122037 | 1 | 0·077 |
|  | Age | 1·855825 | 1 | 0·17 |
|  | Deprivation Q2 | 0·895723 | 1 | 0·34 |
|  | Deprivation Q3 | 1·19314 | 1 | 0·27 |
|  | Deprivation Q4 | 1·433638 | 1 | 0·23 |
|  | Deprivation Q5 | 2·392983 | 1 | 0·12 |
|  | Ethnicity -Unknown | 0·481277 | 1 | 0·49 |
|  | Ethnicity - White | 7·982438 | 1 | 0·0047 |
|  | Region East England | 0·751206 | 1 | 0·39 |
|  | Region London | 2·973344 | 1 | 0·085 |
|  | Region North East | 0·303365 | 1 | 0·58 |
|  | Region North West | 3·546962 | 1 | 0·06 |
|  | Region South East | 12·96343 | 1 | 0·0003 |
|  | Region South East | 5·157954 | 1 | 0·023 |
|  | Region West Midlands | 1·312571 | 1 | 0·25 |
|  | Region York & the Humber | 10·83143 | 1 | 0·001 |
|  | H/o Obesity | 13·06472 | 1 | 0·0003 |
|  | H/o COVID-19 | 0·393437 | 1 | 0·53 |
|  | H/o Depression anxiety | 20·64286 | 1 | <0·0001 |
|  | H/o Diabetes | 1·817841 | 1 | 0·18 |
|  | H/o Hypertensive disorder | 16·995 | 1 | <0·0001 |
|  | H/o PCOS | 1·225438 | 1 | 0·27 |
|  | H/o pregnancy | 33·94044 | 1 | <0·0001 |
|  | H/o Stillbirth | 2·867891 | 1 | 0·09 |
|  | H/o Venous event | 3·04173 | 1 | 0·081 |
|  | H/o Thrombophilia | 1·008306 | 1 | 0·32 |
|  | H/o Chronic Kidney disease | 0·033807 | 1 | 0·85 |
|  | H/o Lipid lowering drugs | 1·360844 | 1 | 0·24 |
|  | H/o Anticoagulant drugs | 1·522565 | 1 | 0·22 |
|  | H/o Antiplatelet drugs | 0·00072 | 1 | 0·98 |
|  | H/o COCP drugs | 0·094558 | 1 | 0·76 |
|  | Current smoking | 48·59202 | 1 | <0·0001 |
|  | Ex smoker | 1·333783 | 1 | 0·25 |
|  | Never smoker | 26·27684 | 1 | <0·0001 |
|  | JCVI group 10 | 0·774002 | 1 | 0·38 |
|  | JCVI group 11 | 0·047317 | 1 | 0·83 |
|  | JCVI group 12 | 1·209556 | 1 | 0·27 |
|  | H/o surgery in last year | 3·556567 | 1 | 0·059 |
|  | Calendar months (knot1) | 1·002827 | 1 | 0·32 |
|  | Calendar months (knot2) | 10·09313 | 1 | 0·0015 |
|  | Calendar months (knot3) | 29·64334 | 1 | <0·0001 |
|  | GLOBAL | 220·7455 | 43 | <0·0001 |
| Preterm | Age | 0·426049 | 1 | 0·51 |
|  | Deprivation Q2 | 6·939138 | 1 | 0·0084 |
|  | Deprivation Q3 | 4·257279 | 1 | 0·039 |
|  | Deprivation Q4 | 4·536905 | 1 | 0·033 |
|  | Deprivation Q5 | 0·005156 | 1 | 0·94 |
|  | Ethnicity -Unknown | 1·848602 | 1 | 0·17 |
|  | Ethnicity - White | 13·20269 | 1 | 0·0003 |
|  | Region East England | 0·124875 | 1 | 0·72 |
|  | Region London | 7·544179 | 1 | 0·006 |
|  | Region North East | 0·08061 | 1 | 0·78 |
|  | Region North West | 1·22637 | 1 | 0·27 |
|  | Region South East | 3·040233 | 1 | 0·081 |
|  | Region South East | 2·180257 | 1 | 0·14 |
|  | Region West Midlands | 2·299971 | 1 | 0·13 |
|  | Region York & the Humber | 0·002344 | 1 | 0·96 |
|  | H/o Obesity | 6·169667 | 1 | 0·013 |
|  | H/o COVID-19 | 8·995212 | 1 | 0·0027 |
|  | H/o Depression anxiety | 2·56972 | 1 | 0·11 |
|  | H/o Diabetes | 15·25271 | 1 | 0·0001 |
|  | H/o Hypertensive disorder | 1·190106 | 1 | 0·28 |
|  | H/o PCOS | 0·447574 | 1 | 0·5 |
|  | H/o pregnancy | 9·595693 | 1 | 0·002 |
|  | H/o Stillbirth | 5·048749 | 1 | 0·025 |
|  | H/o Venous event | 5·158353 | 1 | 0·023 |
|  | H/o Thrombophilia | 0·50559 | 1 | 0·48 |
|  | H/o Chronic Kidney disease | 0·725059 | 1 | 0·39 |
|  | H/o Lipid lowering drugs | 1·13194 | 1 | 0·29 |
|  | H/o Anticoagulant drugs | 4·387327 | 1 | 0·036 |
|  | H/o Antiplatelet drugs | 0·124718 | 1 | 0·72 |
|  | H/o COCP drugs | 0·550078 | 1 | 0·46 |
|  | Current smoking | 0·186116 | 1 | 0·67 |
|  | Ex smoker | 3·930308 | 1 | 0·047 |
|  | Never smoker | 3·829296 | 1 | 0·05 |
|  | JCVI group 10 | 1·140414 | 1 | 0·29 |
|  | JCVI group 11 | 5·146377 | 1 | 0·023 |
|  | JCVI group 12 | 2·235267 | 1 | 0·13 |
|  | H/o surgery in last year | 0·249844 | 1 | 0·62 |
|  | Calendar months (knot1) | 2·541805 | 1 | 0·11 |
|  | Calendar months (knot2) | 11·73749 | 1 | 0·0006 |
|  | Calendar months (knot3) | 30·25589 | 1 | <0·0001 |
|  | Week 24 to <32 | 11·19757 | 1 | 0·0008 |
|  | Week 32 to <36 | 0·614023 | 1 | 0·43 |
|  | GLOBAL | 389·9036 | 42 | <0·0001 |
| Stillbirth | Vaccine exposure | 1·361288 | 1 | 0·24 |
|  | Age | 0·062526 | 1 | 0·8 |
|  | Deprivation Q2 | 0·815195 | 1 | 0·37 |
|  | Deprivation Q3 | 0·000099 | 1 | 0·99 |
|  | Deprivation Q4 | 0·024629 | 1 | 0·88 |
|  | Deprivation Q5 | 0·003118 | 1 | 0·96 |
|  | Ethnicity -Unknown | 0·840109 | 1 | 0·36 |
|  | Ethnicity - White | 3·011356 | 1 | 0·083 |
|  | Region East England | 0·012784 | 1 | 0·91 |
|  | Region London | 0·314981 | 1 | 0·57 |
|  | Region North East | 3·483169 | 1 | 0·062 |
|  | Region North West | 1·051996 | 1 | 0·3 |
|  | Region South East | 3·302553 | 1 | 0·069 |
|  | Region South East | 0·037562 | 1 | 0·85 |
|  | Region West Midlands | 2·239609 | 1 | 0·13 |
|  | Region York & the Humber | 0·41735 | 1 | 0·52 |
|  | H/o Obesity | 0·366587 | 1 | 0·54 |
|  | H/o COVID-19 | 0·224374 | 1 | 0·64 |
|  | H/o Depression anxiety | 1·923993 | 1 | 0·17 |
|  | H/o Diabetes | 0·899999 | 1 | 0·34 |
|  | H/o Hypertensive disorder | 1·420845 | 1 | 0·23 |
|  | H/o PCOS | 0·349757 | 1 | 0·55 |
|  | H/o pregnancy | 0·222825 | 1 | 0·64 |
|  | H/o Stillbirth | 0·840593 | 1 | 0·36 |
|  | H/o Venous event | 0·616863 | 1 | 0·43 |
|  | H/o Thrombophilia | 1·787605 | 1 | 0·18 |
|  | H/o Chronic Kidney disease | 0·138709 | 1 | 0·71 |
|  | H/o Lipid lowering drugs | 0·098719 | 1 | 0·75 |
|  | H/o Antiplatelet drugs | 1·220954 | 1 | 0·27 |
|  | H/o COCP drugs | 0·627241 | 1 | 0·43 |
|  | Current smoking | 0·041886 | 1 | 0·84 |
|  | Ex smoker | 0·641327 | 1 | 0·42 |
|  | Never smoker | 2·462056 | 1 | 0·12 |
|  | JCVI group 10 | 0·000936 | 1 | 0·98 |
|  | JCVI group 11 | 0·49813 | 1 | 0·48 |
|  | JCVI group 12 | 0·865217 | 1 | 0·35 |
|  | H/o surgery in last year | 1·010489 | 1 | 0·31 |
|  | Calendar months (knot1) | 0·000808 | 1 | 0·98 |
|  | Calendar months (knot2) | 2·006747 | 1 | 0·16 |
|  | Calendar months (knot3) | 2·620551 | 1 | 0·11 |
|  | GLOBAL | 44·74847 | 40 | 0·28 |
| Venous event | Vaccine exposure | 2·450411 | 1 | 0·12 |
|  | Age | 0·169599 | 1 | 0·68 |
|  | Deprivation Q2 | 0·609984 | 1 | 0·43 |
|  | Deprivation Q3 | 4·992073 | 1 | 0·026 |
|  | Deprivation Q4 | 0·243826 | 1 | 0·62 |
|  | Deprivation Q5 | 0·052357 | 1 | 0·82 |
|  | Ethnicity -Unknown | 0·11286 | 1 | 0·74 |
|  | Ethnicity - White | 1·73328 | 1 | 0·19 |
|  | Region East England | 1·414345 | 1 | 0·23 |
|  | Region London | 0·443378 | 1 | 0·51 |
|  | Region North East | 0·000567 | 1 | 0·98 |
|  | Region North West | 0·951002 | 1 | 0·33 |
|  | Region South East | 0·713814 | 1 | 0·4 |
|  | Region South East | 3·448863 | 1 | 0·063 |
|  | Region West Midlands | 1·01208 | 1 | 0·31 |
|  | Region York & the Humber | 1·112229 | 1 | 0·29 |
|  | H/o Obesity | 0·275969 | 1 | 0·6 |
|  | H/o COVID-19 | 1·6625 | 1 | 0·2 |
|  | H/o Depression anxiety | 0·398537 | 1 | 0·53 |
|  | H/o Diabetes | 0·278384 | 1 | 0·6 |
|  | H/o Hypertensive disorder | 4·095061 | 1 | 0·043 |
|  | H/o PCOS | 2·053106 | 1 | 0·15 |
|  | H/o pregnancy | 1·348721 | 1 | 0·25 |
|  | H/o Stillbirth | 1·768454 | 1 | 0·18 |
|  | H/o Venous event | 0·511185 | 1 | 0·47 |
|  | H/o Thrombophilia | 0·026771 | 1 | 0·87 |
|  | H/o Chronic Kidney disease | 0·771312 | 1 | 0·38 |
|  | H/o Anticoagulant drugs | 2·247824 | 1 | 0·13 |
|  | H/o Antiplatelet drugs | 0·024918 | 1 | 0·87 |
|  | H/o COCP drugs | 4·533576 | 1 | 0·033 |
|  | Current smoking | 1·027655 | 1 | 0·31 |
|  | Ex smoker | 1·28487 | 1 | 0·26 |
|  | Never smoker | 1·677375 | 1 | 0·2 |
|  | JCVI group 10 | 1·469888 | 1 | 0·23 |
|  | JCVI group 11 | 0·103142 | 1 | 0·75 |
|  | JCVI group 12 | 0·444806 | 1 | 0·5 |
|  | H/o surgery in last year | 2·280992 | 1 | 0·13 |
|  | Calendar months (knot1) | 1·063099 | 1 | 0·3 |
|  | Calendar months (knot2) | 0·058483 | 1 | 0·81 |
|  | Calendar months (knot3) | 0·255977 | 1 | 0·61 |
|  | GLOBAL | 54·05597 | 40 | 0·068 |

Supplementary Figure 1: Consort diagram


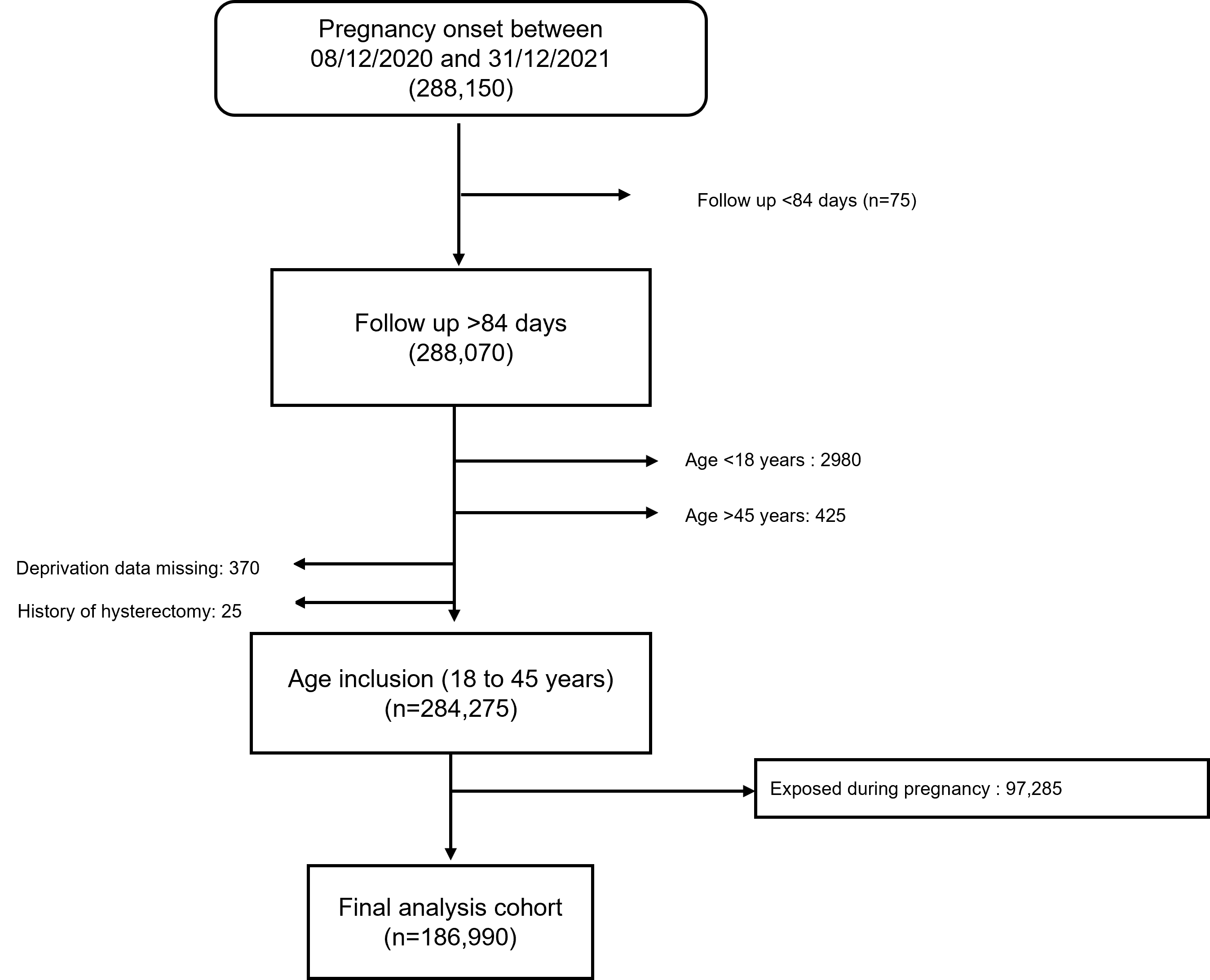


Supplementary Figure 2: Sensitivity analyses censoring at first date of COVID-19 infection in pregnancy (analysis among pregnancy started between 08 December 2020 and 28 August 2021)


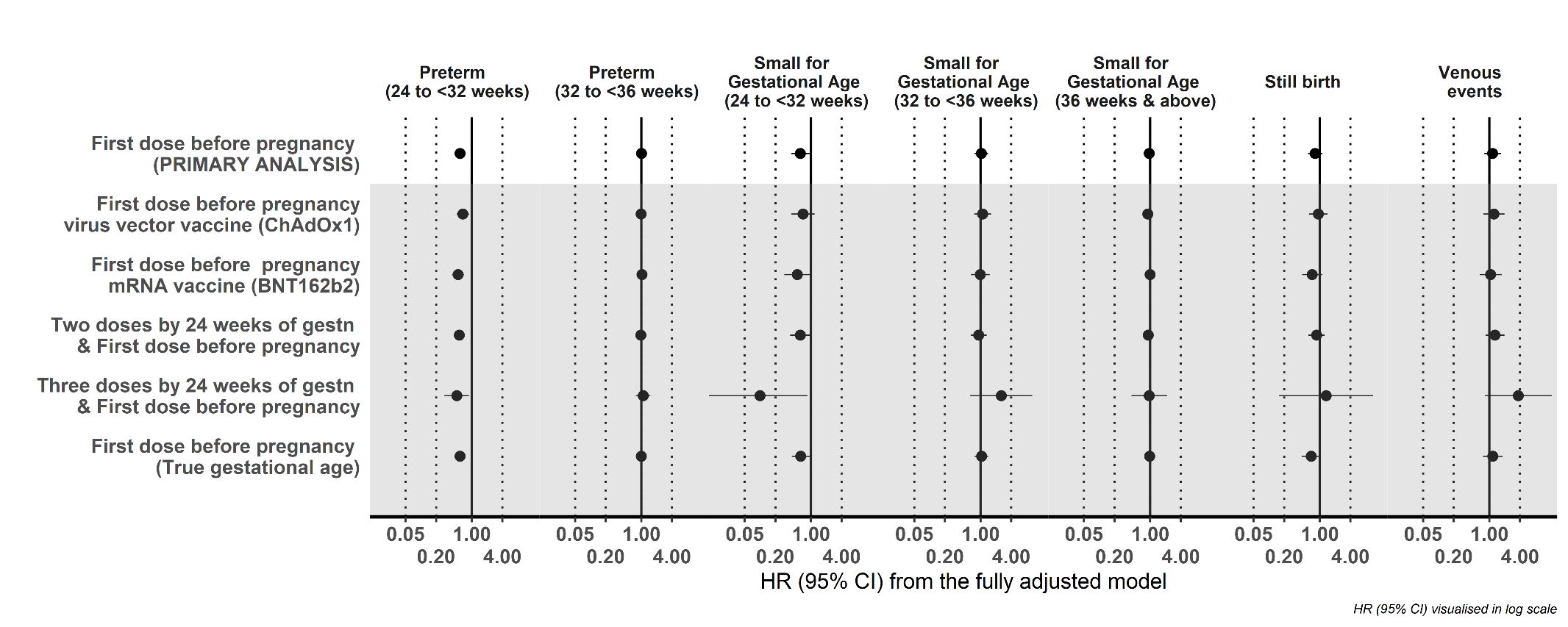


Supplementary Figure 3: Age-standardised incidence rates of venous thrombotic events during pregnancy per 100,000 person years among unvaccinated and vaccinated women.


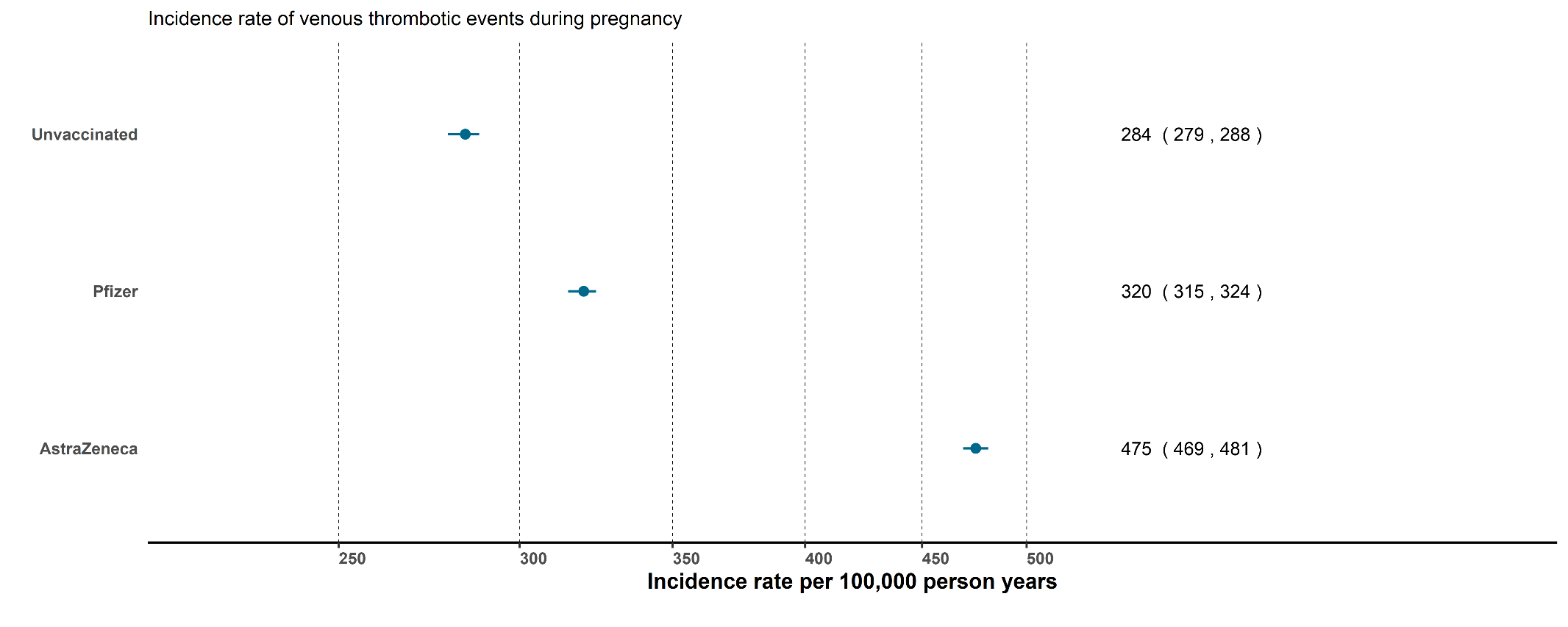


Supplementary Figure 4: Proportional Assumption results


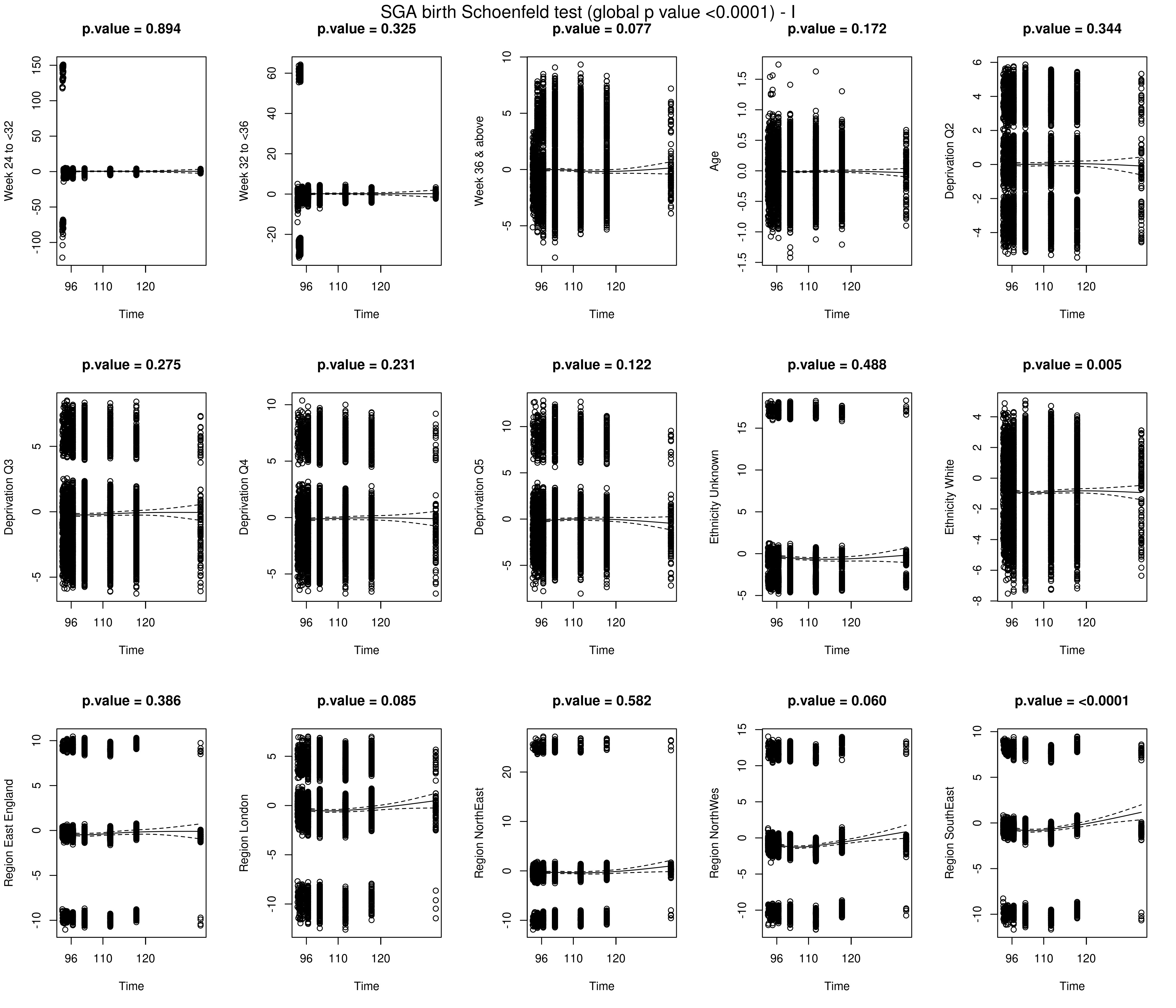


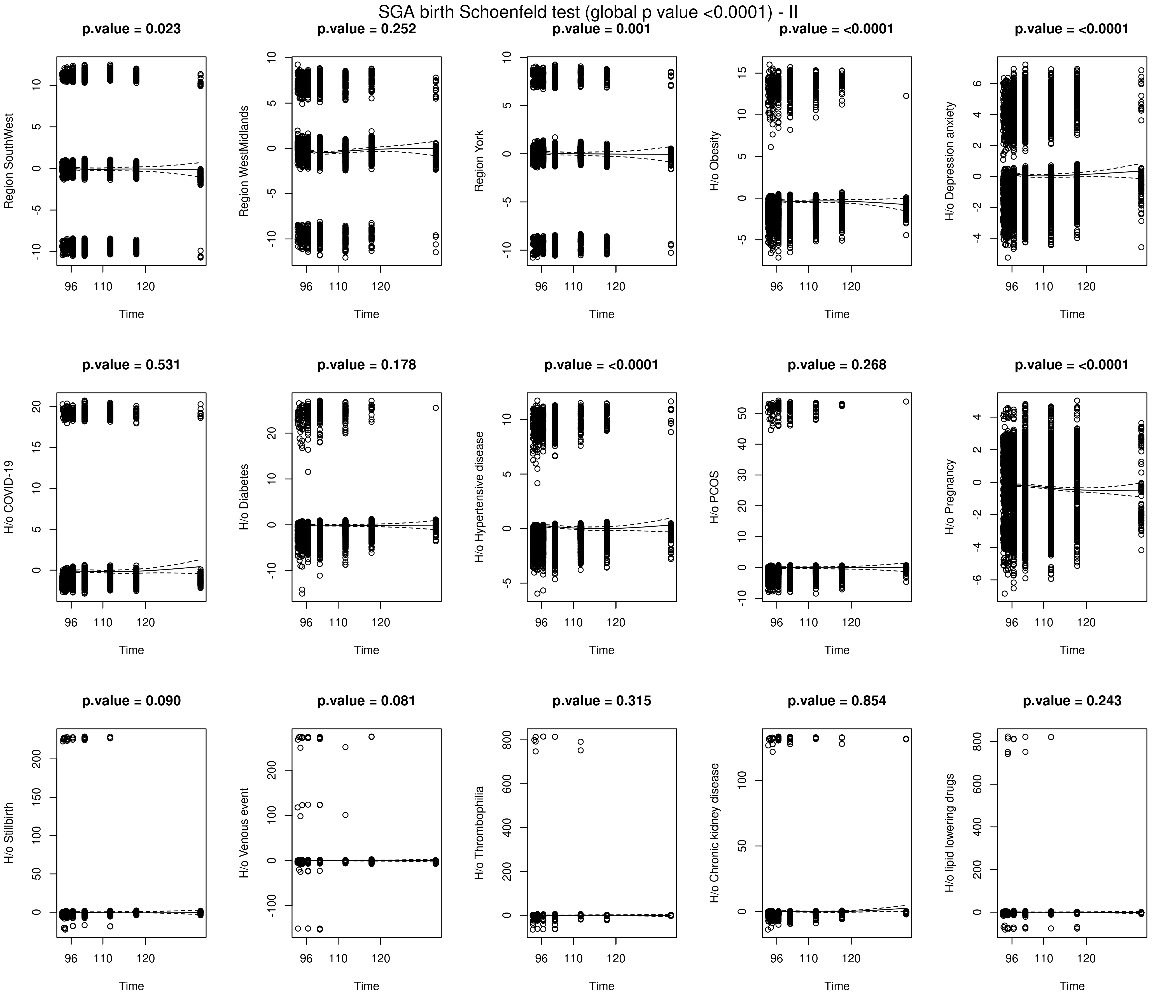


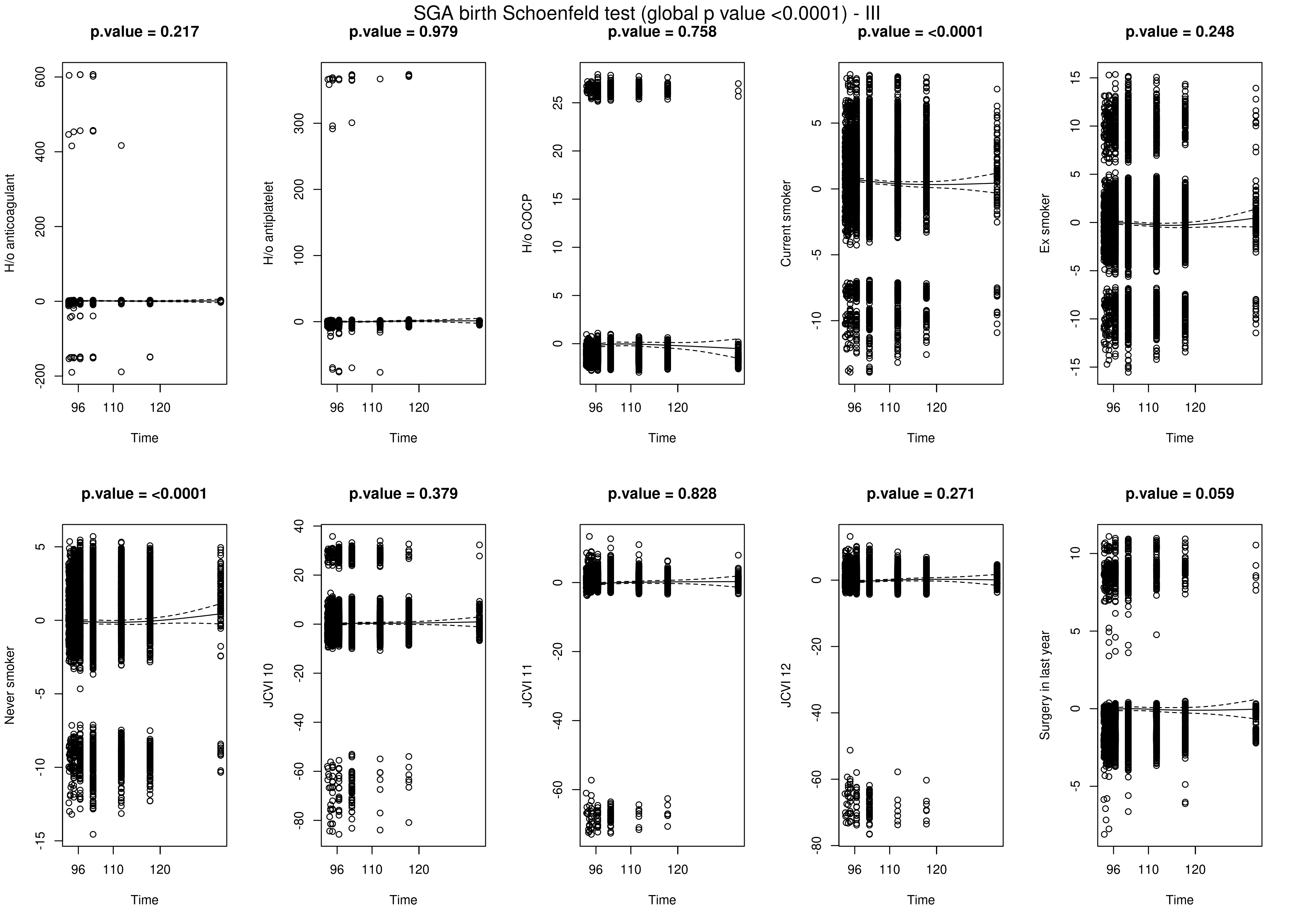


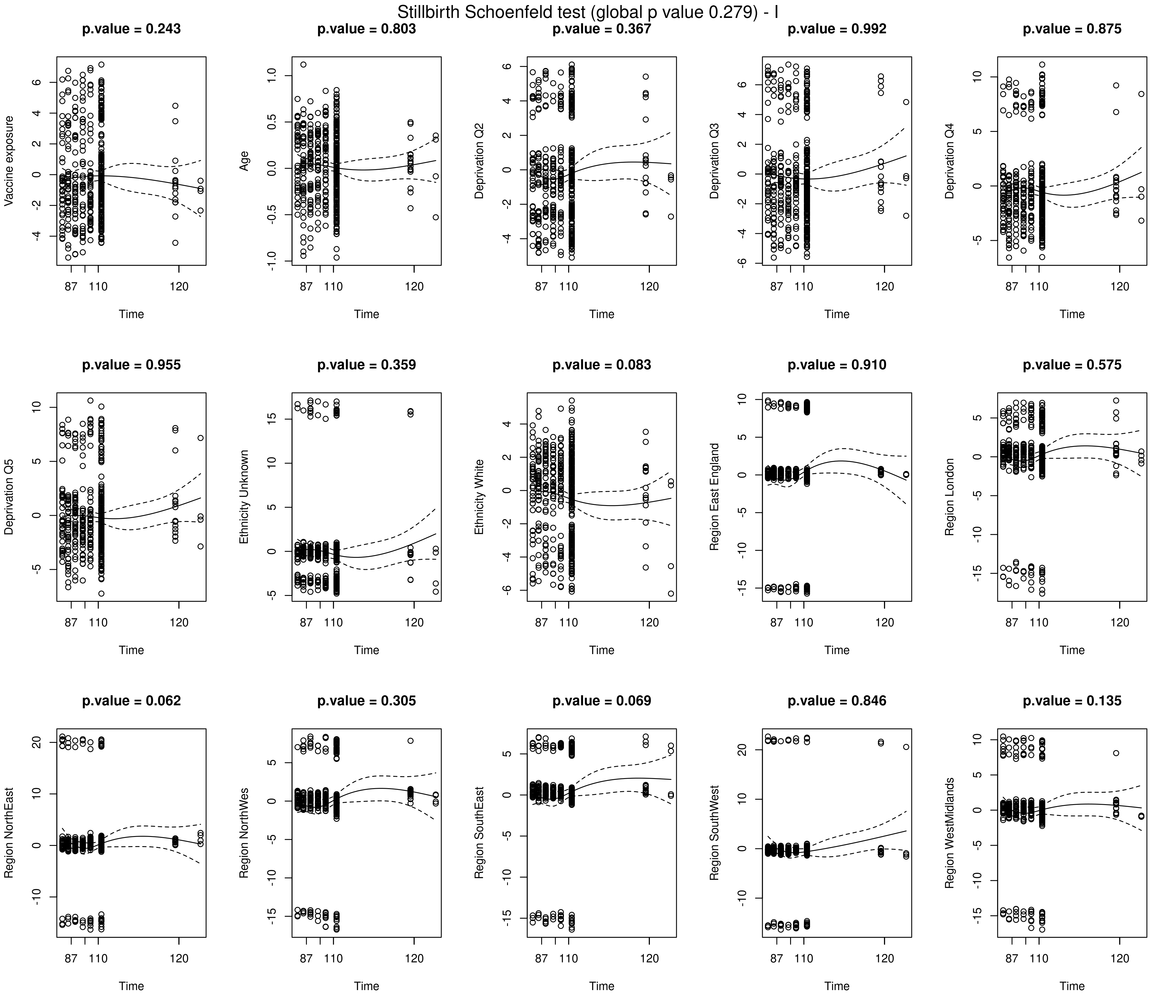


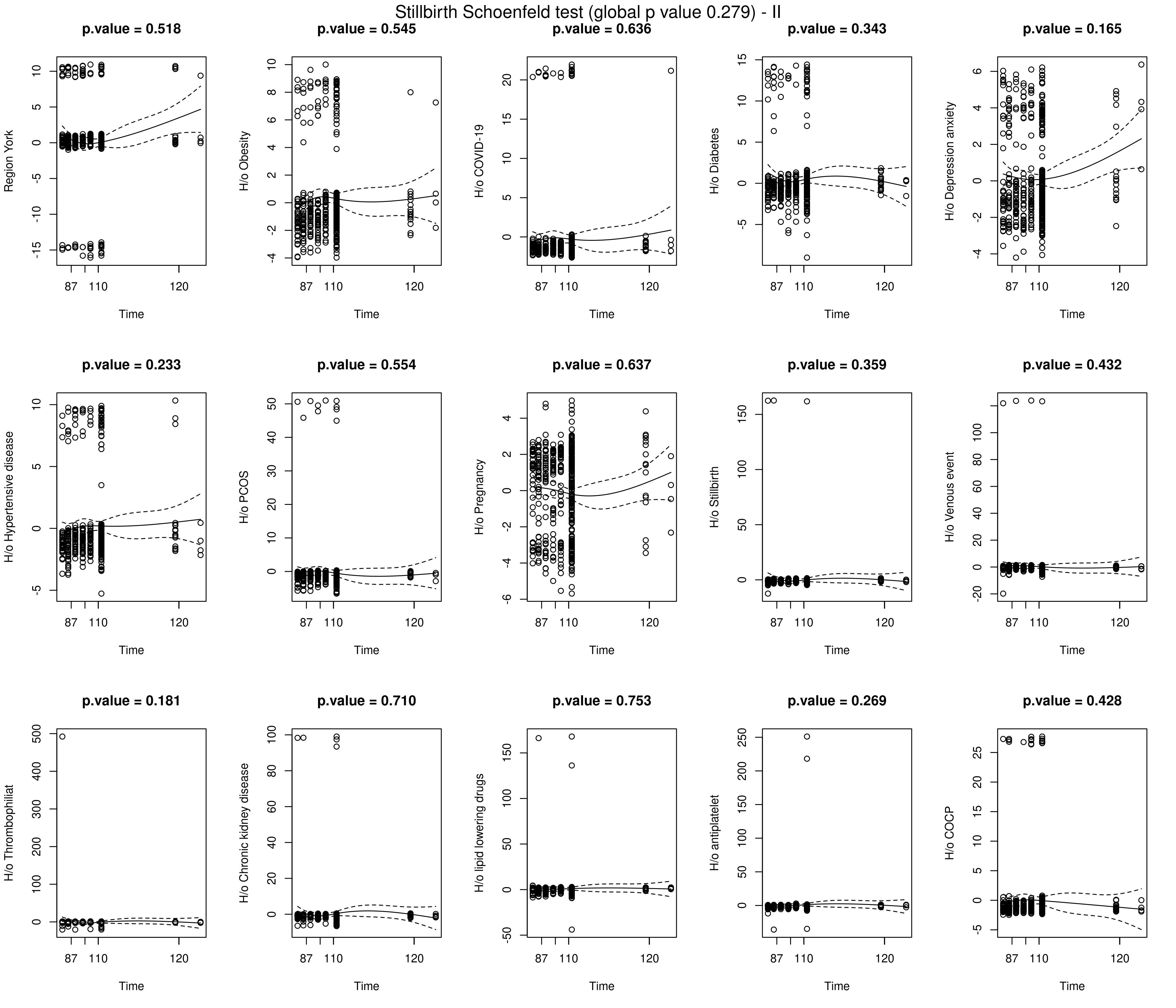


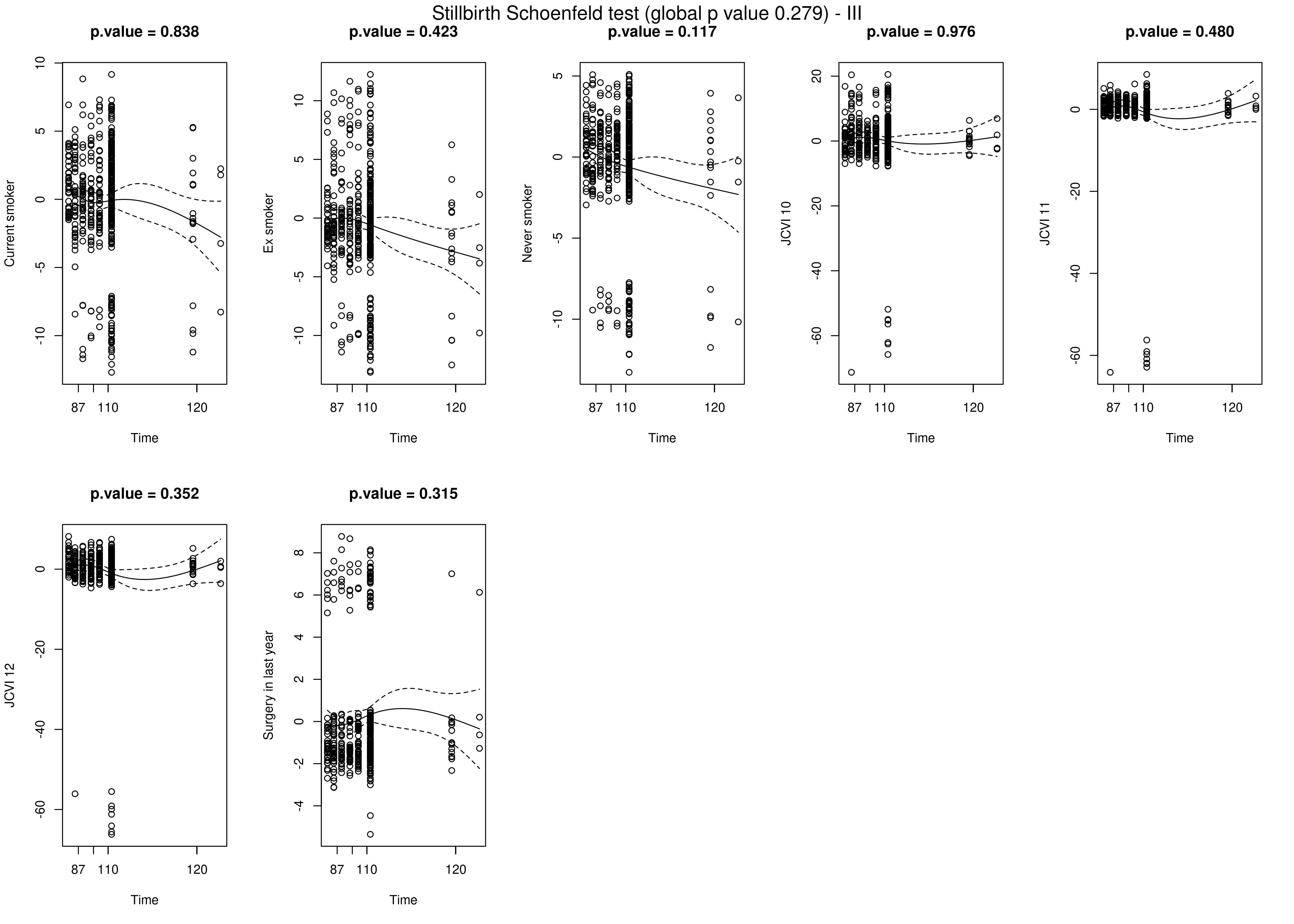


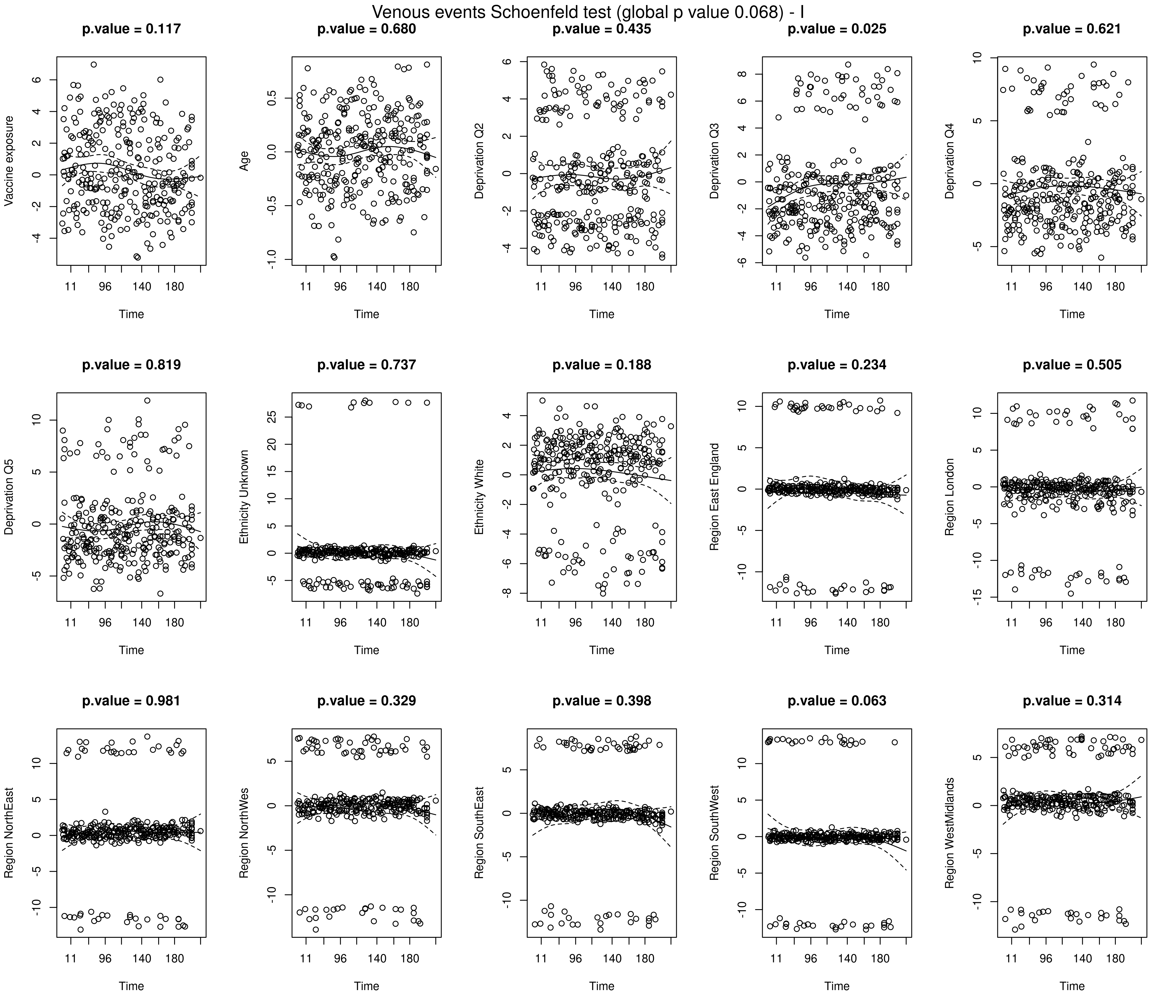


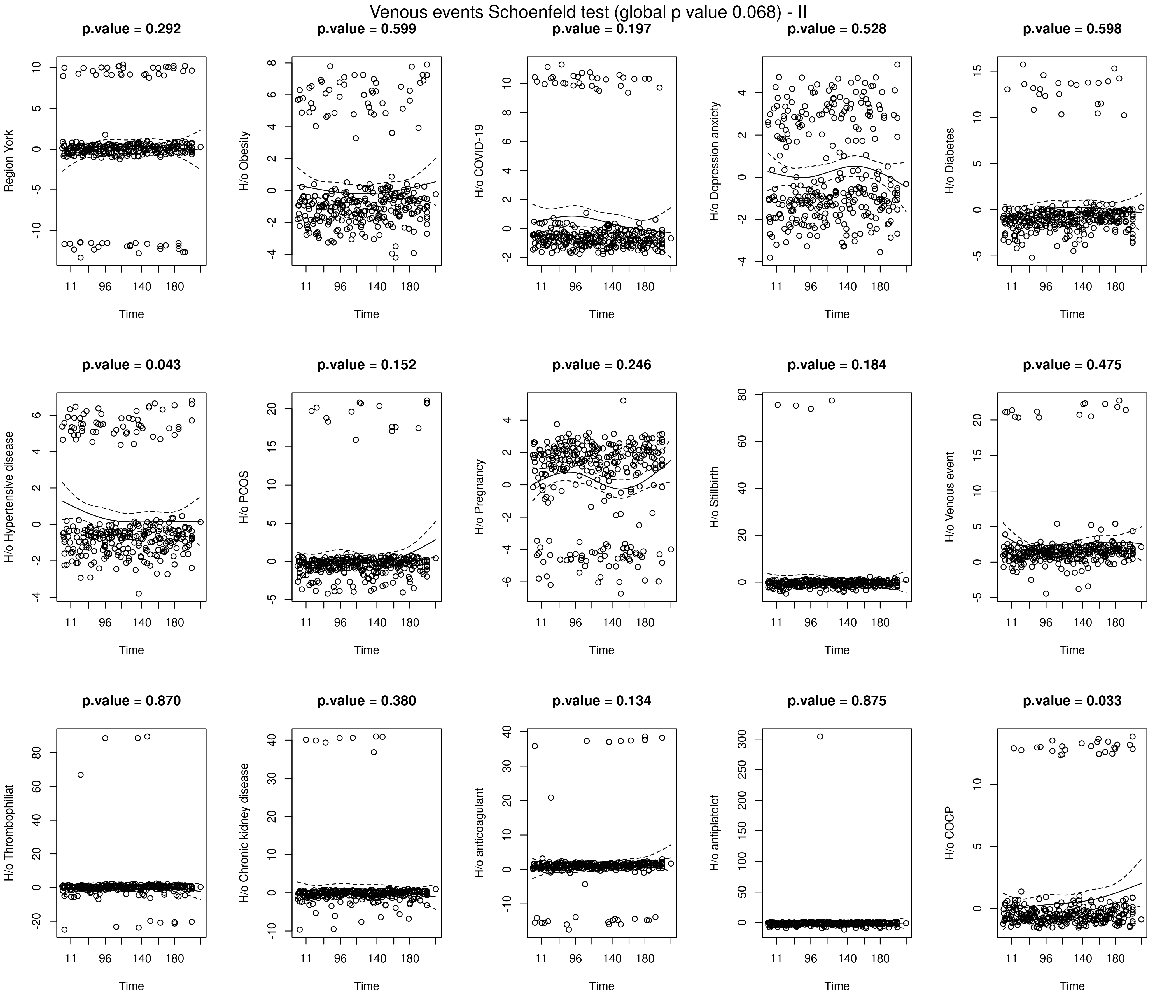


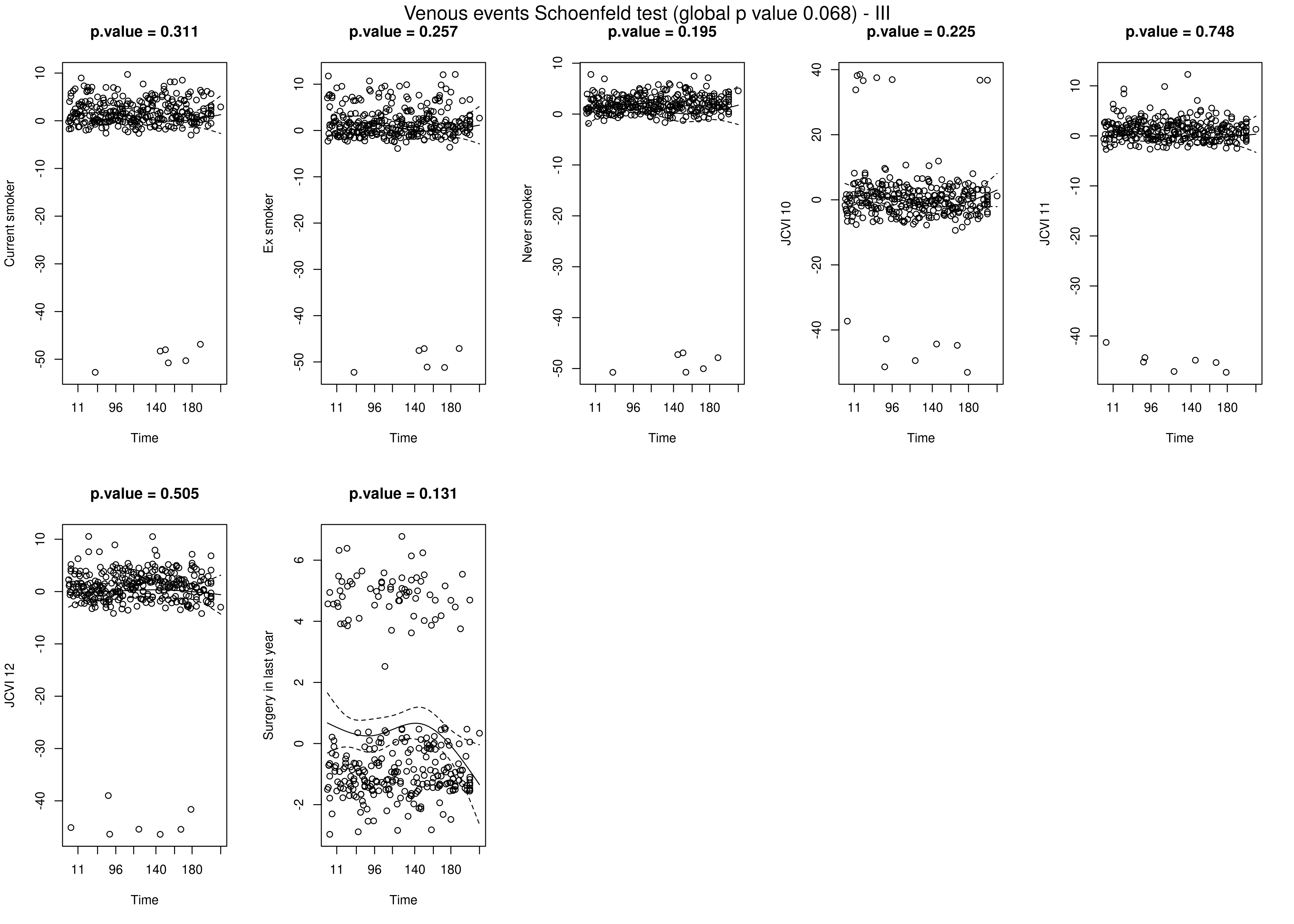


Supplementary Figure 5: Linearity assumption


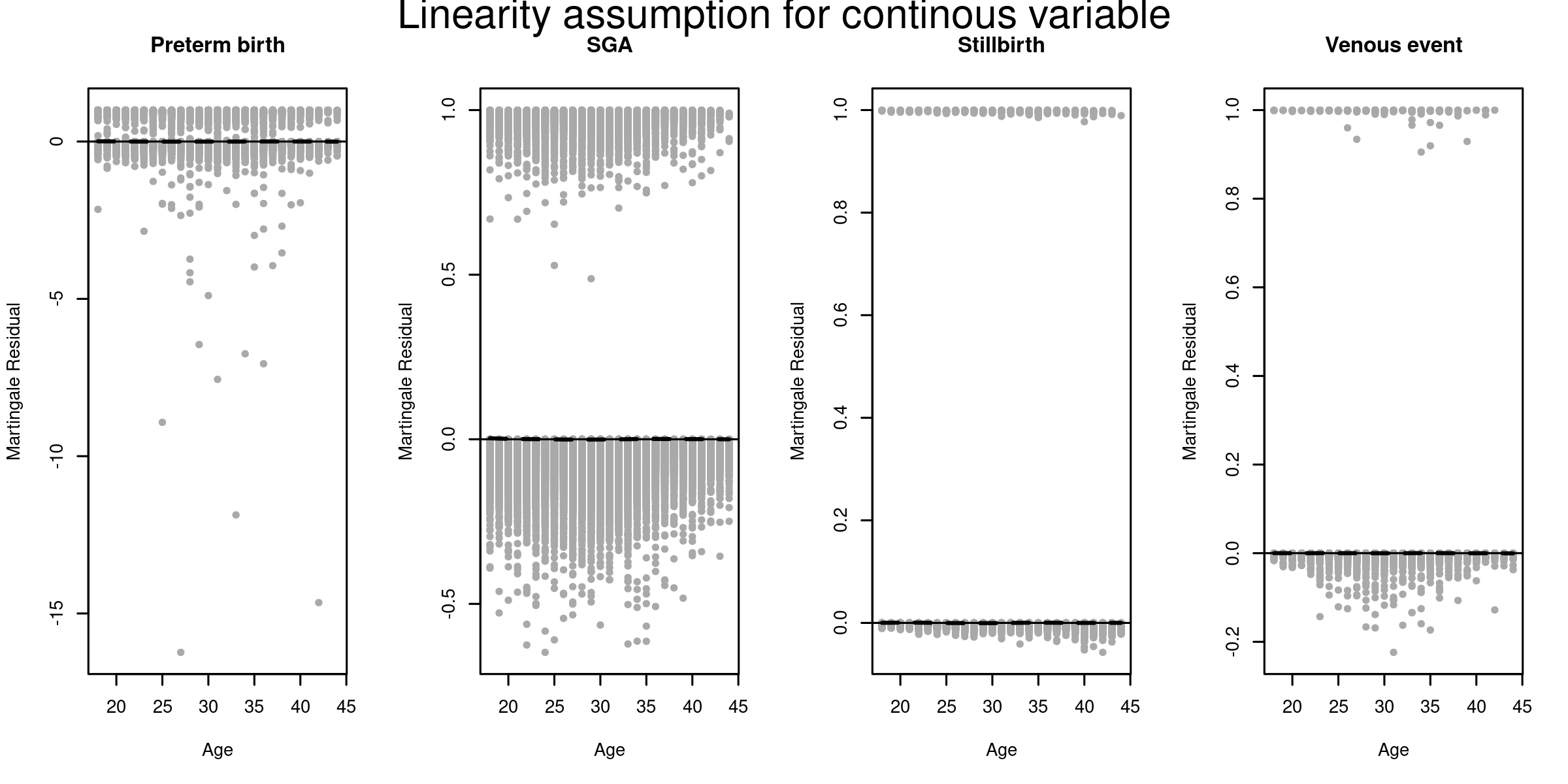


Supplementary Figure 6: Adjusted HR after relaxation of proportionality


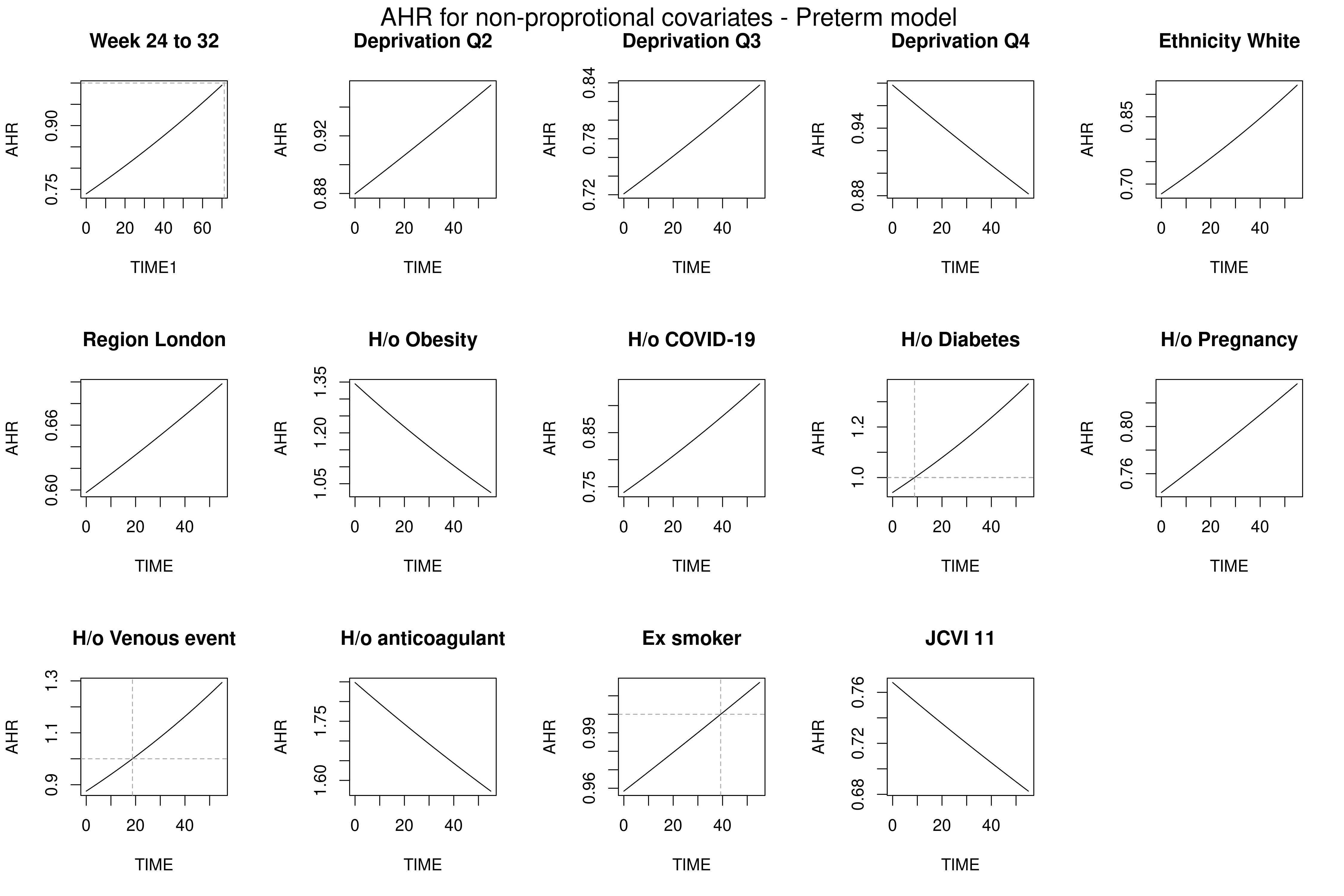


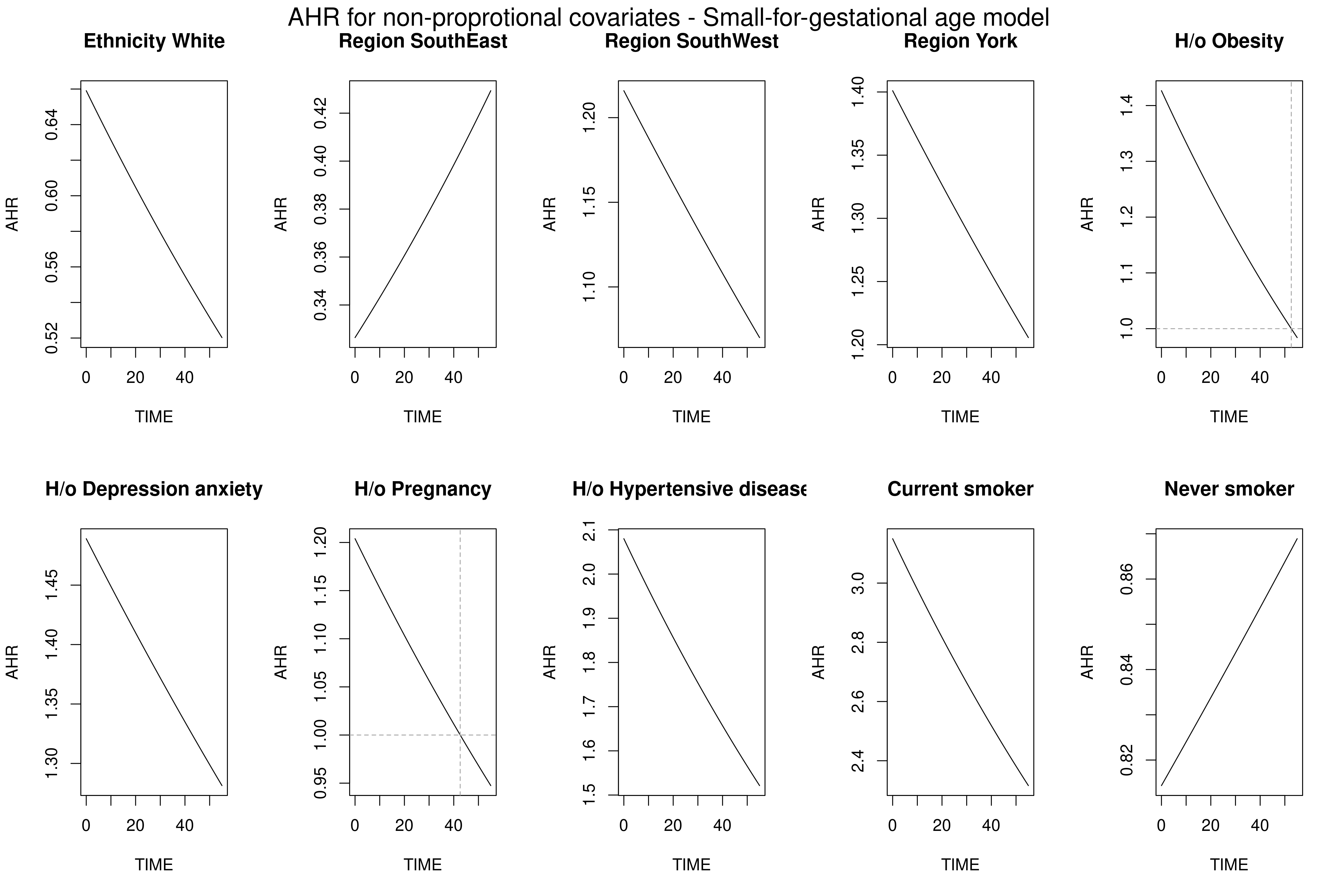


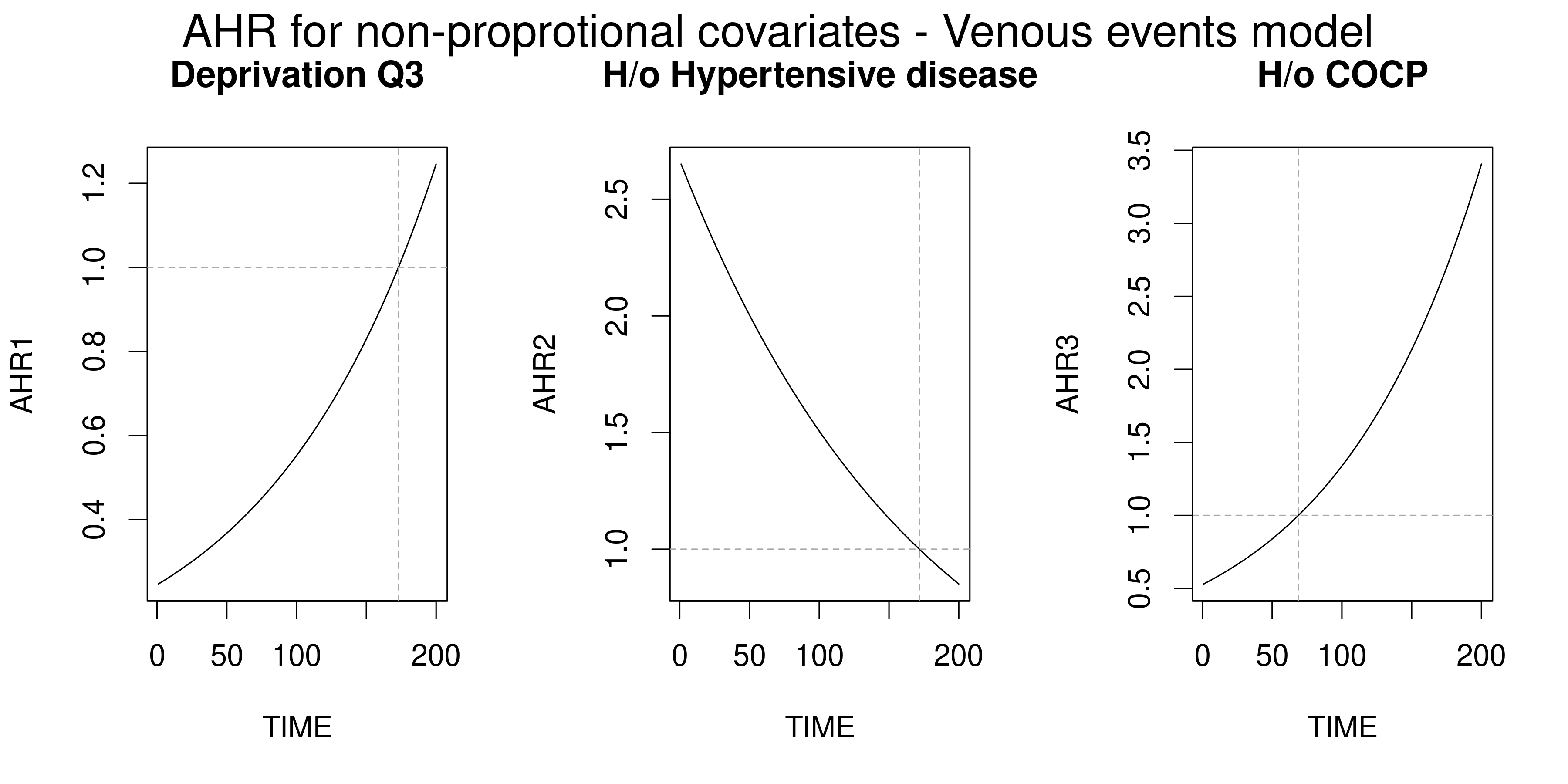


Supplementary Figure 7: Influential observations test


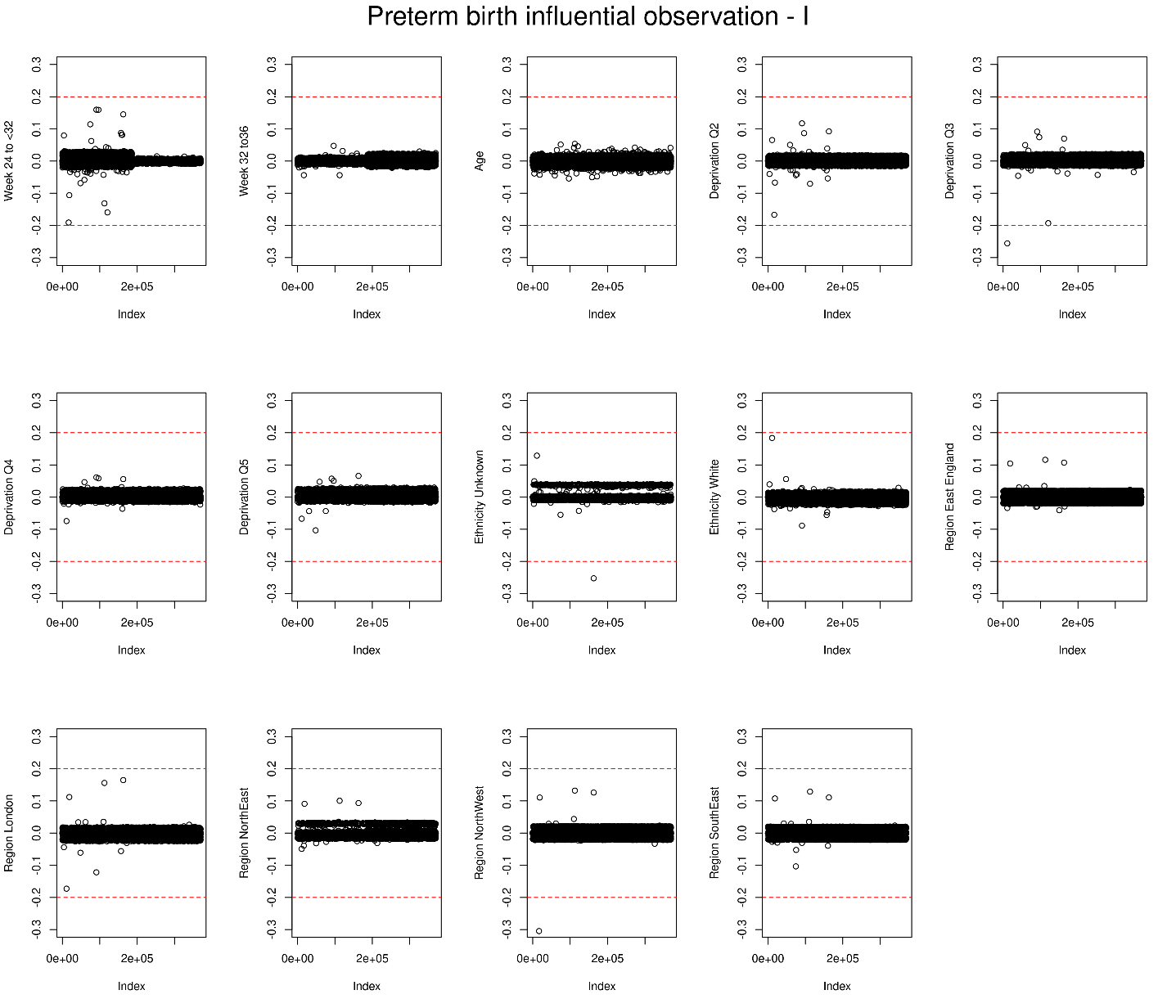


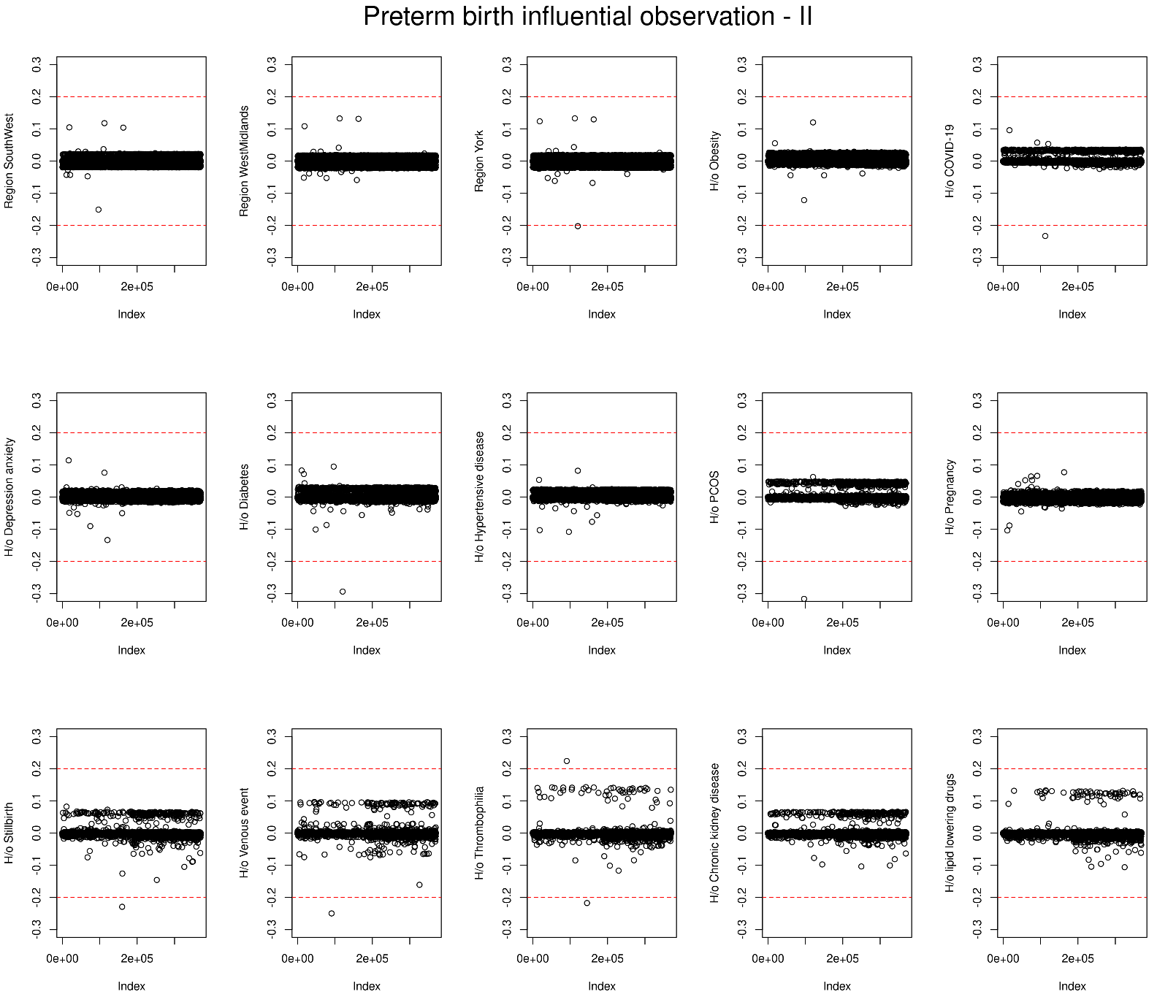


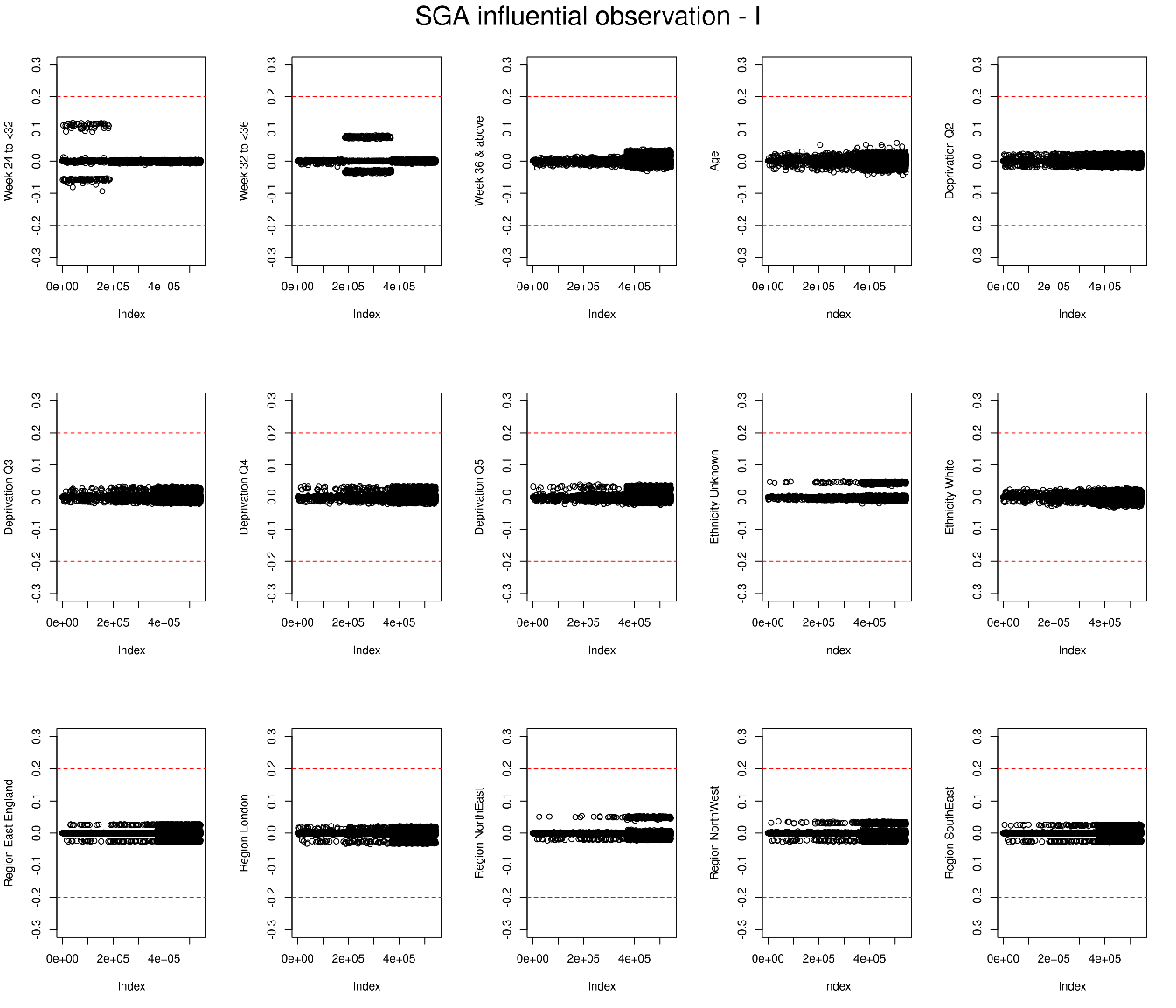


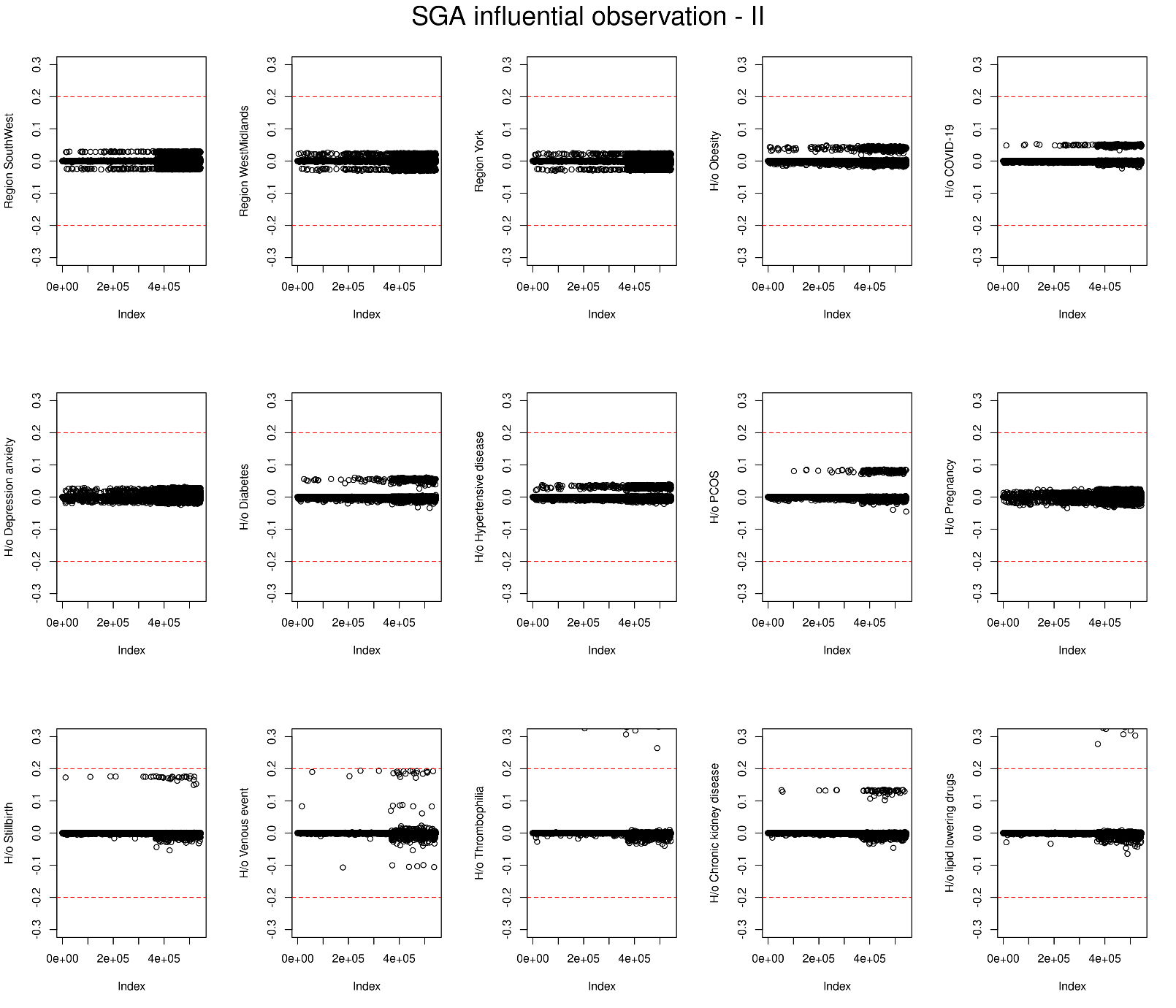


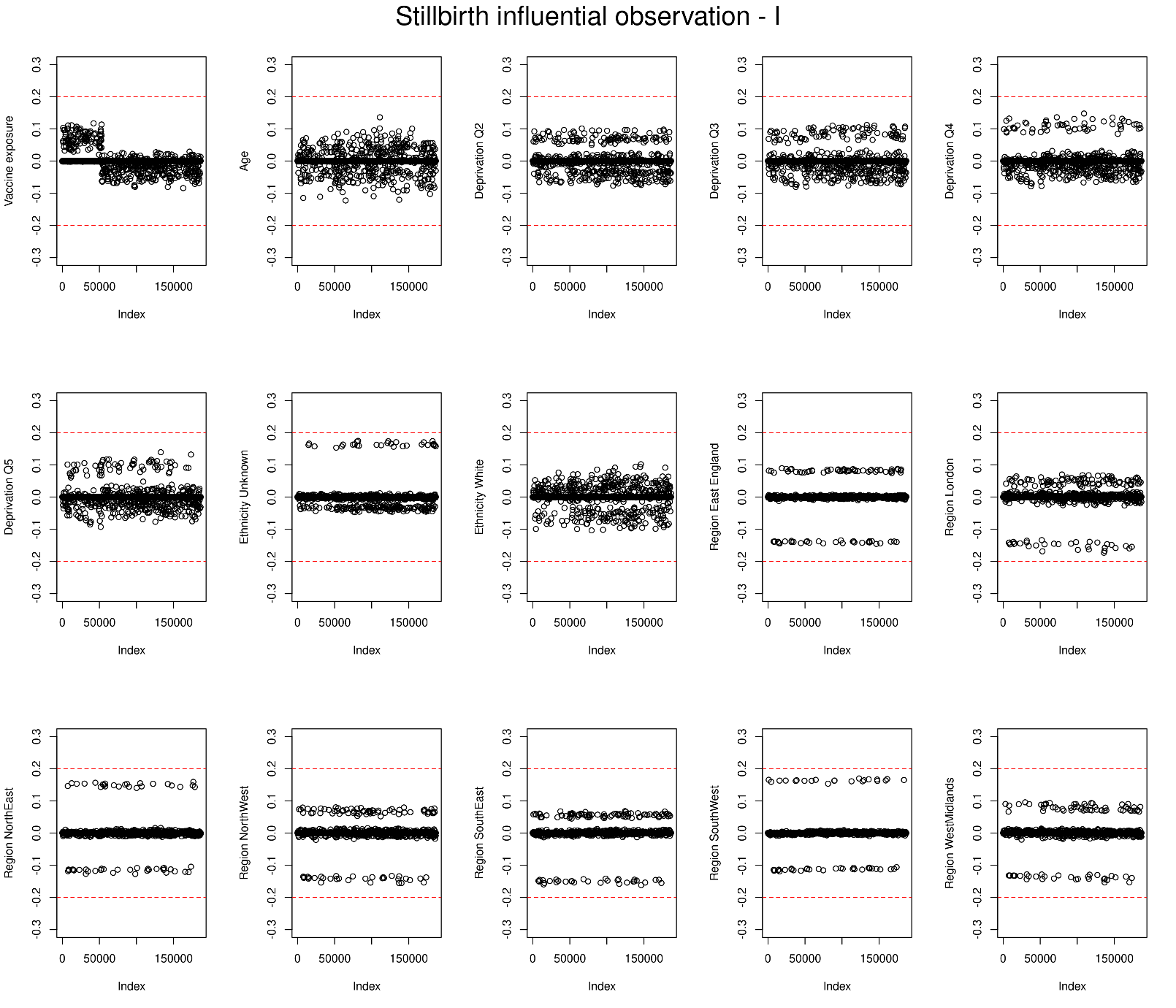


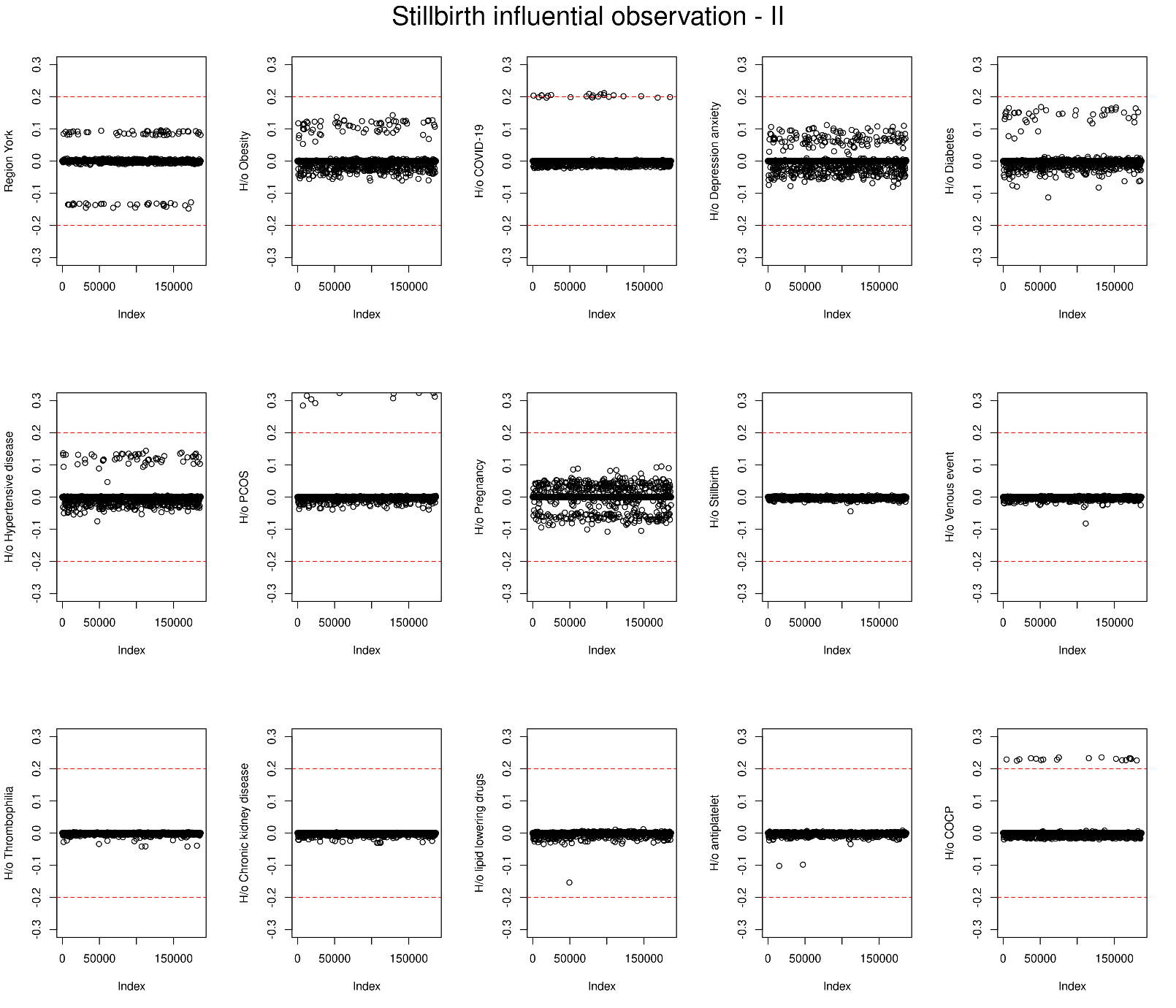


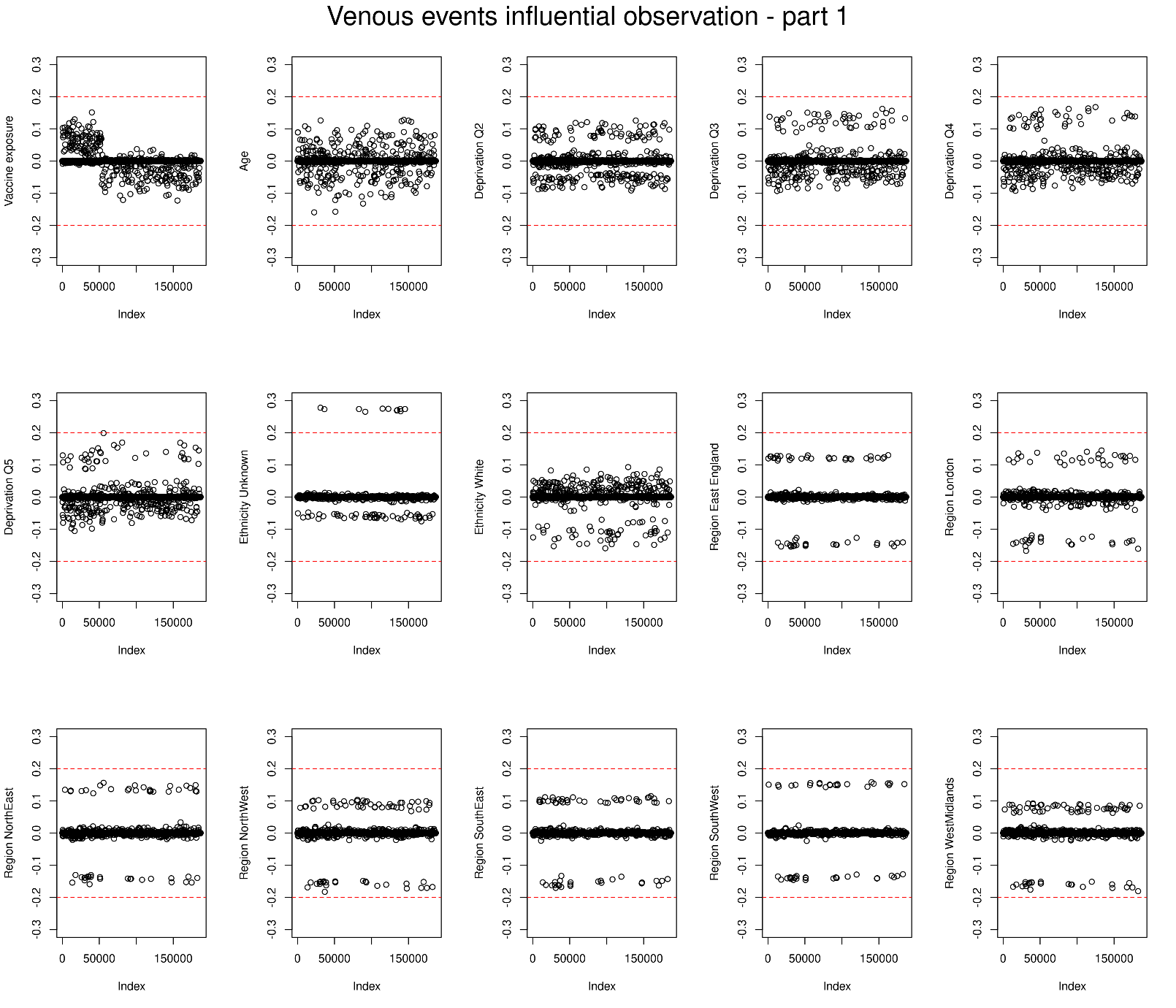


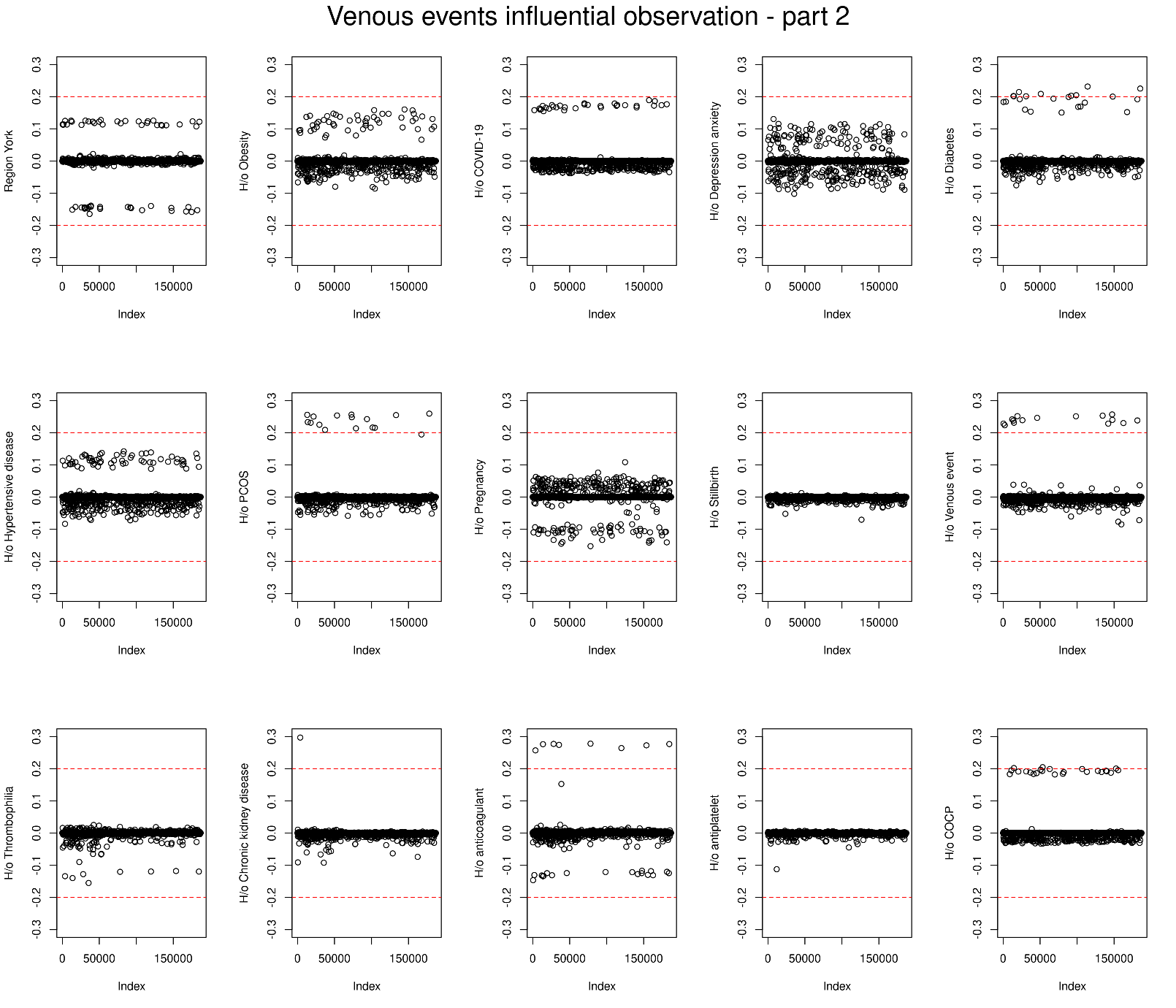

Supplement: Supplementary Material [file mmc1.docx]
